# An SSR‐based approach incorporating a novel algorithm for identification of rare maize genotypes facilitates criteria for landrace conservation in Mexico

**DOI:** 10.1002/ece3.2754

**Published:** 2017-02-10

**Authors:** Corina Hayano‐Kanashiro, Octavio Martínez de la Vega, M. Humberto Reyes‐Valdés, José‐Luis Pons‐Hernández, Fernando Hernández‐Godinez, Emigdia Alfaro‐Laguna, José Luis Herrera‐Ayala, Ma. Cristina Vega‐Sánchez, José Alfredo Carrera‐Valtierra, June Simpson

**Affiliations:** ^1^ Department of Plant Genetic EngineeringCINVESTAV ‐IrapuatoIrapuatoGuanajuatoMexico; ^2^ Unidad de Genómica Avanzada (UGA/LANGEBIO)CINVESTAVIrapuatoGuanajuatoMexico; ^3^ Universidad Autónoma Agraria Antonio NarroSaltilloCoahuilaMexico; ^4^ Instituto Nacional de Investigación Forestal Agricola y Pecuaria (INIFAP), Campo Experimental BajíoCelayaGuanajuatoMexico; ^5^ Centro Regional Universitario Centro Occidente de la Universidad Autónoma ChapingoMoreliaMichoacánMexico; ^6^ Present address: Corina Hayano‐Kanashiro, DICTUSUniversidad de Sonora. Blvd. Colosio entre Reforma y SahuaripaHermosilloSonoraMexico

**Keywords:** in situ conservation, Mexican maize landraces, Palomero, rareness algorithm, SSRs, teosinte

## Abstract

As maize was domesticated in Mexico around 9,000 years ago, local farmers have selected and maintained seed stocks with particular traits and adapted to local conditions. In the present day, many of these landraces are still cultivated; however, increased urbanization and migration from rural areas implies a risk that this invaluable maize germplasm may be lost. In order to implement an efficient mechanism of conservation in situ, the diversity of these landrace populations must be estimated. Development of a method to select the minimum number of samples that would include the maximum number of alleles and identify germplasm harboring rare combinations of particular alleles will also safeguard the efficient ex‐situ conservation of this germplasm. To reach this goal, a strategy based on SSR analysis and a novel algorithm to define a minimum collection and rare genotypes using landrace populations from Puebla State, Mexico, was developed as a “proof of concept” for methodology that could be extended to all maize landrace populations in Mexico and eventually to other native crops. The SSR‐based strategy using bulked DNA samples allows rapid processing of large numbers of samples and can be set up in most laboratories equipped for basic molecular biology. Therefore, continuous monitoring of landrace populations locally could easily be carried out. This methodology can now be applied to support incentives for small farmers for the in situ conservation of these traditional cultivars.

## 1 Introduction

Maize (*Zea mays*) was first domesticated around 9,000 years ago in Mexico (Matsuoka et al., 2002), and since then, local farmers have played an important role in both selection and conservation of specific genotypes adapted to particular environmental conditions and geographical locations. Additionally, in many cases, landraces are cultivated for their unique characteristics essential for preparation of traditional dishes. In Mexico, maize landraces are maintained (Badstue et al., 2007) by saving seed from one season to the next (Pressoir & Berthaud, 2004) and desirable genotypes are often exchanged between family members or through social alliances with both local and distant farmers or even acquired from commercial suppliers (Bellon & Berthaud, 2004; Louette, Charrier, & Berthaud,^1997^). When seed stocks are insufficient, farmers will commonly mix seed from several different sources (Bellon & Berthaud, 2004). The heterogeneous and dynamic nature of local landraces is advantageous when environmental conditions vary or infestation by pests or pathogens occurs. Although in commercial terms many landraces are nonsuitable for grain production, these varieties provide a reservoir of genes that could be exploited to develop new materials with specific adaptations (Esquinas‐Alcazar, 2005).

The introduction of commercial maize hybrids and the potential introduction of transgenic cultivars in the future have raised concerns with respect to genetic erosion of traditional landraces (Dyer, López‐Feldman, Yúnez‐Naude, & Taylor, 2014). Since the 1940s, maize germplasm resources obtained throughout Mexico have been collected and conserved ex‐situ in a number institutions including CIMMYT (International Maize and Wheat Improvement Center), INIFAP (Instituto Nacional de Investigaciones Forestales, Agrícolas y Pecuarias, UAAAN (Universidad Autónoma Agraria Antonio Narro), and UACh (Universidad Autónoma Chapingo); such populations are essentially static and do not reflect the diversity or genotype combinations currently cultivated. Some morphological and geographical data are available for these accessions, and they are currently being extensively genotyped (CONABIO http://www.biodiversidad.gob.mx/genes/pdf/proyecto/Elementos_2011_2.pdf, CIMMYT http://apps.cimmyt.org/english/docs/manual/dbases/fingerprint_Instructions_manual.htm and SINAREFI http://www.colpos.mx/redmaiz/). Several reports of the characterization of in situ landrace accessions in Mexico have also been published (Herrera‐Cabrera, Castillo‐González, Sánchez, Ortega, & Goodman, 2000; Rocandio‐Rodríguez et al., 2014; Sanchez, Goodman, & Stuber, 2000) based on morphological traits or molecular genotypes. These studies are mainly focused on particular races/varieties or limited to particular regions of the country. The contrasting results reported for different studies (Dyer et al., 2014; Sanchez, 2011) underline the complexity of determining diversity in maize landraces over large areas and under different environmental conditions.

Considering that Mexico is the center of domestication of maize, and the cultural, economic, and academic importance of this species for the country (Vielle‐Calzada & Padilla, 2009), the Mexican government, under the auspices of the CIBIOGEM (Inter‐Secretarial Commission on Biosafety of Genetically Modified Organisms), is keen to support the in situ conservation of maize germplasm by encouraging small farmers to maintain the cultivation of traditional landraces and considering incentives which would benefit the farmers who preserve the most diverse genotypes, even though these are often not commercially viable materials. The main challenges to implementing a strategy of incentives are to: (1) implement a relatively simple experimental strategy that can be easily replicated in low‐tech laboratories, but allows reliable sampling and genotyping of a maximum number of individuals while maintaining overall costs at a minimum, (2) obtain a realistic image of the existing diversity in the main regions of the country where landraces are routinely grown, and (3) identify within these samples the most uncommon or “rare” genotype combinations. Developing a strategy to meet these challenges with emphasis on supporting local farmers to maintain their traditional methods of cultivation and selection, while safeguarding the conservation of diversity within landrace populations, is the main objective of this report.

In order to meet these challenges, several genotyping methods were considered. For the proposed landrace diversity study, it was reasoned that to make the best use of resources, the priority should be the robust analysis of the greatest number of samples, in contrast to the accumulation of extensive genotype data on a few samples. Therefore, although genotyping‐by‐sequencing (GBS) methods (Elshire et al., 2011; Poland, Brown, Sorrells, & Jannink, 2012) are extremely powerful and economically relatively accessible, these methods can be time‐consuming, their exploitation implies sophisticated infrastructure and depends on highly trained bioinformatics experts, and the level of complex data generated would be a drawback rather than an advantage for the efficient conclusion of proposed diversity study. From these observations, a microsatellite (SSR)‐based strategy was developed and, by employing an information theory approach and previously obtained maize SSR data, a sampling protocol and minimum number of SSR markers were determined (Reyes‐Valdés et al., 2013).

Although several methods have been reported to identify “rare” genotypes or the smallest subset of most diverse genotypes (Gouesnard et al., 2001; Kim et al., 2007; Thachuk et al., 2009), these have been targeted at ex‐situ germplasm collections or collections assembled for breeding purposes. The range and scope of this long‐term project called for the development of a rapid and robust method of analysis, to quickly identify germplasm comprised of uncommon alleles or allele combinations and facilitate the implementation of efficient conservation strategies. Therefore, a novel algorithm was developed and tested with this aim. While developing the algorithm, it became clear that it could also be exploited to identify the minimum number of accessions needed to cover all the diversity identified in a particular sample. These materials could then be maintained with reduced storage and maintenance costs as a safeguard ex‐situ collection in the event that the in situ germplasm is lost.

The ultimate goal of the initial phase of the landrace diversity project is to analyze around 1,000 maize landrace accessions collected within the last 5–10 years from the Mexican states of Puebla, Tlaxcala, Michoacán, Oaxaca, Guerrero, and Tabasco with the aim of identifying rare genotypes and supporting decisions on the provision of incentives to small farmers and encourage the in situ conservation of maize germplasm. In order to optimize available resources, the strategy was built around the exploitation of recently obtained collections of maize germplasm kindly provided by colleagues and experts from the community of maize researchers in Mexico.

This report describes the successful testing as a “proof of concept” of the proposed experimental strategy and the development of a novel algorithm for the identification of rare germplasm based on the results obtained from the analysis of 185 accessions (comprising 5,550 individual plants) from Puebla State using 14 microsatellite loci distributed across the 10 maize chromosomes. Data generated are freely available on the project Web site: http://computational.biology.langebio.cinvestav.mx/GenoMaiz/index.html

## 2 Materials and Methods

### 2.1 Plant material

A collection of 185 maize accessions from Puebla State, Mexico, which form part of the collection of the “Proyecto Maestro de Maíces Mexicanos” (http://www.redinnovagro.in/casosexito/caso3.pdf), were analyzed in this study as a “proof of concept” that the experimental strategy and data analysis methods developed were feasible and effective. These samples were chosen because they had been recently collected (2011) and geographical and morphological data were well documented. Descriptions of the accessions and other pertinent data are presented in Table S1. Type 1 refers to the primary race classification of each accession and Type 2 a secondary, additional classification for accessions where more than 1 Race could be identified. Samples of teosinte collected by Dr. José Alfredo Carrera‐Valtierra and Palomero samples kindly provided by Dr. Ruairidh Sawers from CIMMYT stock were included as outgroups for comparison. The Puebla accessions include germplasm from 36 different maize races, and the teosinte accessions include samples from Race Chapala and Race Mesa Central, both *Z. mays* subspecies *Mexicana* and Race Balsas, *Z. mays* subspecies *Parviglumis*. Thirty‐eight seeds of each accession were sown in Sunshine^®^ substrate Mix 3 and Vermiculite Specialty GRACE^®^ in 38 square hole cell seedling starter trays in a growth chamber where temperature was maintained at 28°C with 16 hr light and 8 hr dark. Leaves from five‐day‐old seedlings were harvested for DNA extraction.

### 2.2 DNA extraction

Around 80 mg of leaf tissue was disrupted using the TissueLyser II™ (QIAGEN) system. DNA was extracted from each individual sample using the ZR‐96 Plant/Seed DNA Kit™ (Zymo Research, Irvine, CA) in 96‐well format according to the manufacturer's protocol and eluted in a final volume of 115 μl. DNA concentration was determined from observations at 260 and 280 nm using an EPOCH™ Microplate Spectrophotometer (BIOTEK^®^ Instruments, Inc.).

For each accession, DNA was obtained from 30 individual plants, and by pooling 220 ng of DNA of each sample, three bulks each composed of equal amounts of DNA (2,200 ng for each bulk) from 10 plants were formed and used to carry out the microsatellite analysis.

### 2.3 Selection of microsatellite markers

Fourteen microsatellite markers distributed across the 10 maize chromosomes were chosen based on data from a prior simulation analysis (Reyes‐Valdés et al., 2013) (Table 1). Primer sequences and the chromosome data were obtained from MAIZE GDB (Maize genetics and Genomics Database‐http://www.maizegdb.org/).

**Table 1:** List of primers used in the present study

Locus	Bin number	Repeat	Fluorescently labeled forward primer/reverse primer
phi427913	1.01	ACG	PET‐CAAAAGCTAGTCGGGGTCA/ATTGTTCGATGACACACTACGC
phi064	1.11	ATCC	PET‐CCG AATTGAAATAGCTGCGAGAACCT/ATGAACGGTGGTTATCAACAC GC
phi96100	2.00–2.01	ACCT	NED‐AGGAGGACCCCAACTCCTG/TTGCACGAGCCA TCG TAT
phi127	2.07	AGAC	NED‐ATATGCATTGCCTGGAACTGGAAGGA/AATTCAAACACGCCTCCCGAGTGT
phi053	3.05	ATAC	VIC‐CTGCCTCTCAGATTCAGAGATTGAC/AAC CCAACGTAC TCCGGC AG
phi072	4.01	AAAC	FAM‐ACCGTGCATGATTAATTTCTCCAGCCTT/GACAGCGCGCAAATGGATTGA ACT
phi093	4.08	AGCT	FAM‐AGTGCGTCAGCTTCATCGCCTACAAG/AGGCCATGCATGCTTGCAACA ATGGATACA
phi109188	5.03	AAAG	PET‐AAGCTCAGAAGCCGGAGC/GGTCATCAAGCTCTCTGATCG
phi031	6.04	CCG	PET GCAACAGGTTACATAGCTGACGA/CCAGCGTGTGTTCCAGTAGTT
phi034	7.02	CCT	VIC‐TAGCGACAGGATGGCCTCTTCT/GGGGAGCACGCCTTCGTTCT
phi051	7.06	AGG	VIC‐GGCGAAAGCGAACGACAACAATCTT/CGACATCGTCAGATTATATTG CAGACCA
phi015	8.08	AAC	FAM‐GCAACGTACCGTACCTTTCCGA/ACGCTGCATTCAATTACCGGGAAG
phi033	9.02	AAG	PET‐ATCGAAATGCAGGCGATGGTTCTC/ATCGAGATGTTCTACGCCCTGAAG T
phi96342	10.02	ATCC	NED‐GTAATCCCACGTCCTATCAGCC/TCCAACTTGAACGAACTCCTC

### 2.4 PCR amplification conditions

One primer of each pair was 5′ fluorescently labeled with one of the ABI Prism dyes: 6‐FAM, PET, NED, and VIC (see Table 1). PCR amplification was carried out in a 30‐μl volume using AmpliTaq Gold^®^ PCR Master Mix (Applied Biosystems). One hundred nanograms of template genomic DNA from each bulk was used for the PCR amplification using a GeneAmp 2600 or Veriti thermal cycler (Applied Biosystems). The conditions of PCR were as follows: 95°C initial denaturation for 5 min, 35 cycles of 95°C for 30 s, 55°C for 30 s, 72°C for 40 s, and a final extension at 72°C for 10 min. PCR conditions and the DNA concentration for the reaction mix were optimized before initiating the full‐scale analysis. All primers combinations produced PCR products within the expected size range. Before sending the products of the PCR reactions for separation on an Applied Biosystems ABI 3730XL sequencer (carried out at the Genomic sequencing facility at LANGEBIO, CINVESTAV‐Irapuato), positive controls and a selection of samples were visualized on 2% agarose gels.

### 2.5 SSR genotyping

PCR reactions for each primer pair were carried out separately and then combined to produce samples containing the four different fluorescent dyes before separation of the amplified fragments on the ABI 3730XL, using GeneScan 500LIZ as size standard (Applied Biosystems). Samples were genotyped using GENEMAPPER V. 4.0 and Peak scanner V. 1.0 software programs (Applied Biosystems).

### 2.6 Geographical localization of samples

All geographical data for the accessions were transformed to UTM using PBS software (Schnute, Boers, & Haigh, 2004) on the R environment (R Development Core Team, 2011). The geographical coordinates were encoded in.kml files and plotted in Google Earth to generate an interactive map.

### 2.7 Data analysis

The marker selection and bulk sampling scheme was developed and optimized according to Reyes‐Valdés et al. (2013). Data were collected on 185 accessions from Puebla, the main group of interest, and on 32 Palomero and 23 teosinte accessions used as outgroups. For each Puebla accession, three bulks of 10 plants were processed, for teosinte and Palomero samples bulks consisted of two plants. Data were binary scored as presence (1) or absence (0) of each allele in each of the 240 accessions, resulting in a matrix of 240 × 3 = 720 rows (batches within accession) per 278 columns (marker/allele combination). All data were captured and preprocessed into a relational database (MySQL, Oracle© 2013), and analyses were performed using the statistical environment R (R Development Core Team, 2011).

Euclidean distance and UPGMA (Unweighted Pair Group Method with Arithmetic Mean) clustering algorithm were chosen as the best alternatives for data analysis. To assess the genetic structure of the accessions, we measured the Euclidean distances between and within accession. A *t* test was performed to evaluate the average difference of distances between and within accessions. The Jackknife resampling procedure was employed to evaluate the sensitivity of the dendrogram to the exclusion of each of the marker/allele combinations.

A coefficient of rareness, *R*_i_, was estimated for each of the 185 accessions as follows: For a given accession *i*, this measure is calculated as the average of the square differences between the score of each marker/allele combination with regard to the mean score in the whole collection. Therefore, accessions with a higher average of uncommon marker/allele combinations have a higher Ri value than those that have more common alleles.

To estimate a set of accessions that include all marker/allele combinations, a looping algorithm (AMA) was developed by selecting the accession with highest *R*_i_ and including it in the selected set. Then, for each accession not in the selected set, the gain, in number of marker/allele combinations, given by each accession is measured. In the case of a tie, the accession with higher *R*_i_ value is selected. The process is repeated until all marker/allele combinations are included in the selected set. Although this procedure does not guarantee the identification of the smallest or “optimum” set, it produces results close to it. The methods used to develop Ri and AMA are described in detail in Data S1.

The relation between race and marker/allele combinations was determined by contingency analyses using the likelihood ratio test or G‐statistic. Linear regression models using various selection methods were employed to estimate the putative relationship between marker/allele combinations and meters above sea level (MASL). Details and discussion of the statistical data analysis are presented in Data S1. All data can be accessed at http://computational.biology.langebio.cinvestav.mx/GenoMaiz/index.html

## 3 Results

The geographical locations of the collection sites for the 185 maize landrace accessions analyzed in the present study are shown in Fig. S1. As can be observed, the samples were obtained throughout Puebla State and cover locations at different altitudes and with different soil types. In order to gauge the efficiency of the experimental strategy in terms of allele detection, the total number of alleles and the range of sizes of SSR alleles identified in the accessions from Puebla were compared with previous studies using the same SSRs to determine diversity in maize inbred or landrace materials as shown in Table 2. SSR marker PHI031 was the only marker used in the current study for which no previous reports were available for Mexican maize landraces. For the remaining 13 SSRs, seven presented more alleles, five presented fewer, and one presented the same number of alleles in total than had been described previously for maize landraces (Table 2). In addition, in all cases a wider range of allele sizes is reported in the current study in comparison with previous reports. Taken together, these data indicate that the experimental strategy and the SSR markers selected are informative for the material under study and have the ability to uncover new, unidentified alleles for each marker.

**Table 2:** Comparison of number of alleles and allelic range for 14 SSRs between the present study and previous publications

Marker	# alleles (Gethi et al., 2002)—IN	Allelic range (Gethi et al., 2002)—IN	# alleles (Matsuoka et al., 2002)—LR/IN	Allelic range (Matsuoka et al., 2002)—LR/IN	# alleles (Present study)—LR	Allelic range alleles (Present study)—LR
phi015	3	86–104	21/11	76–113/83–104	20	63–140
phi031	NR	NR	NR	NR	18	188–241
phi033	3	236–251	16/12	237–270/224–263	21	224–295
phi034	6	117–144	13/8	123–160/123–148	22	95–166
phi051	4	134–143	13/8	137–154/139–148	9	127–151
phi053	3	169–194	9	169–212	25	127–205
phi064	5	78–98	20/14	75–121/75–110	23	70–142
phi072	3	134–155	19/9	134–163/143–163	15	124–167
phi093	NR	NR	19/12	272–296/284–294	19	249–303
phi109188	3	164–170	17/10	148–180/148–171	22	112–182
phi127	3	112–126	10/7	105–128/112–128	11	103–131
phi427913	3	122–131	9/9	117–135/117–207	19	108–164
phi96100	3	278–296	18/11	219–301/235–300	17	233–305
phi96342	2	241–250	20/10	223–256/233–250	19	208–259

IN, Inbred line; LR, landrace; NR, not reported.

### 3.1 Comparison of genetic distances within and between accessions

**Figure 1:**
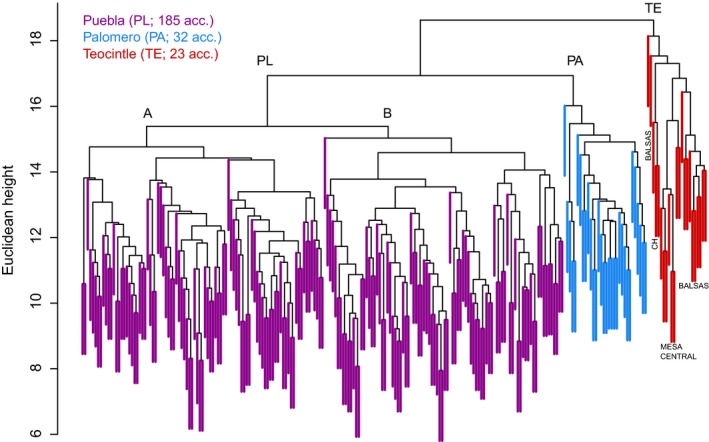
Dendrogram representing genetic diversity between samples based on Euclidean distance. Purple: Puebla (PL) samples, blue: Palomero (PA) samples, red: Teosinte (TE) samples. Subgroups of PL are denoted by A and B. CH, MESA CENTRAL, and BALSAS indicate the teosinte races Chapala, Mesa Central, and Balsas, respectively

Genetic distances between accessions from Puebla State (PL) were determined as described in Materials and Methods. Accessions of Palomero (PA), an ancient landrace, and samples of teosinte (TE), were included as outgroups for comparison. The genetic relationships for all samples are shown graphically in the dendrogram in Figure 1 and are shown to be consistent with the general geographical location and genotypes analyzed. All accessions from Puebla are placed within a single large group denoted PL and colored purple on the dendrogram. This group is further subdivided into two other compact groups, denoted A and B. The Palomero landrace samples are found in a separate clade PA (shown in blue), consistent with the observation that none of the PL accessions are classified as race PA. As expected, the teosinte samples (shown in red) form a group apart (TE) with the greatest genetic distance in relation to the landrace samples. The three distinct teosinte races: Race Chapala, Race Mesa Central, and Race Balsas, are found in different clades (denoted Ch, Mesa Central, and Balsas, respectively) within the teosinte group, supporting the consistency of the method of analysis.

Landrace populations are very variable, and we would expect some level of diversity within each accession/bulk. This is also illustrated by the data in Table S1 where around one‐third of the accessions showed characteristics of two different races (Type 1 and Type 2). Therefore, as an additional measure to demonstrate the consistency of the data presented in Figure 1, the genetic distances between pairs of bulks from the same accession and between bulks from different accessions were carried out. The results of this analysis are presented in section S1–2.2 of Data S1 and show that the distances between pair of bulks range from 7.94 to 22.45 with a mean of 15.82 and a median of 15.75 (see Table S1–6 and Figs. S1–3 in Data S1). The mean distance within accessions, 14.66, is significantly smaller than the mean distance between accessions, 17.71 (*P* < 1e−168, *t* test). The ratio of these values (17.71/14.66) is 1.21, indicating that on average the distance between bulks of distinct accessions is around 21% larger than the distance between bulks of the same accession, and this is also true for the medians of the distributions, implying that the natural grouping of the plants by accession has a solid genetic base.

### 3.2 Relationships observed between dendrogram topology geographical location and race classification

The dendrogram in Figure 1 presents clearly defined groups in relation to widely separated genotypic groups (PL landraces, PA, and TE). In order to determine whether geographical location or morphological traits were also correlated with the groupings observed, associations between maize type (race), kernel color, geographical location, and meters above sea level of geographical location were determined and the results of these analyses were superimposed on the original dendrogram.

**Figure 2:**
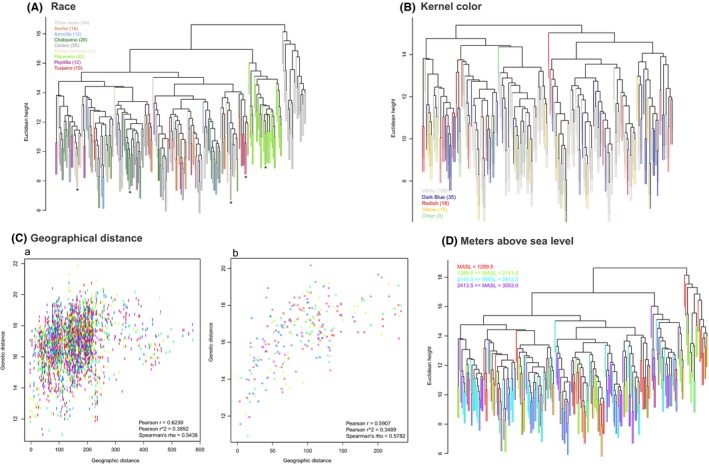
Distribution of race, meters above sea level, and kernel color in relation to genotype and relation to geographical location. (A) Relation between race and genotype; the race determined for each accession is represented by different colors overlaid on the dendrogram presented in Figure 1. The key indicates the color assigned to each race. Accessions classified as containing two different races (Type 1 and Type 2 in Table S1) are shown in gray as not classified as are the teosinte samples. (B) Relation between kernel color and genotype; the kernel color recorded for each accession is represented by different colors overlaid on the dendrogram presented in Figure 1. (C) Relationship between geographical distance and genetic distance. 2Ca represents all samples and 2Cb only teosinte samples. (D) Relation between meters above sea level (MASL) and genotype; the altitude in meters above sea level recorded for each accession is represented by different colors: red—less than 1289.5 MASL, green—between 1289.5 and 2141.0 MASL, blue—between 2141.0 and 2413.5 MASL and purple—between 2141.0 and 3053.0 MASL and overlaid on the dendrogram presented in Figure 1

Sixteen distinct maize races were identified in the PL samples (Table S1), and the mean genetic distance between races was found to be slightly higher (15.33) than within races (14.42). However, for the comparison between race and genotype, only data from races composed of at least 10 accessions were analyzed (8 including PA). The dendrogram in Figure 2A, where accessions are colored depending on their race classification, shows some association between race and genotype as indicated by * for specific clades. Additional analyses were carried out in order to determine whether specific marker/allele combinations were associated with different races; Table 3 shows that 88 marker/allele combinations were significant for the *t* test and 64 for the CT analysis and 47 were identified by both analyses. Although no single marker/allele combination was found to be significant for all accessions tested, at least one allele of marker PHI96100 was significant for each accession (Data S1). However, this marker alone was not sufficient to distinguish the different races when used individually to produce a dendrogram. The most significant marker/allele combinations for each accession are shown in Table 4.

**Table 3:** Number of accessions and number of significant (FDR ≤ 0.1%) marker/allele combinations for each one of the eight races represented by at least 10 accessions

Race	# Accessions	*t* test			CT	Both
		#Sig	#+	#‐		
Ancho	14	27	6	21	5	1
Arrocillo	12	16	2	14	3	1
Chalqueño	20	17	4	13	2	1
Conico	52	3	1	2	9	3
Elotes conicos	31	6	2	4	6	3
Palomero	32	52	13	39	53	41
Pepitilla	15	13	3	10	1	1
Tuxpeño	10	21	5	16	4	1
Total	186	155	36	119	83	52
Different marker/alleles		88			64	47

Results are presented for *t* test and contingency tables (CT) analyses. Column “Both” shows the number of marker/allele combinations significant in both tests (*t* and CT).

**Table 4:** Statistics for the most significant (smallest FDR) marker/allele combinations for each one of the races in the *t* tests

Race	Marker_Allele	In race	In others	P	FDR
Ancho	PHI9632_230	0.0000	0.5147	6.02e−17	1.54e−14
Arrocillo	PHI031_219	0.0000	0.3480	6.62e−12	1.70e−09
Chalqueño	PHI034_120	3.0000	2.6231	6.43e−12	1.65e−09
Conico	PHI96100_295	2.9423	2.3333	5.36e−10	1.37e−07
Elotes conicos	PHI015_80	0.2424	1.0597	5.21e−09	1.34e−06
Palomero	PHI96100_295	0.2500	2.8468	9.77e−23	2.51e−20
Pepitilla	PHI093_287	3.0000	2.6485	8.94e−13	2.29e−10
Tuxpeño	PHI109188_162	3.0000	2.0290	1.89e−24	4.86e−22

Average values of z are presented for the race (column “In race”) and for all other accessions in the set PL∩PA (column “In others”).

When the association between kernel color and genotype was investigated, no clear association could be observed (Figure 2B) and only 11 significant maker/allele combinations were identified for this trait (Data S1), implying that particular kernel colors are not strongly indicative of a specific race, but have probably been incorporated into different races based on cultural preferences.

Regarding the comparison between genotype and geographical distance between samples, for the Puebla and Palomero maize landraces some correlation was observed (Figure 2Ca) although the data were quite “noisy.” However, when only the teosinte (TE) samples were considered stronger, less noisy correlation was observed (Figure 2Cb), indicating an increase in genetic distance as the geographical distance increased.

Adaptation of maize cultivars to high altitudes is thought to have occurred at least in part through introgression from teosinte subspecies Mexicana. Analysis of the relationship between genotype and the MASL where the landraces were collected shows quite strong correlation (Figure 2D) where individual clades contain more accessions from either high or low elevations as indicated by * in Figure 2D. The teosinte samples also support the relationship between genotype and MASL because the Parviglumis samples were all collected at lower altitudes, whereas the Mexicana samples were collected at medium to high altitudes. The Palomero landrace has also been reported to grow at higher altitudes, and this is reflected in the Palomero clade in the dendrogram. Local farmers tend to grow landraces which have been selected locally; however, exchange and transport of seed are common, and it is likely that some genotypes related to high altitudes will have been grown at lower altitudes and vice versa and this may explain the mixture of MASL for closely related genotypes. More detailed statistical analysis (Data S1) confirmed a strong correlation between marker and allele combinations where 39% of all marker/allele combinations were significantly associated with MASL, and it was determined that around 73% of the variance related to MASL was determined by the genotype. All 14 SSR markers were shown to be associated with MASL, but a model was developed in order to determine which marker/allele combinations were most relevant (Data S1), and these results are summarized in Table 5.

**Table 5:** Coefficients and statistics for the “final model”

Marker	Allele	Estimate (β)	Std. Error	*t*−value	Pr > │*t*│
(Intercept)	α = 1,113.11		168.07	6.623	2.42e−10
PHI015	80	−126.99	38.74	−3.278	0.001205
PHI031	190	−151.06	45.4	−3.328	0.001019
	195	−150.71	45.96	−3.279	0.001202
PHI015	101	176.86	45.57	3.81	0.000135
PHI093	287	196.53	54.68	3.594	0.000398
PHI10918	145	212.5	29.9	7.108	1.44e−11
PHI96342	230	157.23	49.94	3.148	0.001858

### 3.3 Identification of rare maize genotypes based on a novel algorithm

Rare or unusual genotypes could be produced by the presence of very rare alleles, by novel combinations of alleles, or by both of these factors together. As the primary aim of the present work was to identify rare maize germplasm and provide a basis for criteria to determine priorities for conservation in situ of maize landraces, a “coefficient of rareness” (RA) and new algorithm (AMA) were developed in order to select a small set of in situ accessions that will include all marker/allele combinations present in the complete collection. A secondary function of the algorithm is to prioritize rare combinations over the most common combinations (the algorithm is described in detail in Data S1).

**Figure 3:**
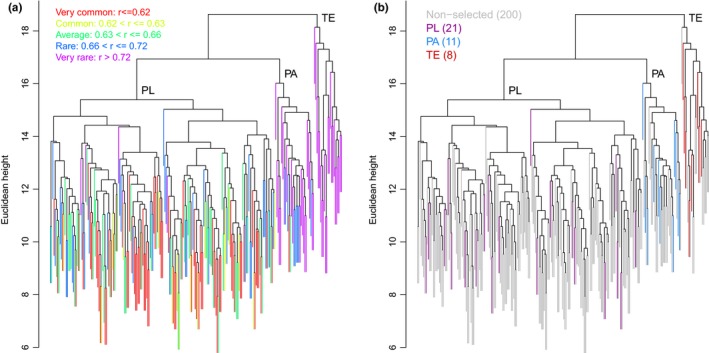
Distribution of rare genotypes and identification of a minimal collection. (a) Levels of rareness of genotypes are overlaid on the dendrogram in Figure 1; colors represent different levels of rareness as shown in key. (b) Identification of minimal collection size; gray lines indicate samples excluded from the minimal collection and colored lines the samples chosen to form the minimal collection

Based on the estimation of rareness for each accession, these data could also be superimposed on the original dendrogram, allowing the distribution of rare genotypes within the PL samples to be determined (Figure 3a). Accessions were grouped into five classes, based on their rareness coefficient: very common, common, average, rare, and very rare. In agreement with the genetic distance observed between the TE, PL, and PA samples, all TE samples were classed as very rare, whereas PA samples were classified as rare or very rare with one sample classified as common and one as very common. Regarding the PL accessions, around 77% were classified as very common, common, or average and around 20% were classified as rare and around 3% as very rare. The rare/very rare accessions are distributed throughout all 10 subgroups within the PL clade, suggesting that rare genotypes are not restricted to specific geographical regions or morphological types. This suggests that the rareness coefficient captures an element of diversity not accounted for in the cluster analysis carried out to construct the dendrogram and has important implications for defining priorities and criteria for the selection of landrace germplasm for conservation in situ. The algorithm “All Marker Alleles” (AMA) (see Data S1) was employed to define the smallest collection of samples that would account for all marker/allele combinations, including the rarest genotypes within the collection. The results of the AMA selection are shown in Figure 3b, where the optimized subsample (colored lines) contains 40 accessions including samples from PA, PL, and TE germplasm collections.

## 4 Discussion

One of the challenges related to the genotyping of maize landraces in Mexico is how to balance the experimental costs with the ability to analyze the maximum number of accessions and/or individual plants. The most effective strategy to meet this challenge is to analyze bulked samples. Similar studies employing bulks are usually based on DNA prepared from pooled leaf samples (Deputy et al., 2002; Wang, Li, & Li, 2011); however, individual extraction although more time‐consuming and expensive was shown to produce consistent results in terms of allele detection when individual and bulked samples were compared (Reyes‐Valdés et al., 2013) and was therefore the method of choice for this study. The bulking scheme has the advantage of allowing the sampling of a larger number of individuals at lower cost than individual scoring, but implies that we cannot obtain a direct estimate of the frequency of each marker/allele combination in the population sampled. The detection of a marker/allele in a bulk of ten plants implies only that at least one of the 20 haplotypes presented that combination.

Although SSR analysis may be almost completely automated, allele designation should be reviewed manually. In particular, null alleles are problematic to detect and designate because the technical failure of PCR reactions or independent mutations that alter the primer site could both lead to the lack of marker/alleles (Matsuoka et al., 2002). In this case, putatively failed PCR reactions were repeated and alleles were designated as null if the PCR reaction was repeated at least twice and consistently gave a negative result. Null alleles were identified in a proportion of around 0.49% (154 cases of 31,329 reads), and assuming that a small proportion of these nulls may be false negatives, they should not have a significant impact on the overall results and conclusions drawn from the data.

All accessions could be discriminated based on the allele data obtained, and in general, the groups in the dendrogram in Figure 1 correspond to overall differences in genotype as TE and PT form separate classes in comparison with the PL samples and race‐specific clades were formed which corresponded to the different TE races. Samples TE04 and TE23, classified as Race Balsas, are outliers within the TE group, and this may be due to the effects of maize–teosinte hybridization as has been described previously (Ellstrand, Garner, Hedge, Guadagnuolo, & Blancas, 2007; Fukunaga et al., 2005; Wilkes, 1967, 1977).

In previous reports (González Castro, Palacios Rojas, Espinoza Banda, & Bedoya Salazar, 2013), greater genetic distance (23.28) was reported between races than within a single race (0.99–8.72). Pineda‐Hidalgo et al. (2013) also reported a range of distances from 0.29 to 0.64 between accessions of the same landrace, but did not report within accession distances or distances between races. In this study, the greater genetic distances reported between rather than within accessions indicate that the data obtained are robust and consistent. Recently, González Castro et al. (2013) showed a strong relationship between genotypes, landrace types, and geographical origin based on analysis of tropical maize landraces using 30 SSRs; however, Pineda‐Hidalgo et al. (2013) were unable to find strong correlations between genotype and landraces in an analysis of landraces from Sinaloa (Mexico), based on 20 SSRs. Although a simple comparison by overlaying the classification in maize race on the dendrogram showed no strong correlation, more detailed analysis led to the identification of marker/allele combinations specifically associated with each race, opening the possibility to use these markers to support the classification of landraces into the overall maize racial classification.

The data presented here do not show a strong correlation between genotype and geographical location at least within Puebla State; this result is perhaps not surprising given the nature of the landrace populations which are highly variable and where germplasm is often transported and exchanged between farmers within a local area. In contrast, the teosinte accessions largely remain untouched and do show a strong correlation with geographical distance. Lack of correlation between kernel color and genotype probably indicates that this trait has been selected within each genotype with multiple colors associated with the genetic background of each and being the result of human selection made by ancient peoples (Doebley, Gaut, & Smith, 2006; Hanson et al., 1996).

In contrast, adaptation to high altitudes is more strongly associated with specific landraces and genotypes. Interestingly, the TE accessions from subspecies Mexicana also show the correlation with high altitudes, and this agrees well with the theory that although maize was originally domesticated in a single event from TE Parviglumis (Matsuoka et al., 2002), introgression from highland TE (Mexicana) led to adaptation of maize landraces to higher altitudes (Hufford, Bilinski, Pyhäjärvi, & Ross‐Ibarra, 2012). Additionally, markers PHI109188 (Bin 5.03) allele 145 and PHI093 (Bin 4.08) allele 287 showed a strong correlation with height above sea level. This agrees with previous reports of a QTL in the region of Bin 5.02 on the short arm of chromosome 5, associated with the trait of macrohair development thought to be important in adaptation to high altitudes (Lauter, Gustus, Westerbergh, & John Doebley, 2004) and associations between introgression on maize chromosomes 4 and 5 from teosinte Mexicana and adaptation to altitude. Our results also indicate effects related to MASL on chromosomes 8, 6, and 10 which have not been highlighted in previous studies and may depend on the particular germplasm under study.

The rareness coefficient (RA) in combination with our “All Marker Alleles” (AMA) algorithm proved to be effective in the identification of a minimum subset of accessions that covered all marker/allele combinations found in the complete collection and could also detect uncommon or rare germplasm samples and will be an excellent tool for providing criteria for selection of the minimum collection of accessions for conservation purposes. In a direct comparison with the state‐of‐the‐art algorithm for germplasm selection, “Core Hunter II” (De Beukelaer, Smy`kal, Davenport, & Fack, 2012; Thachuk et al., 2009), AMA performed favorably. AMA guarantees inclusion of all marker/allele combinations in the selected set present in the input and because it is completely deterministic gives exactly the same results every time that it is run on a given dataset. In contrast, Core Hunter II has a stochastic component, and thus, it could give different results each time that it is run on the same dataset. Also, because AMA does not need extra parameters to be run, and because it takes explicitly the rareness coefficient as objective function, it generally gives an output set with higher rareness than Core Hunter II. Also AMA is at least two orders of magnitude faster than Core Hunter II, a fact that is important for large germplasm collections. Details of the comparison are presented in section S1‐2.5.2 of Data S1.

In general, the devised strategy proved to be efficient and highly satisfactory for the low‐cost, simple, high‐volume analysis of Mexican landrace genotypes and is currently being employed to complete the long‐term goal to analyze 1,000 landrace accessions from regions in central Mexico and eventually to extend the study to cover all of the most important regions where landraces are cultivated within the country. The strategy also presents the potential for continuous monitoring of landrace populations and in particular the rarest and most endangered germplasm by resampling the same geographical regions at different time periods in the future to determine changes in the presence and distribution of specific alleles. Finally, by optimizing the sampling strategy, the method could be employed for other important native crops such as beans, peppers, *Physallis*, and *Bixa* species among many others, which may also be under threat from changes in cultural practices and land use or introduction of commercial varieties.

## Data Accessibility

Sampling locations, morphological and microsatellite data are available at http://computational.biology.langebio.cinvestav.mx/GenoMaiz/index.html. Dryad doi for the above information is doi:10.5061/dryad.c9086

## Conflict of interest

None declared.
