# Supplementary material for: An SSR‐based approach incorporating a novel algorithm for identification of rare maize genotypes facilitates criteria for landrace conservation in Mexico
Source: Ecol Evol. 2017 Feb 10;7(6):1680–90. doi: 10.1002/ece3.2754 (PMC5355182; doi:10.1002/ece3.2754)
Supplement: Supplementary file 1 [file ECE3-7-1680-s001.pdf]

Supplementary Data
(Statistical Results)

OCTAVIO MARTÍNEZ
omartine@langebio.cinvestav.mx

Contents

S1-1. Description of the data 1
S1-2. Data analysis 3
S1-2.1. Selection of genetic distance (GD) and clustering algorithm 3
S1-2.2. Dendrogram estimation and evaluation 7
S1-2.3. Estimation of allele frequencies from bulks 11
S1-2.4. Definition and estimation of *rareness* coefficient (R_i or ρ) 13
S1-2.5. Algorithm '*All Marker Alleles*' (AMA) to capture all marker/alleles in a small set of accessions 15
S1-2.6. Estimating relations between maize phenotype and genetic profile 24
S1-3. R Code 41
S1-3.1. Function to calculate the distances between and within classes 41
S1-3.2. 'All Marker Alleles' (AMA) algorithm. 44
References 48

Supplementary Statistical Results for the paper '*AN SSR BASED APPROACH INCORPORATING A NOVEL ALGORITHM FOR IDENTIFICATION OF A MINIMUM COLLECTION AND RARE MAIZE GENOTYPES FACILITATES CRITERIA FOR LANDRACE CONSERVATION IN MEXICO*'

S1-1. Description of the data

Simple sequence repeats (SSRs) are markers that could present up to two distinct aleles per locus in an individual. Alleles for these markers are characterized and denoted by their number of bases pairs (bp). A previous work in our research group determined, based in an information theory approach, that three bulks of 10 plants per accession give near optimal allele detection with low genotyping error [27]. In this work we employed 14 SSR markers (see Table S1-1) scattered in all 10 maize chromosomes to characterize genetic diversity. Briefly, for each one of the bulks, the equimolar mixture of DNA individually extracted from the 10 plants was subjected to PCR with the primers corresponding to each SSR. Alleles for each marker were determined, and binary scored (zero for absence, one for presence) for each one of the marker/allele[1] combinations. This information is stored in in the table '`fingerprint`' of the GenoMaiz database.

Additionally to the alleles directly found, a 'null' alele was determined for a bulk when no alleles were found for a given marker (i.e., the PCR reaction repeatedly failed to amplify any product), and the quality of the DNA was assured by amplification of other markers in the sample.

The vector of binary scored marker/allele combinations for each bulk within each accession is considered here as a '*genetic profile*' for the corresponding bulk (table `fingerprint` in GenoMaiz database); variable names in this table have names corresponding to `<Marker>_<allele>`; thus, for example, variable `PHI015_80` contains the binary score for the marker allele 80 of the marker PHI015 in each one of the bulks within accessions. By adding the vectors of the genetic profiles of the three bulks of one accession,

[1] With 'marker/allele' we mean '*a specific allele of a given marker*'; the ordered vector of all marker/allele combinations form a genetic profile or '*fingerprint*'.

one obtains a vector with the same variables than the individual genetic profile of the bulk, but now each variable can take values, 0, 1, 2 or 3, corresponding to the number of bulks that was scored with a value of one. These data give a semiquantitative assessment of the frequency of the corresponding marker/allele combination in the accession. The genetic profiles for accessions are kept into the table '`fingerprint_acc`' of the GenoMaiz database.

Additionally, for each accession the table '`accession`' of the GenoMaiz database includes information about the place where the accession was collected and is currently grown (name of the locality, geographic coordinates and height above sea level), 'type' of maize (an agronomic classification), plus kernel color. Also, photographs showing kernels for each accession are available here.

All analyses presented here were performed with data in the tables `fingerprint`, `fingerprint_acc` and `accession` of the database. A full MySQL dump of the database can be downloaded from DocGeno-Maiz.

Table S1-1 presents the range, number and proportions of alleles for each one of the 14 SSR markers in the collection formed by accessions from the groups Puebla (PL), Palomero (PA) and Teosinte (TE).

TABLE S1-1. Number and percentages of accessions studied from Puebla (PL), Palomero (PA) and Teosinte (TE); the full set corresponds to the column 'All' (set PL ∪ PA ∪ TE). Range (bp), number (n_a) and proportion (p_a) of alleles found per marker in each set. The proportion, p_a, at each column (set) is with reference to the alleles found in the corresponding marker (n_a in column 'All').

Collection ⇒		All	PL∪PA		PL∪TE		PA∪TE		PL		PA		TE	
Number of Accessions ⇒		240	217; 90%		208; 87%		55; 23%		185; 77%		32; 13%		23; 10%	
Marker	Range	n_a	n_a	p_a	n_a	p_a	n_a	p_a	n_a	p_a	n_a	p_a	n_a	p_a
PHI015	63–119	17	15	0.88	16	0.94	17	1.00	14	0.82	15	0.88	16	0.94
PHI031	188–241	19	19	1.00	19	1.00	18	0.95	19	1.00	16	0.84	15	0.79
PHI033	224–288	21	19	0.90	20	0.95	19	0.90	18	0.86	13	0.62	17	0.81
PHI034	95–166	23	22	0.96	22	0.96	17	0.74	21	0.91	13	0.57	15	0.65
PHI051	127–151	10	9	0.90	10	1.00	8	0.80	9	0.90	7	0.70	8	0.80
PHI053	127–205	26	19	0.73	25	0.96	24	0.92	17	0.65	17	0.65	9	0.35
PHI064	69–151	35	35	1.00	28	0.80	23	0.66	27	0.77	23	0.66	16	0.46
PHI072	121–167	17	16	0.94	16	0.94	17	1.00	15	0.88	15	0.88	16	0.94
PHI093	249–303	18	12	0.67	17	0.94	17	0.94	11	0.61	10	0.56	15	0.83
PHI109188	112–182	22	22	1.00	22	1.00	16	0.73	22	1.00	12	0.55	15	0.68
PHI127	103–153	14	14	1.00	12	0.86	13	0.93	12	0.86	13	0.93	10	0.71
PHI427913	108–145	16	15	0.94	16	1.00	15	0.94	14	0.88	14	0.88	15	0.94
PHI96100	226–305	20	20	1.00	18	0.90	16	0.80	17	0.85	14	0.70	12	0.60
PHI96342	208–259	20	20	1.00	20	1.00	18	0.90	20	1.00	15	0.75	17	0.85
Totals for n_a, p_a:		278	257	0.92	261	0.94	238	0.86	236	0.85	197	0.71	196	0.71
Ratio Alleles per Accession		1.20	1.18		1.25		4.33		1.28		6.16		8.52	

From Table S1-1 we can see that all 14 SSR markers showed high levels of polymorphism, with a number of alleles per marker that goes from a minimum of 10 for PHI051 up to a maximum of 35 for PHI064, with a total of 278 alleles for all markers. We can use the 'Ratio Alleles per Accession' (RAA) i.e., Number of alleles / Number of accessions (last row of Table S1-1) as a relative –and preliminar, measure of genetic diversity. In the full collection (column 'All') such ratio is $278/240 \approx 1.20$, but the richest set is by far the TE set, with an $RAA \approx 8.52$, which is approximately 6.7 times larger than the RAA for PL (1.28) and 1.4 times larger than the RAA for PA (6.16). This results are consistent with the fact that the TE set represents 3 different types of teosintes, ancestors of maize and wild plants, while PA accessions –the second richest single group (RAA=6.16) represent an ancient maize race. Obviously, the RAA coefficient depends on the relative sample sizes employed; the PL set, with 185 accessions represents 77% of the

complete sample, while PA and TE, with 32 and 23 accessions represent only 13 and 10% of the complete sample, and the rate of discovery of new alleles decreases with sample size.

To make a fair comparison between the RAA diversity index, we obtained 1000 independent random samples of $n = 32$ or $n = 23$ accessions from the PL dataset, evaluating how many different alleles were found each time and calculating the values of RAA at each independent sample. With this procedure we can approximately, but fairly, evaluate if the values of RAA found in PA and TE, $RAA_{PA} = 6.16$, $RAA_{TE} = 8.59$ –with $n = 32$ and $n = 23$ accessions respectively, indicate a larger allelic diversity than the one found in PL, $RAA_{PL} = 1.18$ with the original $n = 185$ accessions. Table S1-2 presents the results form this resampling procedure.

Table S1-2. Statistics for values of RAA obtained by sampling $n = 32$, $n = 23$ accessions at random from the PL accession. The random sampling was repeated 1000 times. LL and UL are the lower and upper limits for the 95% confidence interval calculated.

Reference	Sample	Median	Mean	S	LL	UL
PA: 6.16	$n = 32$	5.969	5.966	0.1982	5.5617	6.3125
TE: 8.52	$n = 23$	7.870	7.861	0.2858	7.3043	8.3913

From Table S1-2 we can see that the mean and median values of RAA obtained with samples of $n = 32$ or $n = 23$ PL accessions are, as expected, much larger than the value obtained with the original sample size in PL, $RAA_{PL} = 1.18$ with $n = 185$. Even when the means and medians of RAA in the PL samples of sizes $n = 32$ and $n = 23$ are smaller than the reference values in PA and TE, $RAA_{PA} = 6.16$, $RAA_{TE} = 8.59$, only the value for TE, $RAA_{TE} = 8.59$ is above the approximate 95% confidence interval for the RAA coefficient for samples of size $n = 23$ from the PL accessions (7.3043 to 8.3913). From this we can conclude that there is evidence that the TE accessions have larger diversity than the PL accessions, but the diversity in PA and PL can be considered approximately equal.

From this analysis of allele diversity in the three groups of accessions, we can conclude that there is abundant genetic information to investigate the diversity and relations of these plant populations.

S1-2. Data analysis

Inference about germplasm diversity and genetic relationships among accessions for conservation proposes, needs a careful choice of genetic distance measure and clustering procedure [23]. There is a plethora of both, genetic distance measures and cluster algorithms [8].

The particular nature of the data in this work, generated by sampling for each accession 3 bulks of 10 plants [27], demands to test and validate the selection of genetic distance as well as clustering procedure to assure the objective determination of genetic diversity and relationships between the materials. We discuss the estimation of allele frequencies for this kind of data in section S1-2.3.

All statistical analyses were performed in R [25], and the code is presented in the appendix of this document or it is available from us upon request.

S1-2.1. **Selection of genetic distance (GD) and clustering algorithm.** Genetic distance (GD) was defined by Nei [24] as '*that difference between two entities that can be described by allelic variation*' [24]. The estimation of GD depends on the data at hand and can be performed by a variety of measures (see for example [23]). The binary scoring of the bulk profile in our data implies some decrease in genetic information; instead of knowing the exact genotype of a plant, the presence of '1' in a bulk of ten plants for a particular marker/allele combination tell us that '*at least one allele* (for that marker/allele) *was present*'. This information reduction produces a bias in the estimation of allele frequencies (see S1-2.3), and restricts the application of standard GD estimators, making it necessary to evaluate alternatives for its estimation.

Various GD estimators are available for binary scored data (reviewed in [23]). The variations in GD estimators arise from the way in which a coefficient takes into account the differences in the binary scored data. Euclidean distance is defined as $GD_E = \sqrt{\sum_i (x_i - y_i)^2}$, where here $x_i,\ y_i;\ i = 1, 2, \cdots, n$ are the binary scores (1 or 0) for each one of the marker/allele combinations. GD_E takes into account and gives the same weight to all differences found, and can be normalized to give values in the interval $[0, 1]$ by a factor $1/\sqrt{n}$ –given that $\sqrt{n}$ is the absolute maximum of GD_E for binary scored data. An estimator related to GD_E is the *simple matching coefficient*, due to Sokal and Michener [28] and equal to $GD_{SM} = (\sum_i |x_i - y_i|)/n$. It is clear that for binary scored data $nGD_{SM} = GD_E^2$, and the not-normalized version of GD_{SM}, say $GD_M = \sum_i |x_i - y_i|$, is called the *Manhattan* or *taxi driver* distance [2]. In contrast with the normalized versions of GD_E and GD_M, which take into account the cases where an allele is absent in both individuals –the $x_i = 0,\ y_i = 0$ cases, the Jaccard's coefficient [18] does not take into account such cases and thus the formula to estimate genetic distance is given by $GD_J = (\sum_i |x_i - y_i|)/(\sum_i x_i + \sum_i y_i - \sum_i x_i y_i)$. Other coefficients to measure GD, as the ones due to Nei an Li or Rogers (see [23]) give different weights to the differences or transform the scale of measure.

The measure of the differences in allele frequencies between and within populations has been a classic way to estimate the amount of genetic differentiation between populations and in subdivisions of a population (see for example [35, 4]). Our data do not provide direct estimators of allele frequencies, in fact, we have only 3 binary scored genetic profiles (fingerprints) for each accession. However, accepting that each one of the accessions represents a population, we can measure the GD between and within accessions. To chose the measure of GD we can select the coefficient that gives a more robust and clear segregation of the accessions when taking into account the *GD within* accession, i.e., between each pair of bulks samples, when compared with the distances estimated *between* accessions, i.e., between bulks of plants that belong to different accessions.

With the aims of estimating and testing the distances between and within genetic profiles, we programed an R function (`dist.bet.wit`; see section S1-3.1 for the code). `dist.bet.wit` has as arguments `x`, the matrix of profiles, `method` to select the measure of distances and `classes`, a vector signaling to which class (accession or group) each individual belongs. Additionally, the function allows the scaling of the distance by the maximum found in the set analyzed. The function produces a list that includes the matrix of distances, the vector of distances between and within classes, statistics for the distances within each class and two tests (the t-test and the non-parametric Wilcoxon rank test) for the hypothesis of equality of means for the distances between and within classes.

The more relevant and different estimators for GD in the case of binary scored genetic profiles are the Euclidean and Jaccard's distances; the majority of other estimators are a transformation of these measures. Table 3 presents statistics for the distances between the 720 genetic profiles, classified in the sources 'Between' or 'Within' accessions and calculated by either, the Euclidean or Jaccard formule and normalized by their corresponding maximum. A total of $750 \times (750-1)/2 = 258840$ genetic distances exist between all distinct pairs formed by the 750 genetic profiles. A genetic distance say, $d(r, s)$ is classified as 'Between' if the genetic profiles r and s belong to different accessions, and as 'Within' if r and s belong to the same accession. Because we have 240 accessions (set PL $\cup$ PA $\cup$ TE; see Table S1-1) and there are three individual genetic profiles (bulks) per accession, we have $3 \times 240 = 720$ distances 'Within' accessions, and the remaining distances, $258840 - 720 = 258120$, are distances 'Between' accessions. To complement the results presented in Table S1-3, Figure S1-1 shows the distributions –as box plots, for the distances 'Between' and 'Within' accessions calculated by the Euclidean or Jaccard estimator.

From Table S1-3 we can see that the distribution of all the Euclidean distances have a smaller range, interquartile range and standard deviation (S) than the case of the Jaccard measures ('Total' rows in Table S1-3). This is partially a result of the fact that Euclidean distance is the square root of the sum of squares, but the fact that the Euclidean distance does not depends on the variable number of cases where both profiles have a pair of ones –as does the Jaccard coefficient, also influences this difference. The determination coefficient, R^2 –the square of the Pearson correlation coefficient, between the distances are $0.8480, 0.8464$ and 0.8504 for the 'Total', 'Between' and 'Within' sources in Table S1-3, indicating that

Table S1-3. Statistics for the normalized genetic distances obtained by the Euclidean and Jaccard measures in 720 genetic profiles corresponding to 240 accessions (set PL ∪ PA ∪ TE). Distances segregated in the 'Between' and 'Within' accession sources.

Distance	Source	n	Min.	1st Qu.	Median	Mean	3rd Qu.	Max.	S
	Between:	258120	0.3888	0.7071	0.7548	0.7546	0.8069	1.0000	0.0738
Euclidean	Within:	720	0.3735	0.5498	0.6004	0.6024	0.6559	0.7850	0.0755
	Total:	258840	0.3735	0.7071	0.7548	0.7542	0.8069	1.0000	0.0743
	Between:	258120	0.2471	0.6256	0.6955	0.6939	0.7684	1.0000	0.0974
Jaccard	Within:	720	0.2407	0.4299	0.4977	0.5028	0.5735	0.8125	0.1008
	Total:	258840	0.2407	0.6256	0.6953	0.6934	0.7679	1.0000	0.0980

approximately 85% of the variation in one of the measures can be explained by the other and *vice versa*.

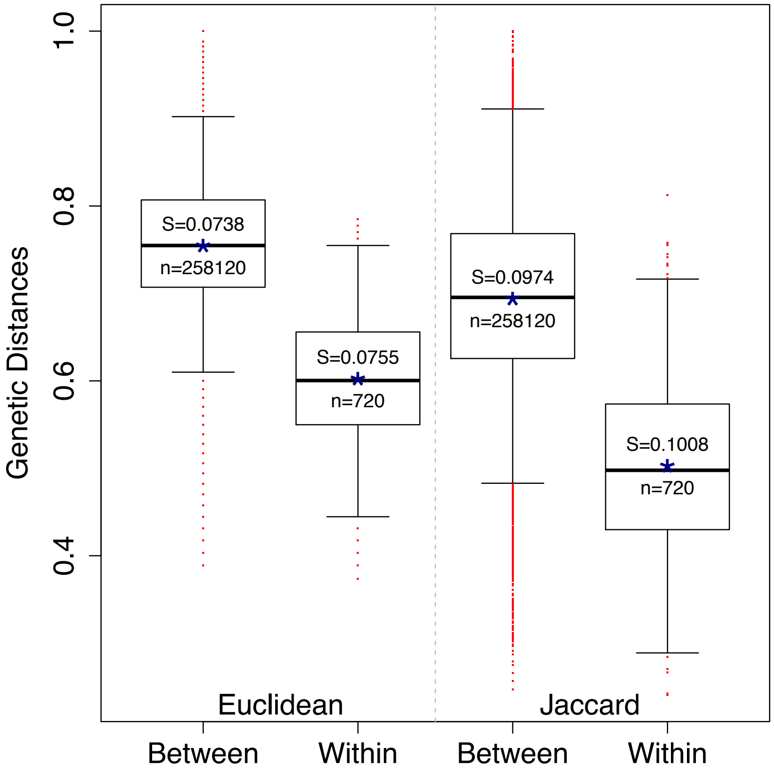

Figure S1-1. Distributions (as box plots) of genetic distances between and within accessions estimated by two measures, Euclidean and Jaccard's or 'binary'. Medians are shown as broad lines, while means are marked by asterisks. Bottom and top of the boxes are from the first to the third quartiles. Boxplot whiskers extend 1 interquartile range unit from the boxes. Outliers are shown as red points. Number of observations (n) and standard deviations (S) are shown within the corresponding boxes.

In Table S1-3 and Figure S1-1 we can appreciate the differences of the distributions of the *GD* 'Between' and 'Within' accesions, calculated from the individual genetic profiles (bulks of ten plants). For both measures, Euclidean and Jaccard, the distances 'Between' have a larger central tendency (mean and median). The differences between the means of the 'Between' group are significantly larger than the corresponding values in the 'Within' group ($P < 1 \times 10^{-200}$; Wilcoxon and T-test). With this we confirm that both measures are detecting the structure 'genetic profiles within accession' that exist in the data.

However, Euclidean distances present smaller variation than the Jaccard's measure, and this is reflected in smaller standard deviation (S), range and interquartile range for the 'Between', 'Within' and 'Total' groups (Table S1-3 and Figure S1-1). Even more, appart from being more compact –and thus, precise, the Euclidean distance present much less outliers (data 1.5 interquartile units away from the median; red points in Figure S1-1) than the Jaccard distance. Additionally, the statistic for both, the Wilcoxon and T test are smaller for the Jaccard distance than for the Euclidean ($W_E = 171850000, W_J = 168910000; t_E = 54.039, t_J = 50.804$) indicating a larger power of the tests (performed for the same data but with different measures). Also, the length of the 95% confidence intervals for the differences between the means (from the T test) are 0.0110 for Euclidean and 0.0148 for Jaccard, indicating more precision in the Euclidean than in the Jaccard measure of GD. These characteristics of the distributions make the Euclidean distance a better choice than the Jaccard measure for the evaluation of GD in this data.

In measuring GD between two genetic profiles, we use vectors of marker/allele combinations. This implies that markers which present more alleles are given more weight when estimating GD ('*equal weight to each allele*' strategy). However, each marker represent a locus in the maize genome, and thus it can be argued that each marker (locus) must be given the same weight, independently of the number of alleles found. We investigated this possibility using independent distance matrices, each one obtained from data of a single marker, and then obtaining a 'representative' matrix of distances as the average of the distances from the individual markers ('*equal weight to each marker*' strategy). The recovery of known topologies (bulks within accessions) with the '*equal weight to each marker*' showed a poorest performance than our original strategy (*equal weight to each allele*), and thus the possibility of using an average distance giving equal weight to each marker was discarded (data not shown here but see our InternalReport2 [2])

Having selected the Euclidean distance in genetic profiles as the measure of GD, we need to decide which agglomeration method (clustering) to use in the construction of a dendrogram that better reflect the genetic relations between accessions. Among possible choices for the clustering algorithm are 'Ward", 'Single linkage', 'Complete linkage', 'Average' (or UPGMA [3]), 'mcquitty' (or WPGMA), 'median' (or WPGMC) and 'Centroid' (or UPGMC) [8]. These methods differ, basically in the way that clusters with more than two individuals are grouped to form larger clusters. For example, 'Single linkage' uses the minimum distance between pairs of individuals in the clusters, while 'Complete linkage' uses the maximum and UPGMA employs the average, etc.

To select the best clustering method for our data we employed an heuristic test under the assumption that the genetic profiles obtained from bulks of the same accession will be closer among them in GD than to genetic profiles obtained from bulks of other accessions. Even if this assumption is not correct in all cases, because, for example two accessions could be genetically very close, i.e., not genetically differentiated in 'many' or 'most' of the cases the GD within accessions are in fact smaller than between accessions (see Table S1-3 and Figure S1-3). In this case we employed the 555 genetic profiles from the 185 Puebla (PL) accessions, and programed an R function ('`find.clust3`', available upon request) to calculate the number of clusters of three elements that were formed by genetic profiles from the same accession ('*uni-accession*' clusters, or clusters containing elements only for a single accession). Table S1-4 shows the results of running the function `find.clust3` with clusters obtained with the R function `hclust` under seven clustering methods.

From Table S1-4 we can see that there is an ample variation between the number of uni-accession clusters recovered per method, that goes from 5 for the 'Centroid' method up to 28 for the 'Average' (UPGMA) method. In percentage with regard to the maximum possible number of uni-accession clusters, 185, this represents a variation within 3 and 15%. This variation give us a relative measure of the differences in topology caused by the selection of clustering algorithm. Apparently the percentage of uni-accession clusters is low, but we need to take into account the enormity of the space of all possible bifurcating dendrograms. For example, the number of distinct three elements sets which can be formed with 3 distinct

[2] 'Internal Reports' from the Computational Biology Lab are informal publications which are made pubic only after the publication of the results in the corresponding project.

[3] *Unweighted Pair Group Method with Arithmetic mean*

Table S1-4. Number and percentages of clusters of three elements with all bulks from the same accession (*uni-accession* clusters) found per method. Figures in the main diagonal and above are the number of uni-accession clusters found by the method and the number of those shared by both methods (upper triangular), respectively. Figures below the diagonal (lower triangular) are percentage of clusters shared by the two methods with reference to the maximum (185). Last row (%Method) gives the percentage found by method in relation with the maximum (185).

**Method**	Ward	Single	Complete	Average	Mcquitty	Median	Centroid
Ward	**24**	12	21	23	23	13	4
Single	6%	**13**	13	13	13	11	4
Complete	11%	7%	**27**	23	23	14	4
Average	12%	7%	12%	**28**	25	15	4
Mcquitty	12%	7%	12%	14%	**26**	15	4
Median	7%	6%	8%	8%	8%	**15**	4
Centroid	2%	2%	2%	2%	2%	2%	**5**
%Method	**13%**	**7%**	**15%**	**15%**	**14%**	**8%**	**3%**

elements in our case equals $555{*}554{*}553/3! = 28,338,485$ thus the probability of obtaining a uni-accession cluster at random, i.e., without having the subjacent genetic structure, is around $185/(555{*}554{*}553/6) = 6.5e-06$ In Figure S1-1 (Euclidean distance) we can see that there is an overlap in the ranks of the distances 'Between' and 'Within' accessions, explaining why we have a relative small number of uni-accession clusters; in other words, the accessions from the PL group even when present some degree of structure are not completely segregated in their genetic profiles.

The maximum number of uni-accession clusters recovered is 28 for the 'Average' (UPGMA) method, close to the number recovered by the 'Complete' linkage, which recovers 27 (Table S1-4); however only 23 of these uni-accession clusters are shared by both methods. This demonstrates that distinct methods recover distinct uni-accession clusters. Based on this results we selected the 'Average' (UPGMA) for the construction of the dendrograms.

S1-2.2. **Dendrogram estimation and evaluation.** We are interested in studying the genetic relation of our marker/allele data at accession level. With this aim, and as mentioned earlier, we summarize the genetic information for each accession by adding the three individual marker/allele profiles from individual bulks. These vectors are kept in the table `fingerprint_acc` of the database. Given the addition of the three genetic profiles from the bulks, each marker/allele combination in the genetic profiles for accession can take the values 0, 1, 2 or 3, providing a semi-quantitative measure for the allele frquency[4].

To estimate, show and validate the genetic relations between accessions of the PL, PA and TE groups, we first obtained the Euclidean distance between the accession's genetic profiles using the R function `dist` with parameter `method = "euclidean"`, and to obtain the corresponding dendrogram we used the UPGMA (or 'average' method) using the R function `hclust` with parameter `method = "average"`. To color the dendrogram by group we used the function `ColorDendrogram` of the R package 'sparcl' [34].

The bootstrap resampling procedure [7] consists in taking B samples with replacement from the data and calculate the statistic of interest for each one of the samples, with the aim of estimating the variability of the estimations. This procedure can be applied to dendrograms (see [32, 33] for examples of bootstrap applied to maize lines). We programed the R function '`hclust.boot`', which takes B samples from the genetic profiles, estimate the corresponding dendrogram for each sample and evaluate the number of times that each node (cluster) in the original dendrogram re-appears in the B samples.

[4]See the section S1-2.3 for a discussion of the estimation of allele frequencies from this kind of data.

Figure S1-2 presents the dendrogram of all 240 accessions colored by group of origin and showing the bootstrap support (in percentage) for the main groups found. For this figure we obtained $B = 1000$ samples and present the corresponding percentages of bootstrap support only for the main groups. Table S1-5 presents the statistics for the bootstrap support for all 238 clusters in the original dendrogram, P_B.

Table S1-5. Statistics for the bootstrap support. Statistics are for the percentage of times that each one of the $N_c = 238$ clusters from the original dendrogram reappered in in the $B = 1000$ bootstrap samples, say P_B.

N_c	Min.	1st Qu.	Median	Mean	3rd Qu.	Max.	S
238	0.00	3.00	13.00	21.30	35.00	96.00	22.90

From Table S1-5 we can see that the distribution of P_B goes from 0, i.e., clusters in the original dendrogram that did not reappeared in any of the $B = 1000$ re-samples (30 of them), up to a cluster with a bootstrap support $P_B = 96\%$, the maximum in Table S1-5. The median of P_B is 13, meaning that half of the 238 clusters have a bootstrap support of less than 13%, while the third quartile of the distribution is 35%, thus, only 25% of the clusters have a bootstrap support larger than this value. The clusters for which we present the P_B in Figure S1-2 (PL, PA, TE, and 'PL & PA') have a minimum $P_B = 55$ and are among the 12.6% of the clusters with higher P_B.

From Figure S1-2 we can see that the TE group forms a well segregated cluster basal to all maize accessions, with a bootstrap support of 55%. This is consistent with the ancestral role of teosintes in the domestication of maize. In the same way, all accessions from PA form a well separated cluster with bootstrap support of 59%, basal to the PL accessions, form a group with 55% bootstrap support. This, again, is consistent with the fact that Palomero is one of the ancient maize races. PL and PA accessions, the set including all maize accessions, form a cluster with 78% of bootstrap support. Even when the bootstrap procedure for dendrograms gives an idea of the support that each one of the clusters has, a more stringent and quantitative procedure to judge the robustness of the groups is given by the statistical evaluation of the differences in distances 'Between' and 'Within' groups. To test the significance of clusters, formed by the TE, PA and PL accessions, we performed an analysis of GD, separating the distances in the 'Between' and 'Within' sources by our R function `dist.bet.wit` (see S1-3.1 in the Appendix). Table S1-6 presents the results of the analysis; the 'Groups' column indicates which groups were analyzed. First three rows (PL, PA, TE), show results for the GD between each posible pair of accessions in the corresponding group; rows 'PL, PA', 'PA, TE' and 'PL, TE' present the comparisons of distances 'Between' and 'Within' the corresponding groups -i.e., in row 'PL, PA' the source 'Between' means distances between one accession of PL and other from PA, while 'Within' are for distances between the same group, PL or PA, etc. Finally, row 'PL, PA, TE' classifies the distances 'Between' and 'Within' from the full matrix of distances, using the indicator of group as '`classes`' into `dist.bet.wit`. Figure S1-3 shows box plots for the distributions of data presented in rows 1 to 3 and 13 to 15 of Table S1-6, corresponding to the groups of distances within PL, PA, TE and to the section 'PL, PA, TE' which classifies the distances 'Between' and 'Within' from the full matrix of distances.

Table S1-6 can be interpreted mainly from the values of mean distances (column 'Mean') and the interquartile ranges; main results are presented graphically in Figure S1-3. From Table S1-6 and Figure S1-3 we can see that the mean distances between accession of PL and PA, 14.65 and 14.41 respectively, are nearest to each other and separated from the mean distances for TE, 16.46. This confirms the fact presented earlier (see Table S1-2), that the TE group has a larger genetic diversity than the one detected in PL or PA. This fact is evident from the distribution of PL, PA ant TE (first three distributions in Figure S1-3); the boxes for PL and PA (first to third quartiles) overlap almost completely, while the box corresponding to TE is above these. To assess the differences between the distances at each set (PL, PA

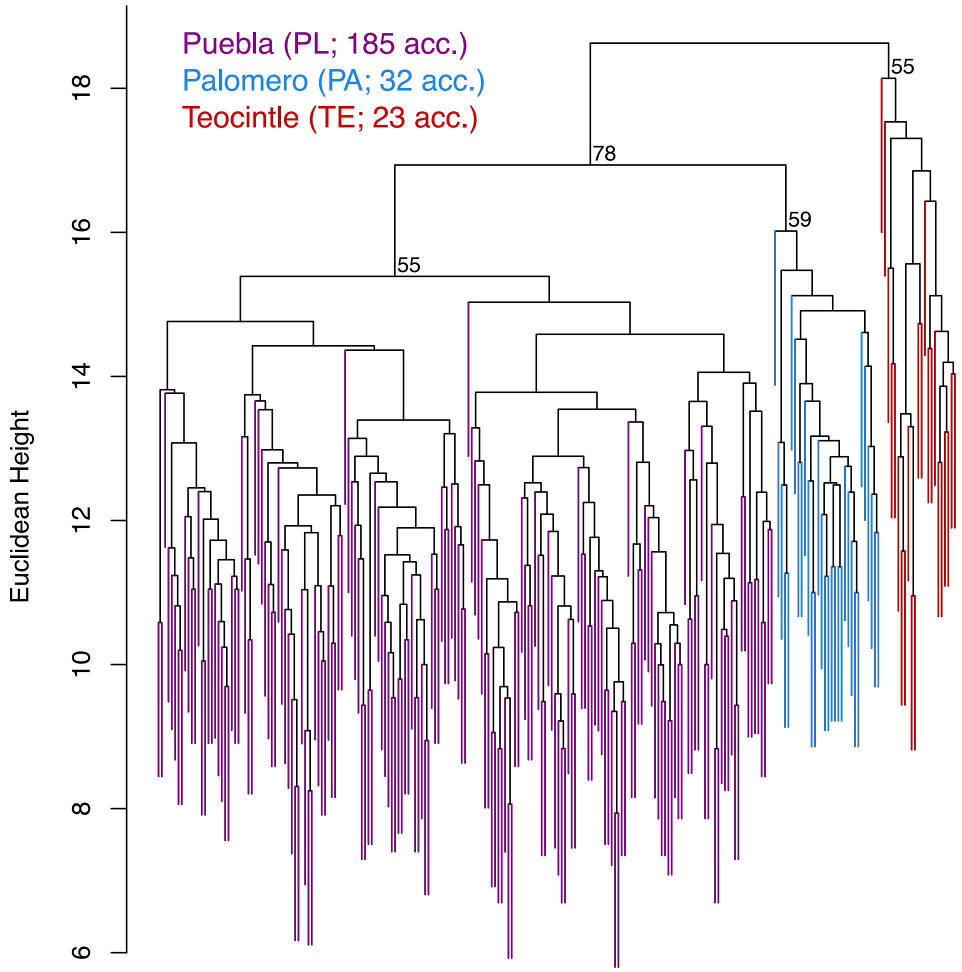

FIGURE S1-2. Dendrogram of 240 accessions colored by group of origin and obtained from the Euclidean distance of the accession's genetic profiles by UPGMA. Bootstrap support for the clusters all PL (55), PA (57), PL and PA (78) and TE (55) are shown in the corresponding nodes.

and TE), we performed the three paired comparisons by means of the Wilcoxon and t tests, and results are presented in Table S1-7 [5].

From Table S1-7 we can see that even when all three test for the means of the distances are highly significant, the largest P values, i.e., the *less* extreme, correspond to the PL *vs.* PA (2.008×10^{-5} and 4.638×10^{-5} for the Wicoxon and t-test, respectively); these values are orders of magnitude larger than the ones for the other two comparisons (PL *vs.* TE and PA *vs.* TE). With this we confirm that the accessions from TE are the ones representing a larger diversity of the three groups studied (PA, PL, TE). On the other hand, from Figure S1-3, showing the distributions of the distances 'Between' and 'Within' these three groups, as well as from the analyses in Table S1-6, we confirm that PL, PA and TE groups are well differentiated by GD.

[5]Note that results presented in Table S1-7 are testing the hypotheses of equality of the means in the corresponding vectors of distances. These are different tests than the ones performed when segregating the distances into the 'Between' and 'Within' sources, as in Table S1-6.

Table S1-6. Statistics for Euclidean distances from 240 genetic profiles segregated in the 'Between' and 'Within' sources for groups. For all 4 cases including more than one group, differences in the means 'Between' and 'Within' were highly significant; P values $< 1 \times 10^{-168}$ for Wilcoxon and t tests (not shown).

Groups	Source	n	Min.	1st Qu.	Median	Mean	3rd Qu.	Max.	S
PL	Total	17020	7.94	13.75	14.76	14.65	15.68	19.29	1.50
PA	Total	496	11.00	13.34	14.42	14.41	15.44	18.44	1.43
TE	Total	253	10.95	15.49	16.64	16.46	17.69	20.17	1.64
PL, PA	Between:	5920	12.81	16.22	16.94	16.94	17.66	20.9	1.10
	Within:	17516	7.94	13.75	14.76	14.64	15.68	19.29	1.50
	Total:	23436	7.94	14.14	15.30	15.22	16.43	20.90	1.73
PA, TE	Between:	736	15.2	17.29	18.08	18.06	18.9	21.86	1.11
	Within:	749	10.95	13.78	15.07	15.10	16.4	20.17	1.79
	Total:	1485	10.95	15.07	16.94	16.57	18.19	21.86	2.10
PL, TE	Between:	4255	14.76	17.97	18.71	18.73	19.52	22.45	1.15
	Within:	17273	7.94	13.75	14.80	14.67	15.72	20.17	1.52
	Total:	21528	7.94	14.07	15.23	15.47	16.67	22.45	2.17
PL, PA, TE	Between:	10911	12.81	16.73	17.64	17.71	18.68	22.45	1.41
	Within:	17769	7.94	13.75	14.76	14.66	15.72	20.17	1.52
	Total:	28680	7.94	14.42	15.75	15.82	17.26	22.45	2.09

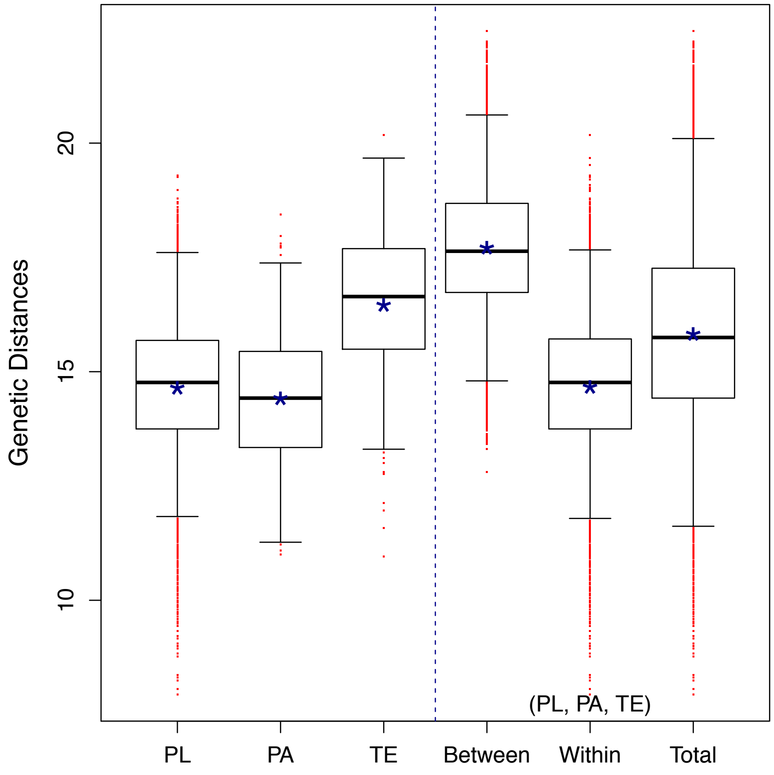

Figure S1-3. Distributions (as box plots) of Euclidean GD in groups of PL, PA, TE (rows 1 to 3 in Table S1-6) and by segregating the full matrix of distances in their 'Between' and 'Within' components (last rows in Table S1-6). Medians are shown as broad lines, while means are marked by asterisks. Bottom and top of the boxes are from the first to the third quartiles. Boxplot whiskers extend 1 interquartile range unit from the boxes. Outliers are shown as red points.

Table S1-7. Results for Wilcoxon and t tests comparing the mean distances between groups

Comparison of distances	Wilcoxon W	Wilcoxon $P[W]$	t-test t-value	t-test $P[t]$
PL *versus* PA	4694300	2.008×10^{-5}	3.523	4.638×10^{-5}
PL *versus* TE	865960	4.487×10^{-60}	17.517	8.095×10^{-46}
PA *versus* TE	21738	1.503×10^{-48}	16.843	9.130×10^{-50}

S1-2.3. **Estimation of allele frequencies from bulks.** As mentioned before, by sampling bulks of 10 plants, when we detect the presence of a given allele, this can be translated as '*at least one allele was present*' –corresponding a value of 1 in the marker/allele profile of an individual bulk. The genetic profiles of individual bulks are added to represent the genetic profile of an accession. In this case the variable corresponding to each marker/allele can take values 0, 1, 2 or 3. From this kind of data no direct estimate of allele frequency is obtained. Here we briefly study this problem.

Let's X be the random variable denoting the number of *gametes* (haplotypes) which present the allele 'a' in a population. Assume that a random sample of s alleles is taken ($s/2$ individuals), then X has a binomial distribution of parameters p (*success* probability; the frequency of the allele 'a', our parameter of interest) and s (number of assays) or, in symbols,

$$X \backsim \mathfrak{B}(s, p)$$

thus

$$P[X = x] = \frac{s!}{x!(s-x)!} p^x (1-p)^{s-x}$$

Now, consider the process of taking a bulk of s alleles and assume that we can observe only a variable Y which will take only two values, say $Y = 0$ (a *negative* result if $X = 0$) and $Y = 1$ if $X > 0$. This variable is what we observe in a bulk; i.e., a 'positive' bulk ($Y = 1$) or a negative one ($Y = 0$) for allele 'a'. It follows that

$$P[Y = 0] = (1-p)^s \text{ and } P[Y = 1] = 1 - (1-p)^s$$

Assume that we have k independent realizations of Y, say $Y_1, Y_2, \cdots, Y_k$, which correspond to independent bulks of individuals. Now consider Z as the sum of Y from $i = 1$ to $i = k$. Z is, again, a random variable with binomial distribution of parameters $q = 1 - (1-p)^s$ (success probability) and k (number of assays), say

$$Z \backsim \mathfrak{B}(k, 1 - (1-p)^s)$$

The maximum likelihood estimator (MLE) of q –which is not directly our parameter of interest, is given by

$$\hat{q} = 1 - \widehat{(1-p)^s} = z/k$$

Given the invariability of the MLE procedure under one to one transformations we have that the MLE of p is given by

$$\hat{p} = 1 - (1 - z/k)^{1/s}.$$

In our particular case of interest we have $s = 20$ (we are sampling bulks of 10 individuals) and $k = 3$ (three independent bulks are sampled), thus

$$\hat{p} = 1 - (1 - z/3)^{1/20}.$$

Table S1-8 presents the numerical value of the estimations of p, $\hat{p}$, as function of the number of positive bulks. The practical implications of the data presented in Table S1-8 are that the genetic scale (estimated frequency of the allele, $\hat{p}$) is not lineal with the scale in z that we used to estimate GD. In fact, a zero or a three in the number of positive bulks are the extremes of the parameter's space, 0 and 1 respectively, but when we see just one positive bulk in three we estimate (by maximum likelihood) a frequency of around one in fifty alleles ($1/50 \approx 0.02$) and when we see two positive bulks we estimate a frequency of around $1/19 \approx 0.05$. We empirically tested the measurement of GD from MLE, by changing the scale of

Table S1-8. Values for the score z in the genetic profile of accessions and the corresponding maximum likelihood (ML) estimates for the frequency of the allele 'a' in the case of $k = 3$ bulks of $s/2 = 10$ plants.

z	$\hat{p}$	$\approx \hat{p}$
0	0.00000000	0
1	0.02006913	1/50
2	0.05344918	1/19
3	1.00000000	1

measurement in the marker/allele vectors from z to $\hat{p}$ using the values in Table S1-8. From the matrix of $\hat{p}$ values we obtained the estimated Euclidean GD and constructed the UPGMA dendrogram presented in Figure S1-4.

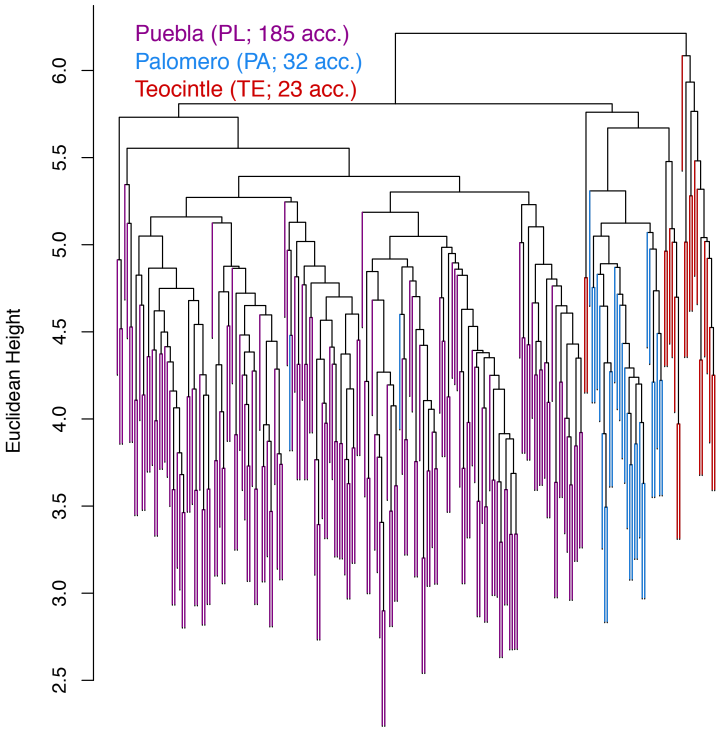

Figure S1-4. Dendrogram of 240 accessions colored by group of origin and obtained from the Euclidean distance of the accession's genetic profiles expressed as MLE of the marker/allele frequencies ($\hat{p}$; see Table S1-8) by UPGMA.

From Figure S1-4 we can see that the dendrogram shows clusters that mix accessions from different groups (colors in Figure S1-4); the cluster formed mainly by accession from PL (in violet), includes two accessions from PA (in blue) and the cluster formed mainly from PA maize accessions (in blue) includes 9 accessions from TE (in red) –a different specie. In contrast, the dendrogram presented in Figure S1-2, constructed with the z scale, recovers the expected and logical topology for the PL, PA, TE groups. From this result, as well as from the fact that the distribution of the distances 'Between' and 'Within' groups estimated from the matrix in the $\hat{p}$ scale are less well separated than in the case of the z scale (data not shown), we conclude that GD estimated from ML is distorted. As we will see this is explained by the fact that in this case the MLE is highly biassed.

The natural parameter of interest in this case is the frequency of the allele in the population, p. However, the number of alleles in a sample, X, is not observed and we obtain information about its value only trough Y and their sum, Z, the number of positive bulks. In our case ($s = 20$ alleles per bulk and $k = 3$ bulks), we can observe only the values of Z which are the natural numbers 0 to 3. We will say that $\hat{p}$, the MLE of p, will be unbiased if $E[\hat{p}] = p$ for all $0 \leq p \leq 1$. By definition we have

$$E[\hat{p}] = \sum_{z=0}^{z=k} \hat{p}P[Z = z|p] = \sum_{z=0}^{z=k} (1 - (1 - z/k)^{1/s})P[Z = z|p]$$

where $P[Z = z|p]$ is the binomial probability with success probability $1 - (1 - p)^s$. Figure S1-5 presents the numerical evaluation for $E[\hat{p}]$ in our case ($s = 20$ alleles per bulk and $k = 3$ bulks) for all p range.

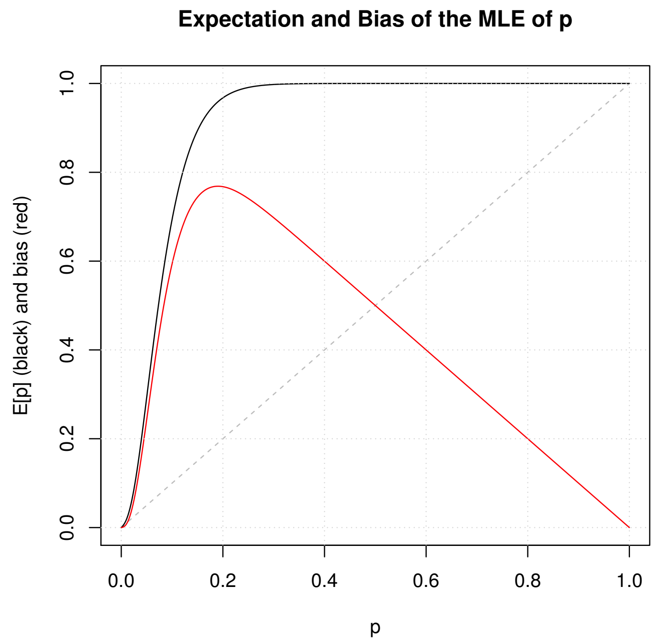

FIGURE S1-5. Expected value for the MLE of p ($E[\hat{p}]$) as function of the frequency of the allele p (black curve). The red line presents the bias ($E[\hat{p}] - p$) of the estimator while the grey dotted line illustrates the case of an hypothetical unbiased estimator.

From Figure S1-5 we can see that the MLE of p is biased 'almost everywhere', except in the extremes of the parameter space (values of $p = 0$ and $p = 1$). The spectacular bias of $\hat{p}$ reflects the loss of information caused by bulking and make ML estimation useless for this case. Estimators with less bias and standard error than the ML estimator $\hat{p}$ can be constructed by *assuming* a lineal function between the values of z and p (results not shown), however for simplicity and clarity, the natural scale z appears satisfactory.

S1-2.4. **Definition and estimation of *rareness* coefficient (R_i or ρ).** For *ex-situ* conservation – where all accessions are currently grown, it appears reasonable to give more importance, or priority, to unusual or 'rare' accessions. From the genetic point of view it can be argued that an accession is rare if it has a mesure of 'frequency' that is far from the mean 'frequency' in the population. As we have seen in section S1-2.3, our scale for marker/allele combinations in the accession's genetic profiles, z, with possible values 0, 1, 2 or 3, is biased for genetic frequencies, but gives a satisfactory measure of genetic distance. We defined a coefficient of rareness, which gives square weight to the difference between the observed and mean marker/allele values. Let's z_{ij} be the value observed in the $i-th$ accession, $i = 1, 2, \cdots, a$,

and the $j-th$ marker/allele combination, $j = 1, 2, \cdots, k$; where k is the total number of marker/allele combinations. Defining the mean value of the $j-th$ marker/allele combination as

$$\bar{z}_j = \frac{1}{a}\sum_{i=1}^{a} z_{ij}$$

we now define the rareness coefficient, ρ, for each accession i as

$$\rho_i = \sqrt{\frac{1}{k}\sum_{j=1}^{k}(z_{ij} - \bar{z}_j)^2}$$

i.e., ρ_i [6] is the square root of the average squared differences between the values of the accession's genetic profile with the corresponding means. By normalizing with the $1/k$ factor and extracting the square root, the scale of ρ_i is set in the same scale than the original scale of measure of our $z = 0, 1, 2, 3$ units, making the interpretations of results more intuitive.

Table S1-9 and Figure S1-6 present statistics and distributions of ρ in the context of all 240 accessions.

TABLE S1-9. Statistics for the rareness coefficient ρ estimated in the context of all (240) accessions, and presented for each one of the groups (PL, PA, TE) as well as for the complete set (All).

Group	n	Min.	1st.Qu.	Median	Mean	3rd.Qu.	Max.	S
PL	185	0.5284	0.6032	0.6290	0.6317	0.6575	0.7671	0.0430
PA	32	0.5976	0.7175	0.7441	0.7417	0.7754	0.8554	0.0516
TE	23	0.7981	0.8305	0.8771	0.8742	0.9002	0.9798	0.0531
All (a)	240	0.5284	0.6109	0.6453	0.6696	0.7019	0.9798	0.0887

From Table S1-9 we can see that the estimated values for ρ goes from a minimum of 0.5284 up to a maximum of 0.9798; roughly the rareness goes from 0.5 up to 1 units in the original scale of the data. This means that there are 'very common' accessions which in average differ from the mean marker/allele 'frequency' in approximately 1/2 of z units. The five more common accessions are PL048, PL134, PL168, PL085 and PL017 with ρ values 0.5284, 0.5378, 0.5447, 0.5498 and 0.5549, respectively. The five most rare accessions are TE023, TE064, TE004, TE063, TE017 and TE062 with ρ values 0.9798, 0.9665, 0.9599, 0.9339, 0.9078 and 0.9049, respectively. As expected the group with the rarest accessions is formed by TE, while the most common (least rare) are the accessions from the PL group (Figure S1-6). Apart from the sample sizes of the groups ($n = 185, 32, 23$ for PL, PA and TE respectively), the average values of rareness, and in general the location of the distributions are influenced by the fact that the three groups are genetically distant, as seen before. To appreciate the rareness of the accessions in the context of the dendrogram, we colored the accessions by their value of ρ. The result is presented in Figure S1-7.

From Figure S1-7 we can see that all accessions classified as '*Very common*' ($\rho \leq 0.62$) are included into the PL cluster, i.e., all of them are from the maize Puebla set. On the other hand, all accessions from the TE cluster are classified as '*Very rare*' ($\rho > 0.72$). Those two facts are expected from the sample size and origin of the accessions, however it is interesting to note that the PL cluster also includes '*Rare*' ($0.66 < \rho \leq 0.72$) and '*Very rare*' ($\rho > 0.72$) accessions, while the PA cluster includes one '*Very common*' ($\rho \leq 0.62$) and one '*Common*' ($62 < \rho \leq 0.63$) accession. This means that the rareness coefficient, ρ, is capturing an aspect of genetic diversity that is not explained by the grouping of the accession into clusters. This implies that for conservation proposes is not enough to select accessions from '*representative*' clusters,

[6] This coefficient is denoted as R_i in the main text.

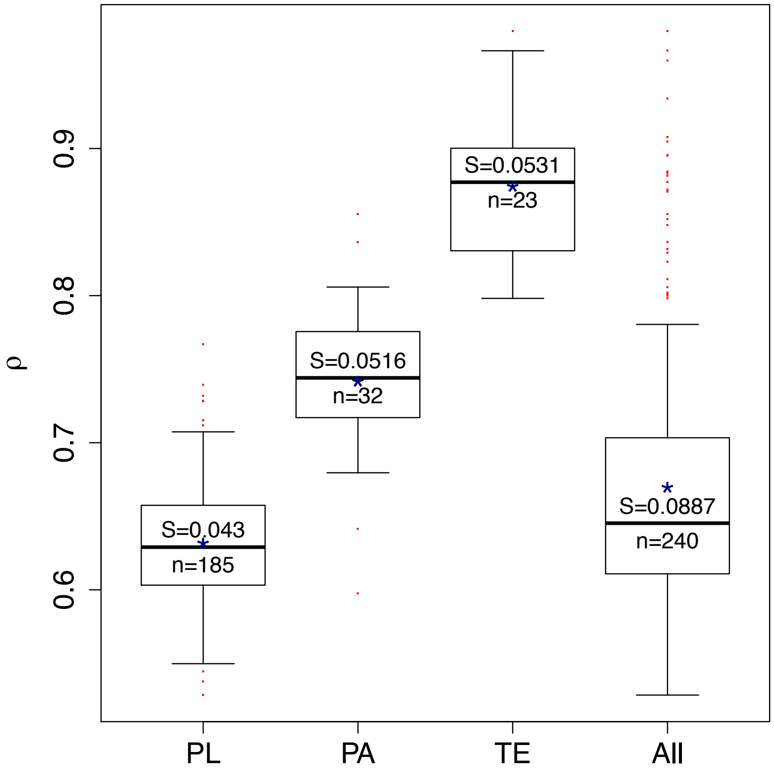

FIGURE S1-6. Distributions (as box plots) of the rareness coefficient ρ estimated in the context of all ($a = 240$) accessions for $k = 278$ marker/allele combinations, and presented for each one of the groups (PL, PA, TE) as well as for the complete set (All). Bottom and top of the boxes are from the first to the third quartiles. Boxplot whiskers extend 1 interquartile range unit from the boxes. Outliers are shown as red points. Number of observations (n) and standard deviations (S) are shown within the corresponding boxes. Means shown as asterisks.

for example, by taking one accession from each cluster above a given height; such a procedure could fail to select a representative set of accessions –a problem that we board in the next section.

S1-2.5. **Algorithm '*All Marker Alleles*' (AMA) to capture all marker/alleles in a small set of accessions.** A common concept for preserving genetic diversity from *ex-situ* germplasm collections stored into gene banks, is to select a '*core collection*' [12, 3]. A core collection is a subset of the full accession's set, that maximize one or more criteria with the general aim of preserving a high proportion of the genetic diversity present in the full collection in a 'small' sub-set of accessions. In many cases, the size of the core collection is fixed in advance, as a proportion of the size of the full collection or simply as the number of accessions desired. There are various algorithms to obtain core collections; among the more recently reported are 'MSTRAT' [15], 'PowerCore' [19] and 'Core Hunter' [30]. In particular, 'Core Hunter' implements an stochastic local search algorithm for selecting core subsets; it can attempt to optimize any number of genetic measures simultaneously, for example the algorithm is capable of selecting core subsets that have high average genetic distance between accessions, or rich genetic diversity overall, or a combination of both, and in many cases it outperforms other algoritms [30].

Our aim here was to develop a fast an efficient algorithm to select a small set of *in situ* accessions that will include all marker/allele combinations present in the full collection. As a secondary objective the algorithm will prioritize '*rarer*' accessions over common ones. When facing resources limitations for

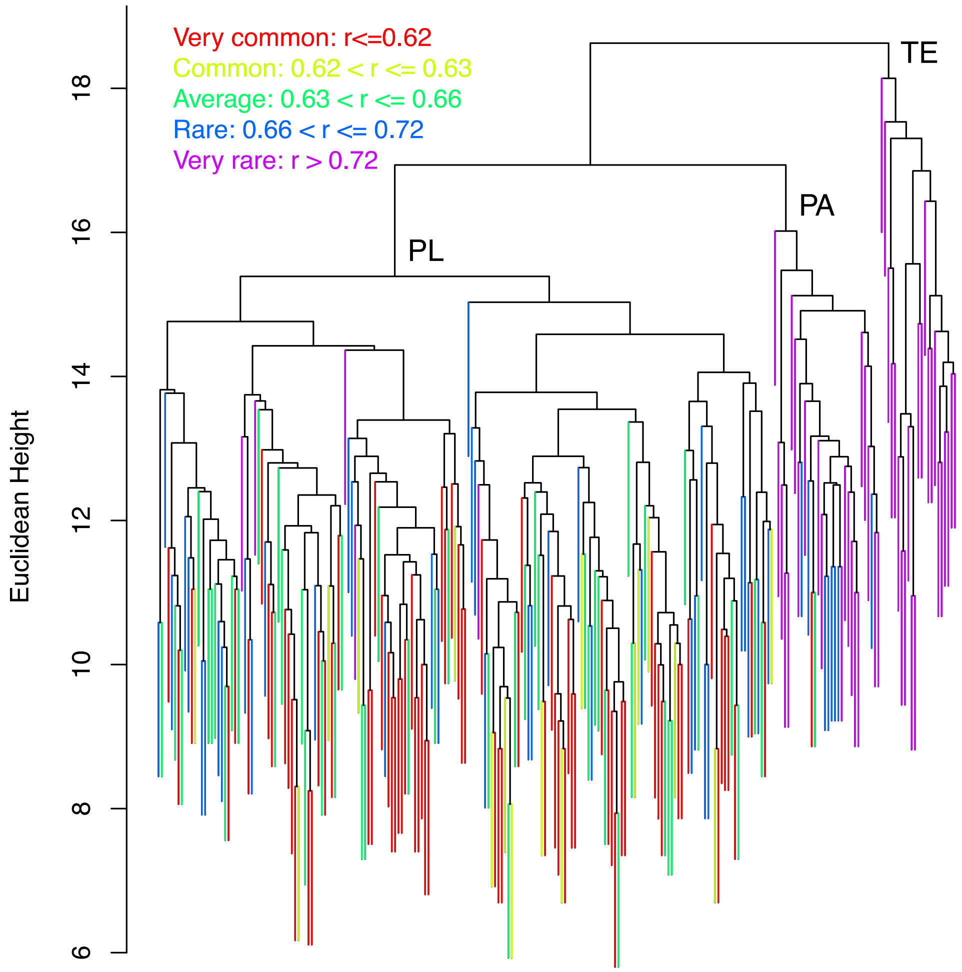

FIGURE S1-7. Dendrogram of 240 accessions colored by 'rareness' coefficient (ρ; shown as 'r' in the legend). Clusters for PL, PA and TE groups with 185, 32 and 23 accessions respectively are identified in the corresponding nodes; see also Figure S1-2 for coloring by group.

the *in situ* conservation of the full set of accessions, the knowledge of the set of accessions that comprises all marker/allele combinations and keeps the rarest ones will give a sensible criteria for resource's allocation.

For any set of accessions, $\mathcal{A}$, we denote as $\#(\mathcal{A})$ the cardinality function, i.e., the number of elements of the set $\mathcal{A}$. Also denote as $M(\mathcal{A})$ the function that counts all distinct marker/allele combinations in the set of accessions $\mathcal{A}$.

Let's $\mathcal{C}$ be the *complete* (full) set of accessions in a given collection, and assume that $\#(\mathcal{C}) = t$ and $M(\mathcal{C}) = n$, thus in the complete set we have t accessions and n marker/allele combinations. Also define the class $\mathbb{C}$ as all not empty subsets of $\mathcal{C}$, say

$$\mathbb{C} = \{\mathcal{A} | \{\mathcal{A} \subset \mathcal{C}\} \cap \{\#(\mathcal{A}) > 0\}\}$$

and the class of all collections that include all t marker/allele combinations, say the sets $\mathcal{T} \in \mathbb{C}$ that fulfill

$$\mathbb{T} = \{\mathcal{T} | M(\mathcal{T}) = n\}$$

thus we can define the class of optimum sets as the sets $\mathcal{O} \in \mathbb{T}$ that fulfill

$$\mathbb{O} = \{\mathcal{O} | \#(\mathcal{O}) \leq \#(\mathcal{T}), \mathcal{T} \in \mathbb{T}\}$$

In short, a set $\mathcal{O}$ is 'optimum' if the accessions that form it have all n marker/allele combinations present in the full collection and the number of accessions of such set, $\#(\mathcal{O})$, is the smallest possible.

We present an algorithm to obtain an optimum set of accessions, $\mathcal{G}$, which has a number of accessions close to the minimum possible, $\#(\mathcal{G}) \approx \#(\mathcal{O})$, and also has a rareness coefficient, ρ, larger than the optimum sets, $\mathcal{O} \in \mathbb{O}$.

The algorithm –presented below in S1-2.5.1, begins from a matrix $\mathbf{z}$ of order $t \times n$ (accessions $\times$ marker/allele combinations), where each element, z_{ij} is a quantitative measure of the $j-th$ marker/allele combination in the $i-th$ accession. $\mathbf{z}$ can be a matrix of estimated genetic frequencies for the marker/alleles in the accessions, or, as in the case of our measure in the plant bulks, simply a quantification of the presence of the marker/allele; remember that for our data we do not estimate allelic frequencies, but quantify by $z = 0, 1, 2$ or 3, the presence of the marker/allele in plant's bulks. The only requirements for the z measure is that it will give reasonable results for the rareness coefficient ρ defined before, and that a value of $z = 0$ implies the non-detection of the corresponding marker/allele combination. Obviously each value of z must be non-negative ($z \geq 0$).

S1-2.5.1. *The AMA algorithm.* Begins with a matrix $\mathbf{z}$ of measures for the presence of each marker/allele (column) at each accession (row). It can contain marker/allele frequencies or other measure.

(1) From $\mathbf{z}$ (of order $t \times n$) obtain the vector of estimated *rareness* coefficients, $\rho = (\rho_1, \rho_2, \cdots, \rho_t)$; see section S1-2.4 for the definition of ρ.

(2) From $\mathbf{z}$ obtain a binary matrix of presence / absence, say $\mathbf{m}$, defined by $m_{ij} = I(z_{ij})$ where the indicator function $I_{\{x>0\}}()$ is defined as $I_{\{x>0\}}(x) = 1$ if $x > 0$ and $I_{\{x>0\}}(x) = 0$ if $x = 0$.

(3) Define an empty set $\mathcal{G}$ (will contain selected accessions).

(4) Obtain a vector $\mathbf{c} = (c_1, c_2, \cdots, c_n)$ in which $c_i = 1$ for the marker/alleles present in the accessions with unique marker/alleles and $c_i = 0$ otherwise.

(5) Obtain a list with all accessions (rows of $\mathbf{m}$) which have unique marker/allele combinations, i.e., marker/allele combinations that are present in a single accession, and if such a list is not empty, set $\mathcal{G}$ equal to the names (or identifiers) of all those accessions.

(6) Update the vector $\mathbf{c}$ setting to 1 the values corresponding to all the marker/alleles that are present in $\mathcal{G}$.

(7) **IF** $M(\mathcal{G}) = n$, equivalently if $\sum_j c_j = n$, then **GO** to (14), **ELSE** continue with (8).

(8) For each accession (row) in $\mathbf{m}$ calculate the gain in marker/alleles, $g_i = \sum_j m_{ij} - c_j$.

(9) Select the row, or rows, say r, which gives the maximum gain, $\max_i(g_i) = g_r$.

(10) **IF** $\max_i(g_i) = 0$ **GO** to (13), **ELSE** continue with (11)

(11) **IF** there are ties in the maximum of the gains, i.e., if more than one value r gives the same $\max_i(g_i) = g_{r1} = g_{r2} = \cdots = g_{rk}$ for different values of r, then select the accession (row) with largest rareness coefficient, say an accession that fulfills that $\rho_r = \max\{\rho_u\}$ where u is the subscript for the tied accessions. If ties persist (because two or more ρ coefficients are equal to the maximum), then, select the first accession that has a maximum value of ρ.

(12) Include the selected accession in the set $\mathcal{G}$

(13) Update the vector $\mathbf{c}$ including 1 in the places corresponding to the new marker/allele combinations found in the accession selected in (11) and **GO** to (7).

(14) **OUTPUT** the set $\mathcal{G}$ and **STOP**.

R code implementing the AMA algorithm in a function called `AMA` is shown in the appendix under section S1-3.2. The output from `AMA` includes, apart from the set $\mathcal{G}$, extra output to help in the evaluation of the algorithm and interpretation of results.

For this discussion, numbers between parenthesis refer to the steps of the algorithm (S1-2.5.1 above).

In contrast with many of the algorithms to obtain core collections, AMA is completely deterministic, i.e., not random or stochastic factors are introduced in the selection of the accessions to be included into $\mathcal{G}$. The algorithm assures that the selected collection will include all marker/alleles, i.e., it guarantees the complete coverage, $M(\mathcal{G}) = n$. When complete coverage is required, *any* algorithm must include the accessions that present a unique marker/allele in the full collection. This is accomplished in steps (5) and (6), and the set of accessions that contain unique marker/alleles is named as '`acc.unique`' in the output of `AMA`. If the accessions in `acc.unique` also contain all other marker/alleles combinations (step (7)) the algorithm stops and outputs the results. If that is not the case –as it will usually happen, then more accessions must be added to achieve complete coverage. To add further accessions AMA selects, in successive steps (loop from (8) to (13)), the accession that adds more new marker/alleles to the selected collection. The gain in marker/alleles is evaluated in (8) for *all* accessions. This is redundant, because accessions already in $\mathcal{G}$ must give a gain of zero. By measuring the gain exclusively in number of new marker/alleles that will be added to the current $\mathcal{G}$, we can state with confidence that the algorithm will give a number of accessions close to the the number of accessions in the members of the optimum sets, $\#(\mathcal{O})$; $\mathcal{O} \in \mathbb{O}$. This is so because, in absence of ties, to include the accession which contributes with more new marker/allele (maximum gain) we are minimizing the number of accessions that will finally be part of $\mathcal{G}$. When ties are present, i.e., when two or more accessions will give the same gain, we employ as criterion the *rareness*, this is, the value of ρ, selecting as the accession that will enter into $\mathcal{G}$ the 'rarest' one, which has the largest value of ρ within the accessions tied by gain. This also assures that the current set $\mathcal{G}$ will grow including marker/alleles that are in a few accessions and help in achieving the secondary objective of including the rarest accessions into the selected set. Thus, in general, the algorithm will increase the number of accessions beginning with the most rare marker/alleles and moving towards more common marker/alleles. However, because when selecting one accession to form part of $\mathcal{G}$ we select *all* marker/alleles present in such accession, the most common marker/alleles will very likely be included in the first steps of the algorithm, and in no case the selection of a single accession will be needed to include such marker/alleles into the ones present in $\mathcal{G}$. The only case where the algorithm randomize the selection of an accession is when there are ties in both, the genetic gain and rareness (ρ) of the accessions. In such case, the algorithm arbitrarily select the 'first' accession (an arbitrary order and thus 'at random'). Note that if the non-selected accessions (from the ones tie in gain and ρ) are still relevant in further steps, by having positive gains, they will be selected in the order of their value of ρ.

It is important to notice that AMA uses the frequencies of the marker/allele combination –if present, or in general, the measure $\mathbf{z}$, only in the estimation of ρ to solve ties on gains. All other operations are performed over the binary matrix $\mathbf{m}$, simplifying the search for $\mathcal{G}$. As a result the algorithm is very fast, requiring an absolute maximum of $t(t+1)$ evaluations of the main loop.

We applied AMA –implemented in the function `AMA`, to different initial sets of accessions, formed by accessions of groups PL, PA and TE. Results for the seven possible sets are presented in rows of Table S1-10.

Table S1-10 present the characteristics of the original set input to the algorithm; the number of original accessions, t, and number of different marker/allele combinations, n, as well as the characteristics of the resulting minimum set of accessions, $\mathcal{G}$, for which we present the number of accession that constitute it, $\#(\mathcal{G})$, as well as the numbers of the origin of the accessions, #PL, #PA and #TE. The last columns of Table S1-10 present the number of steps performed –column 'Steps', number of times that ties were found and solved by the ρ coefficient –column 'Ties', as well as the number of accessions that were originally selected (step (5) in S1-2.5.1) by containing unique marker/allele combinations –column 'Ini. Acc.'. From

Table S1-10. Results obtained with AMA for different sets of accessions.

Initial set			Result; $\mathcal{G}$				Number of		
Group	t	n	#($\mathcal{G}$)	#PL	#PA	#TE	Steps	Ties	Ini. Acc.
PL∪PA∪TE	240	278	40 **(16.67%)**	21	11	8	19	15	22
PL∪PA	217	257	36 **(16.59%)**	24	12	–	17	13	20
PL∪TE	208	261	32 **(15.38%)**	22	–	10	16	11	17
PA∪TE	55	238	29 **(52.73%)**	–	16	13	6	4	24
PL	185	236	30 **(16.22%)**	30	–	–	14	12	17
PA	32	197	21 **(65.62%)**	–	21	–	3	0	19
TE	23	196	17 **(73.91%)**	–	–	17	2	1	16

Table S1-10 we can notice that the reduction in percentage of the number of accessions from the original set to the selected set goes from a minimum of 15.38% for the PL∪TE group, constituted originally by 208 (185 from PL and 23 from TE), up to a maximum of 73.91% for the group formed by 23 TE accessions, of which 17 are needed to keep all 196 distinct marker/allele combinations. In the group of only maize (PL∪PA), with 217 accessions and 257 marker/allele combinations, the results show that a set of 36 accessions (16.59% of the total) can keep the set of all 257 marker/allele combinations. The reduction from the original to the selected set depends in a complicated way on the diversity of the full collection; the accessions which present unique marker/alleles are selected first –the number of these is presented in column 'Ini. Acc.'. From there, the algorithm begin to add accessions, and the number of steps that it take to fulfill the objective is in column 'Steps'. In all cases presented in Table S1-10 this number is small –from 16 to 22, far away from the theoretical maximum, $t(t+1)$. On the other hand, the number of ties in gain found by the algorithm go from 0 –for the case of the PA group up to 15 for the PL∪PA∪TE group. In that case the number of steps was 19 and in 15 of these ties were found and solved by the value of ρ; this underlines the importance of the use of the rareness criterium. By examining the 15 cases of ties in the output of the AMA for the group PL∪PA∪TE we found that the number of accessions with equal gain at some step in the algorithm goes from 2 up to 23 with a median of 5. In all these 15 cases it was possible to 'un-tie' the accessions by selecting the rarest (the one with maximum ρ).

Figure S1-8 shows the dendrogram previously obtained by UPGMA from the Euclidean distance of all 240 accessions (as in figures S1-2 and S1-7), but coloring by origin the accessions selected by AMA (see Group PL∪PA∪TE in Table S1-10).

In Figure S1-8 we can see that the 40 selected accessions are scattered into the three major groups formed by PL, PA and TE. However, the intensity of selection was not the same into the three groups; we have 185, 32 and 23 accessions from the PL, PA and TE and in the selected set we have 21 from PL, 11 from PA and 8 from TE, corresponding to 11.35% of the original PL, 34.38% of the original PA and 34.78% of the original TE accessions; larger proportions were selected from the more diverse groups, PA and TE, which are also the ones with smaller original sample.

Figure S1-9 presents the dendrogram constructed by UPGMA of the Euclidean distance for the accessions selected by the AMA algorithm for all groups (see 1st row, group PL∪PA∪TE in Table S1-10). From this figure we can see that the topology of the dendrogram cluster the accessions per group, as in Figure S1-8, with a clear segregation between them. Comparing the places where the inter-group nodes are formed (Euclidean height or distance, X-axis) between figures S1-8 (all accession) and S1-9 (selected accession), we can notice that in general de inter-group nodes are at higher distance in Figure S1-9 than in Figure S1-8; in the first the scale goes from 10 to 18 while in the second it goes from 6 to 18. This indicates that the selection algorithm is, apart from capturing all marker alleles in the collection, performing a maximization of the distance between accessions. This unintended –but desirable side effect, is a result of using the values of ρ in the algorithm to un-tie the gain ties between accessions; by using the rareness coefficient we increase the diversity between selected accessions.

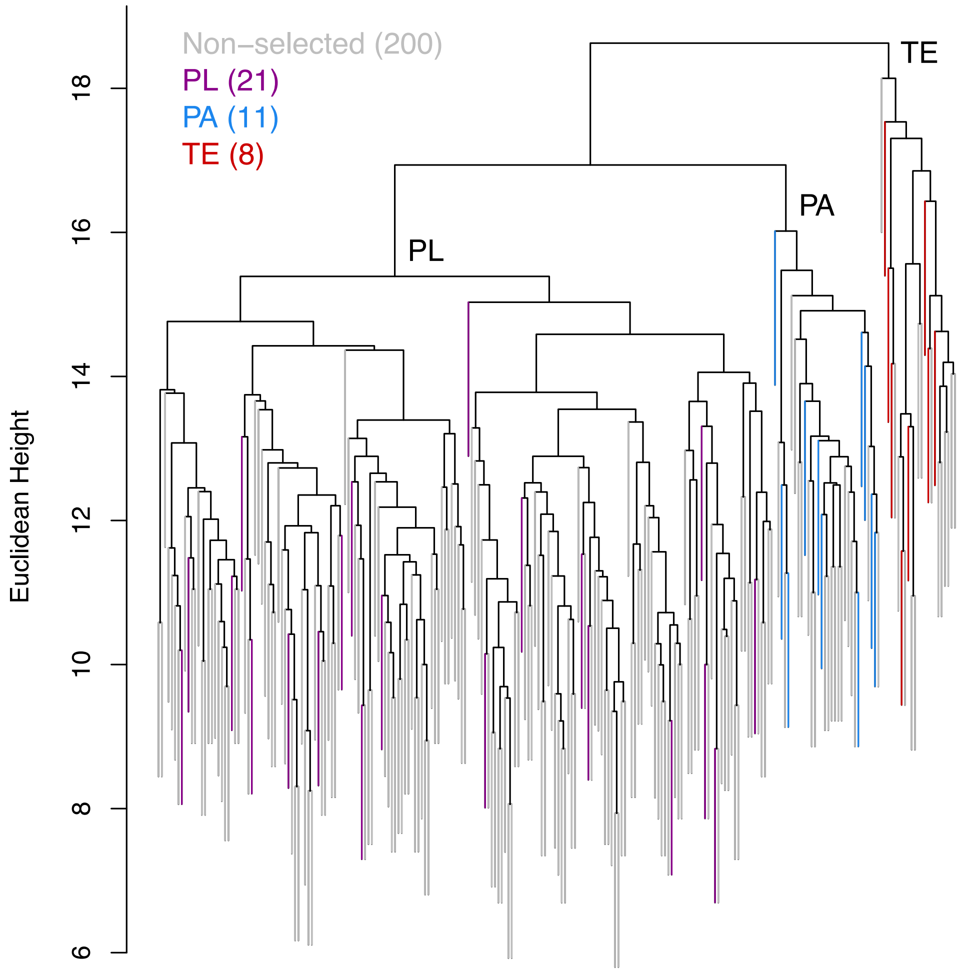

FIGURE S1-8. Dendrogram of 240 accessions colored in gray if they were not selected by `AMA` to form the group $\mathcal{G}$, and colored by group of origin when they were selected. Obtained from the Euclidean distance of the accession's genetic profiles by UPGMA as in figures S1-2 and S1-7.

To see how the distances between and within accessions compare in the unselected (all accessions) and selected (group $\mathcal{G}$ obtained with `AMA`), we performed an analysis with the function `dist.bet.wit` which results are presented in Table S1-11

TABLE S1-11. Statistics for Euclidean distances from 240 (non-selected) and 40 (selected) genetic profiles segregated in the 'Between' and 'Within' sources for groups PL, PA and TE. Results for 'Non-selected' had been presented before in Table S1-6.

Groups	Source	n	Min.	1st Qu.	Median	Mean	3rd Qu.	Max.	S
	Between:	10911	12.81	16.73	17.64	17.71	18.68	22.45	1.41
Non-selected	Within:	17769	7.94	13.75	14.76	14.66	15.72	20.17	1.52
	Total:	28680	7.94	14.42	15.75	15.82	17.26	22.45	2.09
	Between:	487	14.56	17.13	18.06	18.11	19.09	21.35	1.32
Selected	Within:	293	11.79	14.39	15.46	15.41	16.40	19.52	1.46
	Total:	780	11.79	15.87	17.19	17.10	18.52	21.35	1.90

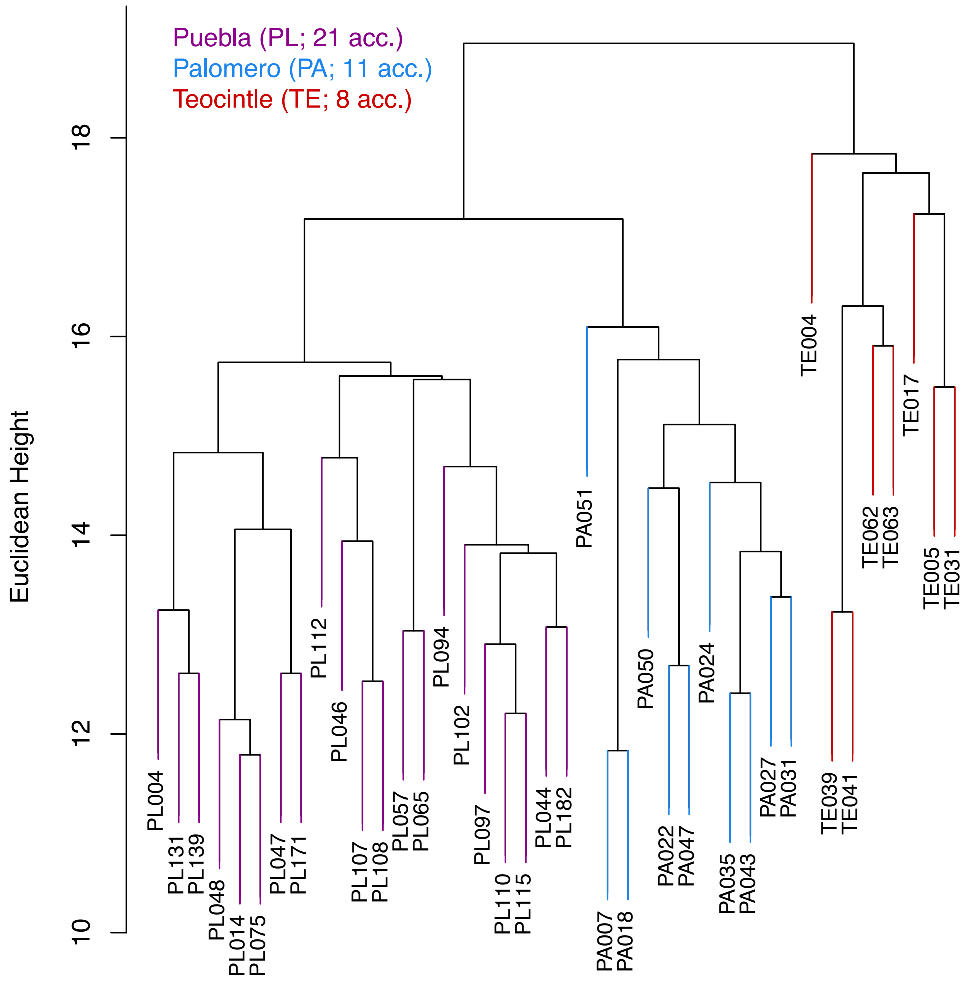

Figure S1-9. Dendrogram of 40 accessions selected by `AMA` to form the group $\mathcal{G}$ (first row of Table S1-10), and colored by group of origin. Obtained from the Euclidean distance for the selected accession's genetic profiles by UPGMA.

As for the analysis of 'Between' and 'Within' distances for the un-selected accession, the corresponding analysis in the selected group is highly significant ($P \approx 9.4 \times 10^{-85}$ in the Wilcoxon test), corroborating that the selected accessions have a much lower average distance within groups PL, PA and TE that between them. In Table S1-11 we observe that for the selected accessions, the mean distance 'Within' groups is 15.41 while the mean distance 'Between' groups is 18.11, in both cases with a standard deviation of approximately 1.4. From Table S1-11 we can also see the differences in the 'Total' distances in the 'Non-selected' (row 3) and 'Selected' (row 6) groups. Figure S1-10 shows the distributions of the distances numerically summarized in Table S1-11.

From Figure S1-10 we can see that, even when the ranges of the distances in the selected accessions are smaller than the corresponding ones in the not selected accessions, the mean and medians are approximately equal in the corresponding 'Between' and 'Within' groups; this means that the selection algorithm keeps (and even increase a little) the observed distances, fairly representing the diversity of the full collection in the selected one. In fact, the means of the distances in the 'Total' group (including all accessions without segregating the sources) is larger in the 'Selected' (mean equal to 17.1) than in the 'Not-selected' (mean 15.8) groups, and this difference is highly significant in both, the Wilcoxon and the

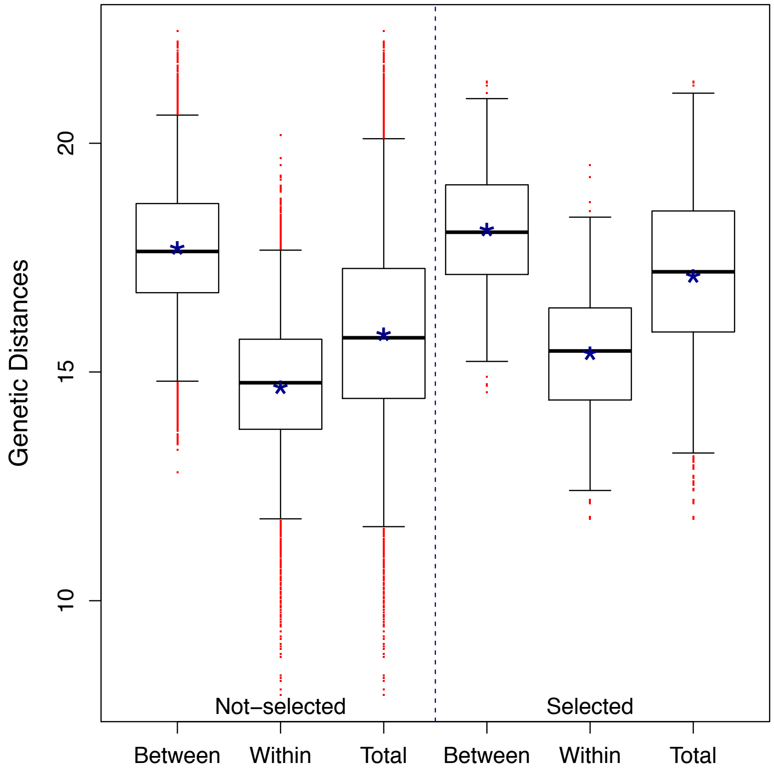

FIGURE S1-10. Distributions (as box plots) of Euclidean GD in Not-selected and Selected groups of accessions segregated into 'Between' and 'Within' sources ('Total' are also plot; see numeric data in Table S1-11). Medians are shown as broad lines, while means are marked by asterisks. Bottom and top of the boxes are from the first to the third quartiles. Boxplot whiskers extend 1 interquartile range unit from the boxes. Outliers are shown as red points.

t tests (P values smaller than 1×10^{-16} in both cases). This means that the selection algorithm not only keeps, but increases the distances between accessions from the full to the selected groups.

S1-2.5.2. *Comparing 'AMA' with 'Core Hunter II'.* 'Core Hunter II' [5] is the state of the art program for subset selection in germplasm *in situ* collections. It employs the 'Mixed Replica' search algorithm (MixRep) and is able to optimize in parallel multiple genetic measures, including both distance measures and allelic diversity indices. To compare AMA with Core Hunter we evaluated our own data (the set of 240 accession from PA, PL and TE) and a set of 275 accessions, genotyped at 24 SSR loci with 186 total alleles [6], that had been used when comparing 'Core Hunter' [30] and 'Core Hunter II' [5] with other algorithms.

Core Hunter (version 2.0, obtained from www.corehunter.org) uses the parameter '`-sample_intensity`' to determine the size of the selected group (as a proportion of the size of the full collection), and '`-CV <weight>`' to select the weight that it will be given to the 'coverage' (CV) of the proportion of marker/allele combinations. When setting '`-CV 1`' Core Hunter II will optimize (maximize) only the diversity of marker/allele combinations; in that case the output shows the proportion of marker/alleles from the full collection present in the selected set; a $CV = 1$ implies that all marker/alleles from the full collection were present in the selected set.

Given the stochastic components in the search performed by Core Hunter II, each run whit identical parameters can lead to different solutions, i.e., different sets of selected accessions, even if the optimization criteria are the same for all sets in the output. Given this, we performed ten runs of Core Hunter II with

each set of parameters. For the sizes of the full collections tested, the maximum running time of Core Hunter II, controlled by the parameter '`-runtime`' made no difference (results not shown), and thus was let to its default of 60 seconds. Given the deterministic nature of AMA, there are not differences in the output when the input is determined, and there are not parameters to try, thus only one run for each set of data was performed with function AMA. Comparisons of AMA with Core Hunter II include coverage, CV, size of the selected set, $\#S$', average rareness in the selected set, $\bar{\rho}_S$ and statistics for the size of intersection between the set selected by AMA and each one of the set selected by CoreHunter II.

The set $\mathcal{G}$ selected by AMA from the 240 accessions of PL, PA and TE has 40 accession (see row PL∪PA∪TE in Table S1-10) and an average rareness of $\bar{\rho}_S = 0.7289 \approx 0.73$, that compared with the average rareness of the full collection, $\bar{\rho} = 0.6696$, presents a difference of $\bar{\rho}_S - \bar{\rho} = 0.7289 - 0.6696 \approx 0.06$ or 6%.

We ran Core Hunter II with decreasing `-sample_intensity`, say, 0.2, 0.16 and 0.1584, corresponding to $0.2 \times 240 = 48$, 39 and 38 accessions selected, respectively. Results are presented in Table S1-12.

Table S1-12. Results of running Core Hunter in the collection PL, PA and TE with `-CV 1` and decreasing `-sample_intensity`. Each row presents results for 10 independent runs.

`-sample_intensity`	$\#S$	CV	$\bar{\rho}_S$	min(I)	$avg(I)$	max(I)
0.2000	48	1.0000	0.7091	27	30.80	34
0.1600	39	1.0000	0.7200	31	33.00	35
0.1584	38	0.9964	0.7193	30	31.60	33

From Table S1-12 we can see that the minimum size of the group of accessions with a complete coverage (column 'CV') is given by 39 accessions; all 10 sets obtained by running with `-sample_intensity=0.1584` (third row) give $CV = 0.9964 < 1$. We take this as evidence that the size of the optimum set, $\#(\mathcal{O})$; $\mathcal{O} \in \mathbb{O}$, in this particular case is equal to 39 accessions. In comparison, the set of accessions found by AMA has 40 accessions, only one more than the optimum of 39, representing less than 0.5% of the total number of accessions. With this we demonstrate that the size of the set of accessions selected by AMA is close to the one in the optimum, say $\#(\mathcal{G}) \approx \#(\mathcal{O})$. Also from Table S1-12 we can note that the average rareness of the sets obtained with Core Hunter II, column $\bar{\rho}_S$, are smaller than the average rareness of the accession obtained by AMA, 0.7289. In fact, none of the individual sets obtained by Core Hunter II is as large as this value (data not shown). The statistics for the length of the intersection between $\mathcal{G}$ –the solution with 40 accessions found by AMA, and each one of the sets found by Core Hunter (columns min(I), $avg(I)$, max(I), corresponding to the minimum, average and maximum of the length of intersections, respectively), show that for all sample intensities, all sets found by Core Hunter are distinct to the one found by AMA in the sense that these do not contain $\mathcal{G}$ as subset. If $\mathcal{G}$ were subset of any of the sets found by Core Hunter, then the maximum of the intersection, max(I), must be equal to 40 or $\#S$ (the size of the Core Hunter solutions). In Table S1-12 we see that max(I) is 34 for $\#S = 48$, so none of the Core Hunter solutions contains $\mathcal{G}$. Also max(I) is 35 and 33 for $\#S$ equal to 39 and 38 respectively, thus the solutions found by Core Hunter are not subsets of $\mathcal{G}$. The average lengths of intersections are around 32 accessions, i.e., only approximately 32/40 or 80% of the accessions are shared between $\mathcal{G}$ and the Core Hunter solutions.

For *ex-situ* conservation proposes, it is important to define an optimum set of accessions in a given geographic area. In this case it was necessary to find the optimum set of accessions only within the state of Puebla. We tested the AMA and CoreHunter algorithms with the set of the 185 accessions from Puebla (PL). Within the context of the PL set, the rareness coefficient, ρ, varied from 0.5541 to 0.8120 with a mean of 0.6740. The set selected by AMA comprises 30 accessions with a mean rareness $\bar{\rho} = 0.6932$ As before, we run CoreHunter maximizing full coverage with decreasing sampling intensities (`-sample_intensity`) and determined that with `-sample_intensity` < 0.163 the results were sets with less than 30 accessions but with incomplete coverage ($CV < 1$). Thus in this case AMA gives a selected

set with the maximum coverage ($CV = 1$) which is also optimum by presenting the minimum set size that can give full coverage. Because this case is the one with highest practical importance, we made 100 runs of CoreHunter with parameters `-sample_intensity 0.163 -CV 1`. In all cases the selected sets contained 30 accessions, but the mean rareness coefficient for the CoreHunters sets was $\bar{\rho} = 0.0.6885$, confirming that AMA tend to produce a selected set with higher average rareness ($\bar{\rho} = 0.6932$ in this case).

To corroborate that AMA give near optimum results in coverage, and larger rareness in the selected set than Core Hunter, we analyzed a dataset of maize accessions, genotyped at 24 SSR loci with 186 total alleles obtained by fingerprinting 275 bulks of maize landrace populations, each containing multiple maize individuals from the Americas and Europe [6]. Here this dataset is named '`bulc`' (by the name of the file downloaded from www.corehunter.org as example for Core Hunter version 2.0).

For the `bulc` dataset, with 275 accessions and an average rareness $\bar{\rho} = 0.1618$, AMA gave a set of 19 accessions with an average rareness $\bar{\rho}_S = 0.1585$ Table S1-13 presents the results of running Core Hunter in the `bulc` dataset.

Table S1-13. Results of running Core Hunter in the `bulc` dataset with `-CV 1` and decreasing `-sample_intensity`. Each row presents results for 10 independent runs.

`-sample_intensity`	$\#S$	CV	$\bar{\rho}_S$	$\min(I)$	$avg(I)$	$\max(I)$
0.2000	55	1.0000	0.1603	5	7.30	11
0.0700	19	1.0000	0.1542	4	7.30	11
0.0640	17	1.0000	0.1591	7	8.70	11
0.0580	16	0.9893	0.1567	4	6.70	8
0.0350	9	0.9247	0.1472	3	3.40	4

From Table S1-13 we can see that the smallest size of sets with full coverture ($CV = 1$) selected by Core Hunter II have 17 accessions, this is 2 less than the set selected by AMA. This represents an improvement of approximately 0.7% with reference of the complete set of 274 accessions in the full collection. However, for selected sets of 19 accessions, the average rareness of the sets selected by Core Hunter, $\bar{\rho}_S = 0.1542$, is smaller than the average rareness of the sets selected by AMA, $\bar{\rho}_S = 0.1585$, confirming that for sets of the same size and near the optimum size, AMA tents to select rarest accessions.

From the results presented in this section we conclude that the AMA algorithm gives near optimal sets of complete coverage, i.e., including all marker/allele combinations present in the full collection. This selection can help when taking decisions for the conservation of *ex-situ* collections.

S1-2.6. **Estimating relations between maize phenotype and genetic profile.** Maize accessions in the GenoMaiz database include geographic and phenotypic information, as for example the place where the accession is currently grown (and was collected), the type of maize, the kernel color, among other. Here we present exploratory analyses of relation between the genetic profile and those characteristics.

To find association between a qualitative characteristic of maize –as race or kernel color, with the genetic profile represented by the vector of numbers of bulks that present a given marker/allele (genetic profile in z scale), we can perform two kinds of analysis. The first is to consider z as lineally related with the frequencies of the marker/alleles and then preform a t-test or an ANOVA taking the qualitative characteristic as a factor. The second is to consider the presence / absence of each marker allele in a bulk, and construct a contingency table to assess the dependence of the qualitative characteristic on the presence of the marker/allele. This second approach is briefly presented in the next section.

S1-2.6.1. *Testing marker/allele presence versus a qualitative character.* Consider the problem of finding marker/allele combinations (denoted simply as m in what follows) associated with qualitative characteristics of the accession, say q. To detect association between sets $\{m_i\}$ and $\{q_j\}$, we can consider a

Table S1-14. General form of the r x k contingency table to detect association between sets $\{m_i\}$ and $\{q_j\}$

	q_1	q_2	$\cdots$	q_k	Total
m_1	$n_{1,1}$	$n_{1,2}$	$\cdots$	$n_{1,k}$	$\sum_j n_{1j}$
m_2	$n_{2,1}$	$n_{2,2}$	$\cdots$	$n_{2,k}$	$\sum_j n_{2j}$
$\cdots$	$\cdots$	$\cdots$	$\cdots$	$\cdots$	$\cdots$
m_r	$n_{r,1}$	$n_{r,2}$	$\cdots$	$n_{r,k}$	$\sum_j n_{rj}$
Total	$\sum_i n_{i1}$	$\sum_i n_{i2}$	$\cdots$	$\sum_i n_{ik}$	$\sum_{i,j} n_{ij}$

'contingency table', as the one presented in Table S1-14. To find association between rows and columns of Table S1-14 we consider the probabilities of rows, p_i, and columns, p_j, and set as null hypothesis

$$\mathcal{H}_0 : p_{ij} = p_i p_j$$

for all pairs $\{i, j\}$. The alternative hypothesis is

$$\mathcal{H}_a : p_{ij} \neq p_i p_j$$

which implies that the classification criteria are dependent (see for example [9]). Under $\mathcal{H}_0$ the expected values of each one of the cells in Table S1-14 are given by

$$E[n_{ij}|\mathcal{H}_0] = \frac{\sum_i n_{ij} \sum_j n_{ij}}{\sum_{i,j} n_{ij}}$$

$\mathcal{H}_0$ can be tested with the Likelihood Ratio Test (LRT) or 'G test' [29] for contingency tables, which follows an approximate χ^2 distribution with $df = (r-1)(k-1)$ degrees of freedom or, mainly for the case of 2×2 contingency tables, by using Fisher's exact test [11].

S1-2.6.2. *Maize race.* The variable '`tipo`' in the GenoMaiz database gives the type of maize as classified by the collectors, and corresponds to maize 'race' (see for example [14, 31]). The analysis in this section has as objective to explore the relation between the marker/allele combinations (genetic profile) and the race of the maize accessions. A total of 217 accessions, 185 from PL and 32 from PA have information for a total of 257 marker/allele combinations (see Table S1-1 for the number of alleles per marker in the PL∪PA set).

As a first step in the analysis we estimated the differences in GD 'Between' and 'Within' races using the function `dist.bet.wit` (see S1-3.1 in the Appendix for the code of the function). We found a mean GD of 15.33 'Between' races while the mean GD 'Within' race was 14.42 and the difference between these mean GD was highly significant ($P < 1 \times 10^{-100}$). This implies that the accessions of the same race are, in average, closer in GD to accessions from the same race than to accessions of other races.

Even when 17 races were present in the 217 accessions, only 8 of them were represented by 10 or more accessions; only these races were taken into account in further analyses. To investigate which of the marker/allele combinations were significantly associated with each race, we performed t tests for the means of each marker/allele combination in the sets formed, in turn, by the accessions of each race and all other accessions. The scale used to measure the marker/allele in the genetic profiles was z, the number of bulks that presented the marker allele, that can take values 0, 1, 2 and 3. We also performed contingency tables (CT) analyses for marker/allele combinations that were present in at least 50 of the bulks of the 217 accessions. This arbitrary threshold of 50 bulks for CT was set in order to avoid excessive data sparseness. 120 marker/allele combinations complied with this threshold and were analyzed in turn for each one of the 8 races using Fisher exact test [11]. As an example of the CT analyses, Table S1-15 presents the results for the marker/allele PHI96100_295 in the Palomero race.

In Table S1-15 the row labeled 'With' presents the number of bulks in which the marker/allele PHI96100_295 was present, while the row labeled 'Without' presents the number of bulks in which that marker/allele was absent. As can be seen from the table, the prevalence of PHI96100_295 is low (6/96 = 0.0625) in

Table S1-15. Contingency Table for the presence of marker allele PHI96100_295 in the ($217 \times 3 = 651$) bulks of the 217 accessions studied. P value in the Fisher's exact test for independence: 2.38×10^{-80}.

	Palomero	Others	Total
With	6	532	538
Without	90	23	113
Total	96	555	651

Palomero while it is high ($532/555 = 0.9586$) in other races, causing a very low and significant P value in Fisher's test (2.38×10^{-80}).

Given that many tests will be performed looking for association between race and marker/allele, the P values of the tests for both, the t-tests and Fisher's exact tests in CT, were transformed to 'False Discovery Rate' (FDR) using the R function `p.adjust` by the method of Benjamini and Hochberg [1]. Only marker/allele combinations with a FDR of 0.001 or 0.1% were considered as relevant or 'significant'.

Table S1-16 present a summary of the results for each race for both the t-test and CT, while Table S1-17 give –as examples, the most significant marker/allele for each race in the t-tests.

Table S1-16. Number of accessions and number of significant (FDR $\leq$ 0.1%) marker/allele combinations for each one of the eight races represented by at least 10 accessions. Results are presented for t-test and contingency tables (CT) analyses. Column 'Both' shows the number of marker/allele combinations significant in both test (t and CT).

Race	# Accessions	t-test			CT	Both
		#Sig.	#+	#−		
Ancho	13	27	6	21	5	1
Arrocillo	13	16	2	14	3	1
Chalqueno	18	17	4	13	2	1
Conico	52	3	1	2	9	3
Elotes Conicos	33	6	2	4	6	3
Palomero	32	52	13	39	53	41
Pepitilla	15	13	3	10	1	1
Tuxpeno	10	21	5	16	4	1
Totals:	186	155	36	119	83	52
Different marker/alleles:		88			64	47

From Table S1-16 we can see that between 3 (1.17%) –for 'Conico' and 52 (20.23%) for 'Palomero', of the 257 marker/allele combinations tested are significantly associated with one of the races (FDR $\leq$ 0.1%) by the t-test, while between 1 –for Pepitilla and 53 –for Palomero are significantly associated by the CT analyses. In total, 88 distinct marker/alleles were significant for at least one race in the t-test, even when adding the number of marker/alleles per race gives a total of 155, this figure that takes into account marker/alleles that appear in more than one race. For the CT analyses a total of 64 marker/allele combinations are significant for at least one race. The association between mean of marker/allele and race can be positive –when the marker/allele is present at a higher frequency in the race (column '#+'), or negative, when the opposite happens (column '#−'). Interestingly, for all races there are more negative than positive associations; i.e., the race is in most cases related with a lower than higher presence of the marker/allele. However, as we will see in Table S1-17, there are cases when the marker/allele is estimated at the maximum value (average $z = 3$) or completely absent (average $z = 0$). There is not a single marker/allele that appear in all 8 selected races as significant in the t-tests, however for all races some of the alleles of the marker 'PHI96100' are significantly associated with race (see Table S1-18), thus marker

'PHI96100' is the one that appears to be more discriminant for race in the accessions studied. Also a total of 47 marker/allele combinations were significant by both, the t-tests and the CT analyses. This demonstrates that there are genetic differences between races.

Table S1-17 presents the most significant marker/allele combination in the t-tests for each one of the eight races.

TABLE S1-17. Statistics for the most significant (smallest FDR) marker/allele combinations for each one of the races in the t-tests. Average values of z are presented for the race (column 'In race') and for all other accessions in the set PL∩PA (column 'In others').

Race	Marker_Allele	In race	In others	P	FDR
Ancho	PHI96342_230	0.0000	0.5147	6.02e-17	1.54e-14
Arrocillo	PHI031_219	0.0000	0.3480	6.62e-12	1.70e-09
Chalqueno	PHI034_120	3.0000	2.6231	6.43e-12	1.65e-09
Conico	PHI96100_295	2.9423	2.3333	5.36e-10	1.37e-07
Elotes Conicos	PHI015_80	0.2424	1.0597	5.21e-09	1.34e-06
Palomero	PHI96100_295	0.2500	2.8648	9.77e-23	2.51e-20
Pepitilla	PHI093_287	3.0000	2.6485	8.94e-13	2.29e-10
Tuxpeno	PHI109188_162	3.0000	2.0290	1.89e-24	4.86e-22

From Table S1-17 we can see that between the most significant marker/allele combination for each race in the t-tests we find two cases –for Ancho and Arrocillo, of marker/allele estimated to be absent of the accession, while there are three cases –for Chalqueno, Pepitilla and Tuxpeno where the corresponding marker/allele is estimated as with the maximum average value of $z = 3$; in these cases all bulks of all accessions are positive for the presence of the corresponding marker/allele, which indicates the likelihood of a high frequency for the corresponding allele in the accessions.

As mentioned before, marker 'PHI96100' appears to be the marker more discriminant for race. Table S1-18 presents the significant differences for frequencies of alleles of this marker at each one of the races.

From Table S1-18 we can see that seven of the 20 alleles from PHI96100 (see also Table S1-1) are among the ones with significant (FDR$\leq$ 0.001) differences in z scores between at least one of the 8 maize races. In three cases –alleles 271, 279 and 292, the alleles are un-detected in a race, while present in other accessions at non zero z values while in 2 cases (for alleles 275, and 288) these alleles are detected in all accessions (average of $z = 3$) in some races (allele 275 in Ancho and Tuxpeno; allele 288 at Ancho and Arrocillo). The fact that two different alleles, 275 and 278, appear in all three bulks of a single race (Ancho), indicates that the frequency of such alleles is high (average $z = 3$).

To further investigate the power of the marker PHI96100 to discriminate among races, we constructed a dendrogram based only in this marker for the accession of the selected races. That dendrogram is presented in Figure S1-11. From Figure S1-11 we can see that even when there is some tendency of accessions to cluster first by race (in particular for Palomero), the information from this marker is not enough to give good race classification (clustering); i.e., races are far from being well segregated by the information given by this marker, even when some alleles are highly specific to race (Table S1-18).

Figure S1-12 presents the dendrogram of all accessions –presented before in Figure S1-2, but colored by maize race.

From Figure S1-12 we can see that by using all information available, the only maize race that is well segregated is Palomero (PA), a fact that was previously observed and discussed here. All other 7 races are scattered into different clusters, even when some accessions of the same race tend to cluster first by pairs.

Table S1-18. Significant (FDR≤ 0.001) differences for frequencies of alleles of marker PHI96100 per race in t-tests. Average values of z are presented for the race (column 'In race') and for all other accessions in the set PL∪PA (column 'In others').

Allele (bp)	Race	In race	In others	P	FDR
268	Palomero	0.7812	1.8054	6.5e-06	4.3e-05
271	Palomero	0.0000	0.2595	2.5e-08	3.2e-07
275	Palomero	1.9688	2.9459	4.8e-06	3.2e-05
	Ancho	3.0000	2.7892	3.3e-07	7.0e-06
	Tuxpeno	3.0000	2.7923	3.3e-07	7.7e-06
279	Arrocillo	0.0000	0.0931	2.4e-05	0.00041
	Chalqueno	0.0000	0.0955	2.4e-05	0.00039
288	Ancho	3.0000	2.7402	1.9e-11	1.6e-09
	Arrocillo	3.0000	2.7402	1.9e-11	2.4e-09
292	Ancho	0.0000	0.3480	2.8e-08	1.1e-06
	Chalqueno	0.0000	0.3568	2.7e-08	1.5e-06
	Pepitilla	0.0000	0.3515	2.8e-08	1.5e-06
	Tuxpeno	0.0000	0.3430	2.9e-08	1.1e-06
	Conico	0.0385	0.4182	6.4e-06	0.00055
	Elotes Conicos	0.0303	0.3804	2.8e-06	0.00015
	Palomero	2.0625	0.0270	5.5e-13	1.1e-11
295	Palomero	0.2500	2.8649	9.8e-23	2.5e-20
	Ancho	2.9231	2.4510	6.3e-05	6.0e-04
	Conico	2.9423	2.3333	5.4e-10	1.4e-07

From the information presented in this section we conclude that there is a relation between race and genotype, taking this as the marker/allele profile. This was demonstrated by the large number of highly significant differences in the prevalence of particular marker/allele combinations when segregating accessions by race. However, maize races (with the exception of Palomero) are far from being genetically segregated, and this is possibly a result of the complex race inter breeding history.

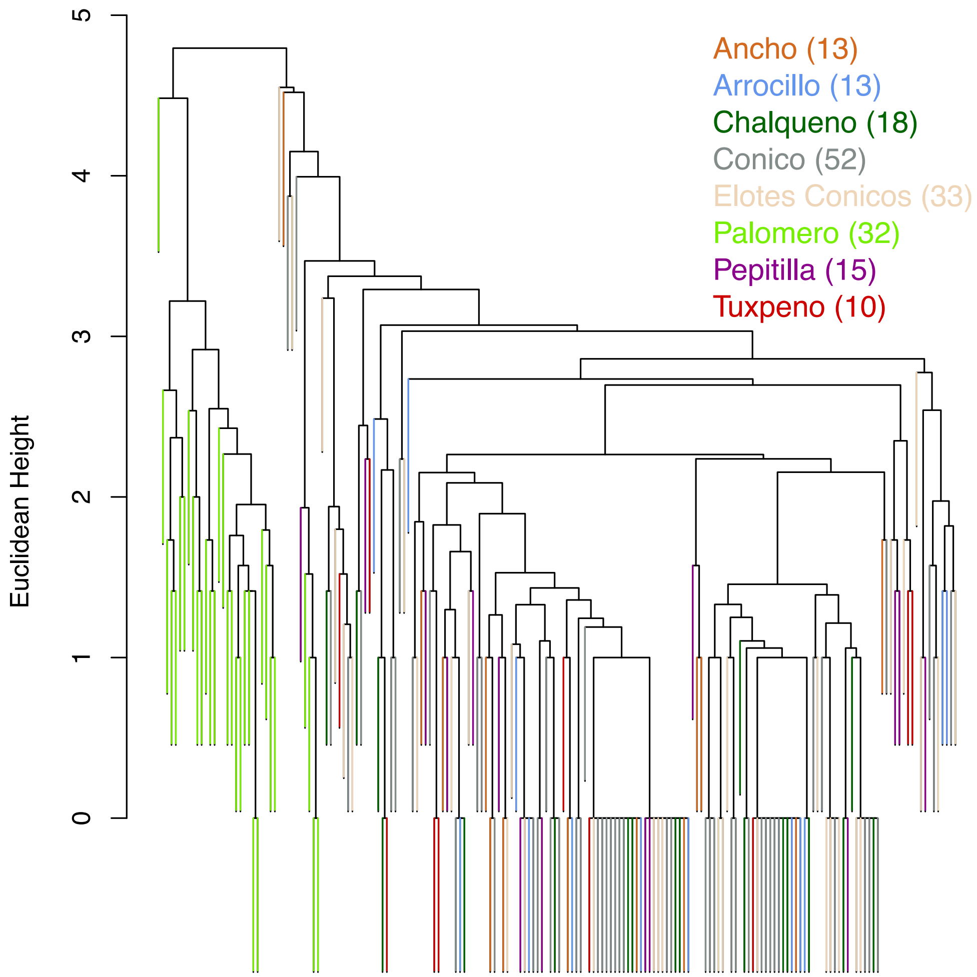

FIGURE S1-11. Dendrogram obtained by UPGMA of the Euclidean distance calculated only from data of the marker PHI96100 (20 alleles) in accessions from the eight selected races.

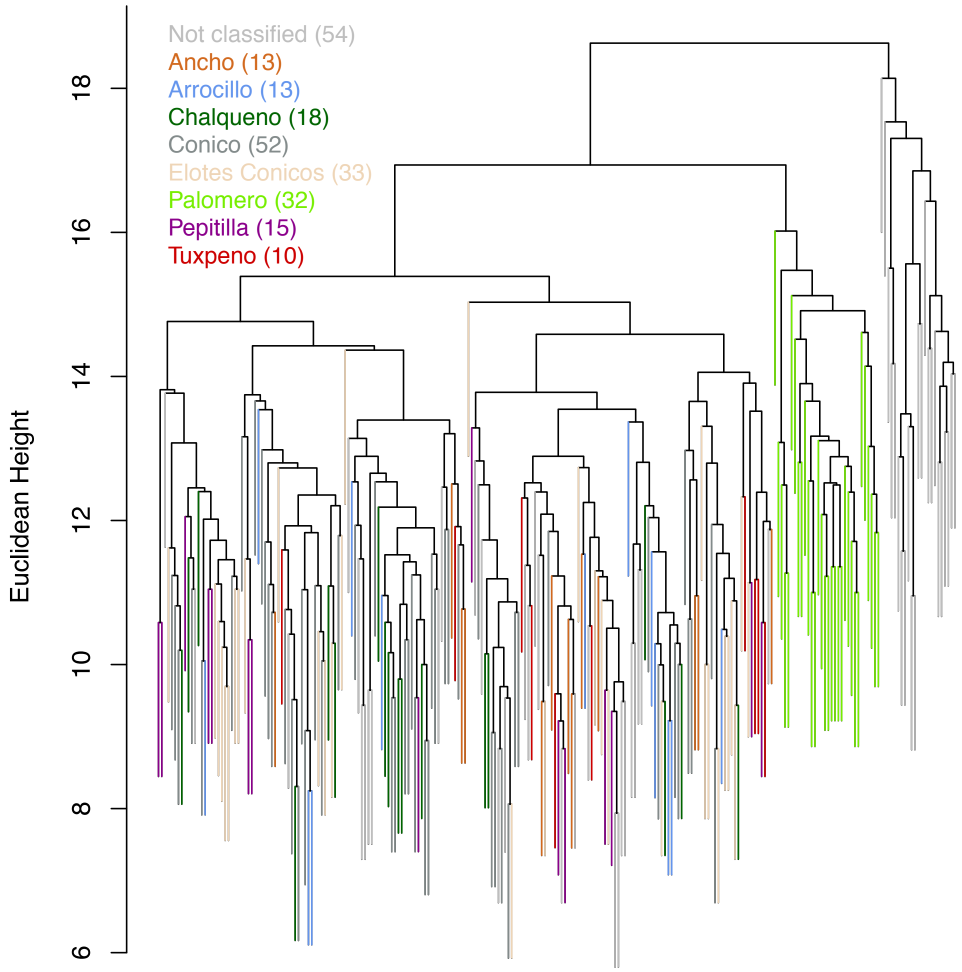

Figure S1-12. Dendrogram of 240 accessions colored by group of origin and obtained from the Euclidean distance of the accession's genetic profiles by UPGMA. Colors indicate maize race, for races represented by at least 10 accessions.

S1-2.6.3. *Kernel color.* Qualitative variable '`color`' in the database indicates the maize kernel color as perceived by the grower of the accessions. Because there are only 32 accessions from the PA type and this is a well genetically segregated race, we performed the analysis of genetic relation of `color` with genetic profile only for the 185 PL accessions. The variable `color` has 11 different values (colors), but we collapsed these categories into only 5 colors by grouping alike colors. The number of accessions by color are shown in Table S1-19.

Table S1-19. Number of accessions from PL by color.

Color	# Accessions
'Amarillo' = Yellow	15
'Blanco' = White	108
'Azul' or 'Negro' = Dark Blue	35
'Rojo' (plus variants) = Reddish	19
Other	8

As for the case of race, we made t-tests for the means of the z value (number of bulks per accession) for each one of the 236 marker/allele combinations in the groups formed by accessions from a specific color (Table S1-19) and the group of other colors. To take into account multiple testing, we used a FDR ≤ 0.001 (as in the case of race). Table S1-20 shows the number of significant (FDR $\leq 0.1\%$) marker/alleles associated with color in the PL accessions while Table S1-21 show the most significant (smallest FDR) marker/allele combination for each color.

Table S1-20. Number of accessions and number of significant (FDR $\leq$ 0.1%) marker/allele combinations for each one of the colors represented by at least 10 accessions.

Color	# Accessions	# Sig. marker/allele	#+	#−
Yellow	15	8	0	8
White	108	2	2	0
Dark Blue	35	0	0	0
Reddish	19	1	0	1
Totals:	177	11	2	9
Different marker/alleles: 10 (4.24%)				

Table S1-21. Statistics for the most significant (smallest FDR) marker/allele combinations for each one of the colors in the t-tests. Average values of z are presented for the race (column 'In color') and for all other accessions in the set PL∩PA (column 'In others').

Color	Marker/allele	In color	In others	P	FDR
Yellow	PHI015_72	0.0000	0.2470	8.25e-09	1.95e-06
White	PHI031_218	2.1667	1.3117	5.72e-08	1.35e-05
Dark Blue	PHI064_74	0.4286	1.1800	7.61e-06	0.0018
Reddish	PHI015_108	0.0000	0.1687	2.97e-06	0.0007

From Table S1-20 we can see that only 11 marker/alleles (10 different) were significantly associated with the color of the accessions; this contrasts with the case of race, where we found a large number of marker/allele combinations, 155 (88 different) associated with the character (see Table S1-16). All 8 marker/allele combinations associated with 'Yellow' are negative (column '#−') indicating smaller frequencies in accessions with kernel of that color than in accessions with other colors, while two positive associations are present for 'White' accessions, the largest of the groups analyzed. Not significant (FDR < 0.001) associations were found for the 'Dark Blue' color –even when Table S1-21 presents the most

significant marker/allele combination for this color, that almost reached the threshold FDR < 0.001 and only one significant marker/allele combination was detected for 'Reddish' (last row of Table S1-21).

Figure S1-13 presents the dendrogram of the PL accessions colored by kernel color.

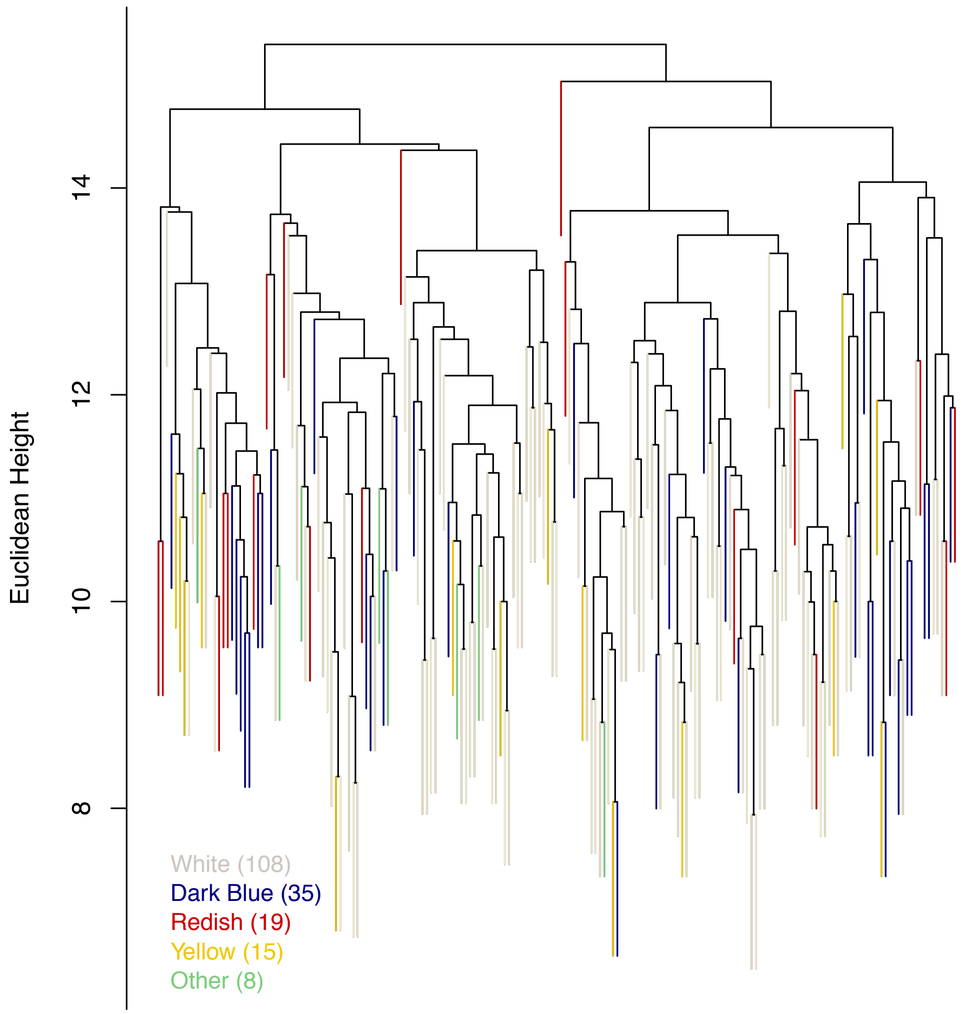

FIGURE S1-13. Dendrogram of 185 PL accessions colored by kernel color (Euclidean distance processed by UPGMA).

From Figure S1-13 we can observe that there is not a strong cluster tendency by kernel color; even accession grouped by pairs at low euclidean heights –say below 10 in the Y-axis of the dendrogram, are often of different colors, and there are paired accessions from different colors at different heights in the dendrogram.

Given the low number of marker/allele combinations significantly associated with kernel color (Table S1-20), and the lack of clear clustering by color structure in Figure S1-13, we conclude that kernel color is not strongly associated with genetic profile.

S1-2.6.4. *Correlation between geographic and genetic distances.* Correlation between geographic and genetic distances has been well documented for natural populations (see [21] for maize, [13] for a fungus, and [26] for an interesting case in humans). Here we used and tested Pearson's correlation coefficient r

[10] as well as Spearman's rank correlation coefficient ρ [16] to study the relation between the geographic and genetic distances between accession in our dataset.

A total of 232 accessions in the group PA∪PL∪TE had geographic localization and those were employed to calculate geographic as well as genetic distances. Table S1-22 presents statistics for genetic distances for the full set and various subsets, while Table S1-23 presents the same statistics for geographic distances in the same groups. In tables S1-22 and S1-23 the column '# Acc.' gives the number of accessions of

Table S1-22. Statistics for genetic distances (z scale) between all accessions per group.

Group	# Acc.	# Dist.	Min.	1st.Qu.	Median	Mean	3rd.Qu.	Max.	S
PA∪PL∪TE	232	26796	7.94	14.35	15.68	15.78	17.20	22.45	2.11
PA∪PL	209	21736	7.94	14.07	15.20	15.13	16.31	20.90	1.71
PA	25	300	11.00	13.29	14.54	14.48	15.68	18.44	1.55
PL	184	16836	7.94	13.75	14.76	14.65	15.68	19.29	1.50
TE	23	253	10.95	15.49	16.64	16.46	17.69	20.17	1.64

Table S1-23. Statistics for geographic distances (km.) between all accessions per group.

Group	# Acc.	# Dist.	Min.	1st.Qu.	Median	Mean	3rd.Qu.	Max.	S
PA∪PL∪TE	232	26796	0	81	121	155	195	578	115
PA∪PL	209	21736	0	69	108	110	143	357	60
PA	25	300	0	83	125	140	230	337	87
PL	184	16836	0	61	104	102	131	303	56
TE	23	253	0	55	95	96	124	233	54

the corresponding group, while '# Dist.' gives the number of distances calculated for all possible pairs of accessions within the corresponding group. The range of geographic distances for the whole group (PA∪PL∪TE; Table S1-23) goes from 0 to 578 km. with a mean of 155 and a standard deviation of 115 km. Naturally all ranges for the subgroups (PA∪PL, PA, PL and TE) are within the range for the full group, TE being the most geographically close set with a range from 0 to 203 km. and a mean of 96 km. and also the less variable with a value of standard deviation of 54. In contrast, from Table S1-22 we can see that the TE set is the one with largest median and mean genetic distances, 16.64 and 16.46 respectively, as well as the most variable of the individual sets (PA, PL and TE) with $S = 1.64$

To study the correlation between geographic and genetic distances we employed the R function `cor.test`, which test for association between paired samples, using one of Pearson's product moment correlation coefficient, r, or Spearman's ρ. While Pearson's r measures the degree of linear dependence between the distances, and r^2 measures the proportion of the variance of one of the distances explained by the other, Spearman's ρ is a nonparametric measure of dependence between the ranks of the distances. Table S1-24 presents the correlations between geographic and genetic distances for each group.

From Table S1-24 we can see that, in the full group (PA∪PL∪TE), $r = 0.6239$ and $r^2 = 0.3892$, and the null hypothesis of no-correlation, say $R = 0$, is rejected with high significance ($P < 2.2e - 16$). This indicates that, globally, around 39% of the variation of one of the distances can be explained by the other ($r^2 = 0.3892$). Spearman's correlation for the full group is $\rho = 0.5438$, lower that the value of r and possibly more representative of the true relation of the geographic with genetic distances. Figure S1-15 presents an scatter plot of a sample of 1000 of the geographic and genetic distances in the full group. From this figure we can appreciate that, even when there is an indubitable trend of increasing genetic distance as function of the geographic distance, the relation is highly noisy –i.e., to the same geographic distance correspond a large range of genetic distances. This is a result of the fact that maize accessions are human transported, and not naturally dispersed. To confirm this, we can examine the correlation between distances of subsets of the full group. In the row corresponding only to maize accessions (PA∪PL

Table S1-24. Correlations between geographic and genetic distances between accessions. 'LL' and 'UL' are the approximate 99% upper and lower confidence limits for r. 'P' are P-values for the test of the hypothesis of null (0) correlation.

Group	# Dist.	Pearson's					Spearman's	
		r	r^2	P	LL	UL	ρ	P
PA∪PL∪TE	26796	0.6239	0.3892	$< 2.2e-16$	0.6142	0.6334	0.5438	$< 2.2e-16$
PA∪PL	21736	0.2464	0.0607	$< 2.2e-16$	0.2299	0.2627	0.2453	$< 2.2e-16$
PA	300	0.1465	0.0215	0.0111	-0.0019	0.2886	0.1410	0.0145
PL	16836	0.1382	0.0191	$< 2.2e-16$	0.1187	0.1577	0.1526	$< 2.2e-16$
TE	253	0.5907	0.3489	$< 2.2e-16$	0.4745	0.6867	0.5782	$< 2.2e-16$

in Table S1-24) we see that $r = 0.2464, r^2 = 0.0607, \rho = 0.2453$; i.e., the correlation between the distances is less than half than the one estimated in the complete group. Also, the correlation in the group PA∪PL is highly influences by the facts that, as we have seen, PA is an ancient and well segregated maize race and the collection sites for PA and PL are far away. To discard these kind of artifacts we can study the correlations within individual sets (rows three to five in Table S1-24). Comparing these rows we note that the largest correlation by far is for accessions of the TE group; $r = 0.5907, r^2 = 0.3489, \rho = 0.5782$. These values are much larger that the ones corresponding to the PA ($r = 0.1465, r^2 = 0.0215, \rho = 0.1410$) or PL ($r = 0.1382, r^2 = 0.0191, \rho = 0.1526$). This means that the natural populations of TE are consistent with a regular decrease of genetic similarity with increasing geographic distance, as predicted by the theory of isolation by distance [20], while in the domesticated maize accessions (PA and PL) this effect is lost by human intervention in the dispersion of the races.

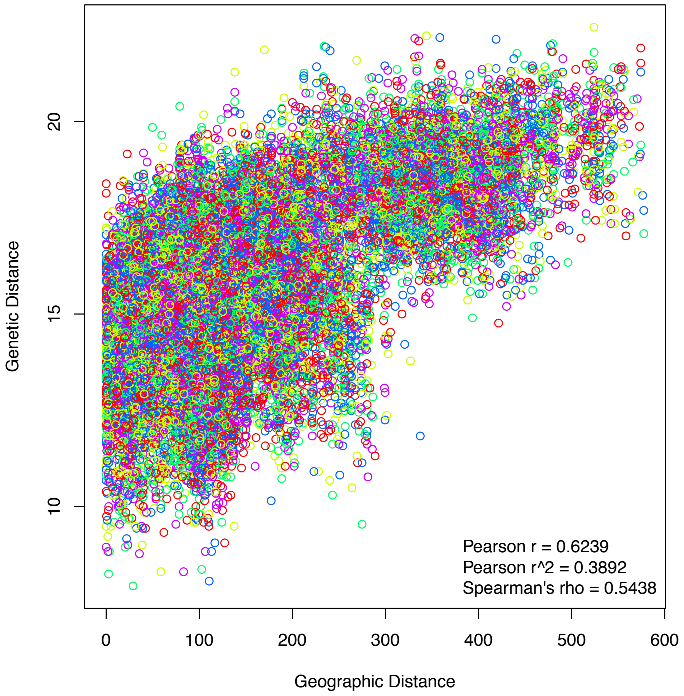

Figure S1-14. Scatter plot of a random sample of 1000 geographic (X-axis) × genetic (Y-axis) distances from the total of 26796 in the group of accessions PA∪PL∪TE.

Figure f14 presents a scatter plot of all the distances between TE accessions. From this figure we can notice the tendency of genetic distance to increase as a function of geographic distance, and this relation is less noisy than the one presented in Figure S1-13 for the complete set. Also, the value of ρ in the TE

group (0.5782), is the largest one in all the groups (Table S1-24), indicating a strongest relation of the ranked geographic and genetic distances in the TE than in the other collections.

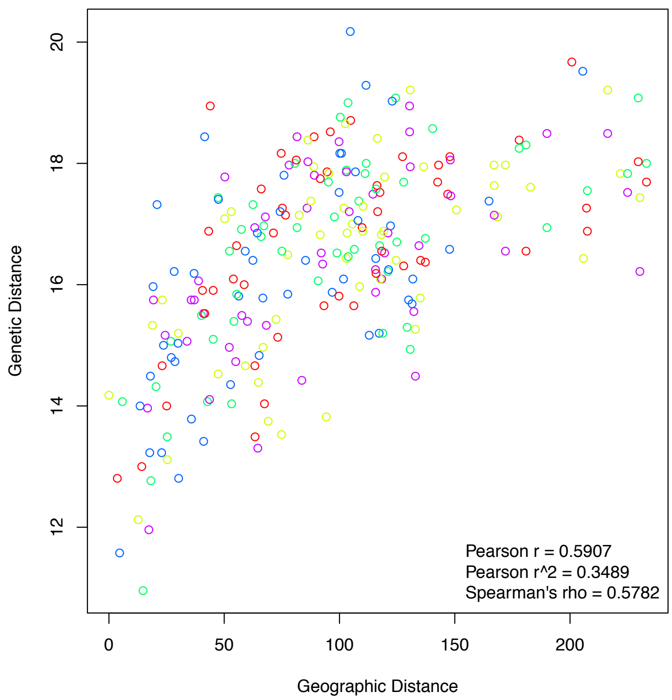

FIGURE S1-15. Scatter plot of geographic (X-axis) × genetic (Y-axis) distances from the group of 23 TE (all 253 distance pairs ploted).

S1-2.6.5. *Height above sea level (HASL).* The effect and race adaptation of maize to distinct height above sea level (HASL) is a well known fact [17]. Here we investigate if there is a relation between the presence of marker/allele combinations –measured in the z scale of number of bulks that contain the combination, with the HASL at which the accessions are grown and were collected. Two hundred and forty accessions of the group PA∪PL∪TE included data for HASL and were included in the analysis. Table S1-25 presents the statistics for HASL in these accessions. From Table S1-25 we see that HASL in the accessions of

TABLE S1-25. Statistics for height above sea level (HASL) in meters per group.

Group	# Acc.	Min.	1st.Qu.	Median	Mean	3rd.Qu.	Max.	S
PA∪PL∪TE	240	0	1290	2141	1872	2414	3025	788
PA	32	0	0	2118	1579	2582	2668	1117
PL	185	163	1348	2325	1988	2473	3025	705
TE	23	540	812	1465	1351	1848	2150	554

PA∪PL∪TE range from zero (sea level) up to 3025 m with a mean of 1872 and a standard deviation of 788, thus there is an ample representation of altitudes in the collection. Also from Table S1-25 we can see that the mean in groups PA, PL and TE differs. To test these differences we performed an ANOVA of HASL taking the groups PA, PL and TE as factors. This analysis gave an F value of 9.96 with 2 and 237 df which is highly significant ($P = 7.05e - 05$). Figure S1-16 shows the 95% confidence intervals for the pair differences of the means in the three groups. From this figure we can note that the difference between the means in the groups PL-PL and TE-PL are different from zero, while the difference between TE and PA is not.

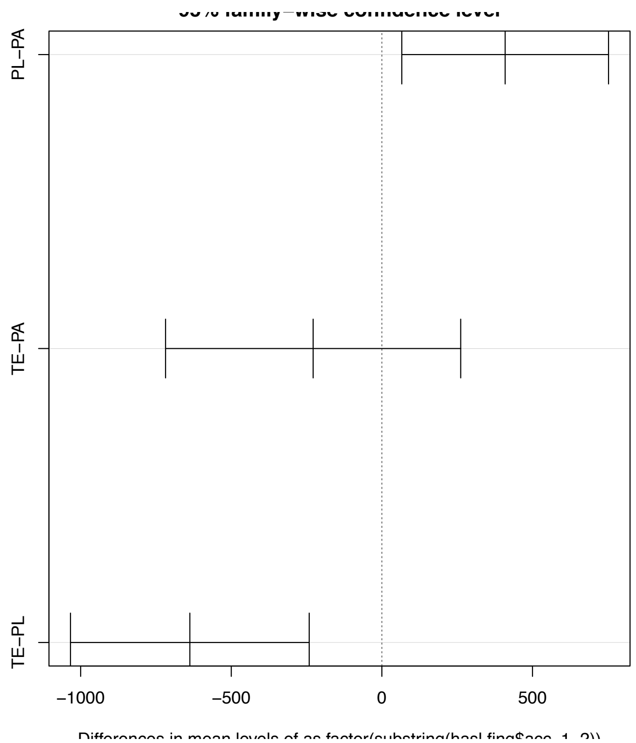

FIGURE S1-16. Tukey 'Honest Significant Differences' 95% intervals for the differences in HASD between groups of accessions.

To assess the relation between marker/allele combinations with the HASL we fitted linear models of the form

$$y_i = \alpha + \sum_{j=1}^{k} \beta_j z_{ij} + \varepsilon_i$$

where y_i is the value of HASL in the $i-th$ accession, $i = 1, 2, \cdots, 240$, z_{ij} is the value of the marker/allele combination in the $i - th$ accession and $j - th$ marker-allele, α is the intercept of the model and β_j the effect of the $j - th$ marker-allele while ε_i is the error or residual for the $i - th$ accession and k is the number of markers fitted in the model. To avoid high influence outliers, we fitted model including only marker/alleles with a sum of marker/alleles equal of larger than 40; i.e., marker/alleles that were present in at least 40 bulks. This criterion limited the number of marker/alleles to be fitted to 140 of the 278 initially available (see Table S1-1).

First a 'full' model was fitted by including all 140 marker/alleles. This model was highly significant ($P = 2.697e-10$), having a residual standard error: 506.7 on 99 degrees of freedom, a multiple R-squared of 0.8286 and an adjusted R-squared of 0.5863 Looking for a more parsimonious model, we subjected the full model to the Stepwise Algorithm by AIC trough the R function '`step`' with default parameters (see for example [22]). The resulting model –called here 'step model', contained 55 marker/allele combinations for all the 14 markers, having a residual standard error of 412.9 on 184 degrees of freedom with a multiple R-squared of 0.7885, and adjusted R-squared of 0.7253. The F-statistic for this model was 12.47 on 55 and 184 df and $P < 2.2e-16$. The step model demonstrates that there is a high genetic component explaining around 73% of the total variance of HASL, as function of the z values of 55 of the 140 marker/alleles; i.e. $55/140 \approx 0.39$ or 39% of the marker/alleles have some effect on HASL. Table S1-26 and its continuation in Table S1-27 present the coefficients for the step model.

From Tables S1-26 and S1-27 we can see that the step model includes at least one (for PHI093) and as much as seven (for PHI034) alleles; i.e., all 14 markers included in the study appears to have some influence over HASL. The 55 estimated coefficients (column 'Estimate ($\hat{\beta}$)' in Tables S1-26 and S1-27)

Table S1-26. Coefficients and statistics for the 'step model'.

Marker	Allele	Estimate ($\hat{\beta}$)	Std. Error	t-value	$Pr(>\|t\|)$
(Intercept)		$\hat{\alpha} = 2965.21$	386.93	7.663	1.01e-12
PHI015	80	-90.01	33.47	-2.689	0.007822
	87	-80.83	33.91	-2.384	0.018150
	95	-221.85	44.67	-4.966	1.55e-06
	101	170.17	38.74	4.392	1.89e-05
PHI031	190	-92.97	44.03	-2.112	0.036079
	195	-118.32	37.97	-3.117	0.002124
	202	-118.97	63.69	-1.868	0.063361
	209	84.78	37.39	2.267	0.024524
	218	-73.10	29.19	-2.504	0.013148
	219	-141.60	47.58	-2.976	0.003312
	222	-81.69	44.06	-1.854	0.065314
PHI033	236	-85.83	35.00	-2.452	0.015127
	239	140.73	39.73	3.542	0.000502
	246	-106.19	33.41	-3.178	0.001739
	260	53.28	36.85	1.446	0.149919
PHI034	112	-129.60	32.56	-3.981	9.88e-05
	113	-101.52	41.70	-2.434	0.015873
	127	-167.37	60.92	-2.747	0.006609
	132	-160.36	35.99	-4.455	1.45e-05
	136	84.81	40.71	2.083	0.038606
	138	83.52	47.10	1.773	0.077863
PHI051	136	-105.16	44.91	-2.341	0.020279
	138	-136.76	44.17	-3.096	0.002267
	141	-92.69	38.30	-2.420	0.016499
PHI053	136	-127.83	72.99	-1.751	0.081553
	185	-116.18	37.17	-3.126	0.002062
	189	117.10	37.33	3.137	0.001991
PHI064	69	125.25	36.86	3.398	0.000832
	73	142.00	55.63	2.553	0.011500
	78	-114.59	59.91	-1.913	0.057313
	90	-118.96	64.89	-1.833	0.068394
PHI072	143	79.72	36.76	2.168	0.031404
	148	-130.90	39.01	-3.355	0.000963
	152	171.25	43.03	3.979	9.93e-05
	160	-87.17	40.52	-2.151	0.032781
PHI093	287	74.29	46.60	1.594	0.112645
PHI109188	120	110.08	40.86	2.694	0.007716
	145	147.22	28.47	5.172	6.02e-07
	166	73.24	32.09	2.282	0.023629
	173	-119.52	48.34	-2.473	0.014320
	175	-88.11	53.18	-1.657	0.099298
PHI127	112	168.85	64.61	2.613	0.009705
	114	-54.08	31.43	-1.720	0.087035
	118	-69.57	31.76	-2.190	0.029770
	123	87.64	31.67	2.767	0.006229
PHI427913	115	-85.27	41.49	-2.055	0.041277
	117	119.14	61.74	1.930	0.055164
PHI96100	271	-158.90	54.57	-2.912	0.004038
	275	-104.56	60.84	-1.719	0.087379
	292	-330.60	68.01	-4.861	2.50e-06

Table S1-27. Coefficients and statistics for the 'step model' (Continue from Table S1-26).

Marker	Allele	Estimate ($\hat{\beta}$)	Std. Error	t-value	$Pr(>\|t\|)$
	230	182.88	49.71	3.679	0.000307
	233	-72.14	45.53	-1.584	0.114800
PHI96342	235	151.13	65.58	2.304	0.022319
	242	105.37	50.61	2.082	0.038739
	208	-89.92	56.85	-1.582	0.115464

indicate the average increase (when positive) or decrease (when negative) of the mean of HASL. 21 (38%) of such coefficients are positive and the remaining 34 (62%) are negative; thus there are fewer marker/alleles positively associated with HASL than negatively associated. The sizes of the estimated effects go from -330.60 for PHI96100_292 up to 182.88 for PHI96342_230 with a mean for all coefficients of -27.28 The individual P values for the tests of the hypothesis of no effect ($\beta_i = 0$) in column $Pr(> |t|)$, have a highly significant minimum of $1.55e - 06$ for PHI015_95 and a not-significant maximum of 0.1499 for PHI033_260.

To find a model with the most relevant marker/alleles, we visually examined each one of the distributions of HASL as function of the presence of 0 to 3 bulks (z) in each one of the 55 marker/alleles present in the step model. From these distributions we selected only the marker/alleles which gave an almost monotonically decrease or increase in the median of HASL. This procedure yield the selection of seven markers which were fitted into a 'final model'. This final model was highly significant ($P < 2.2e - 16$), with a residual standard error of 590.4 on 232 degrees of freedom, a multiple R-squared of 0.4548 and an adjusted R-squared of 0.4384; thus the decrease from 55 marker/alleles in the step model to 7 marker/alleles (87%) in the final model only decreased the adjusted R-squared from 0.7253 to 0.4384 (40%). This final model is easier to interpret and more parsimonious because all effects of the marker/alleles are a good lineal approximation to the changes in HASL. Table S1-28 presents the coefficients for the final model.

Table S1-28. Coefficients and statistics for the 'final model'.

Marker	Allele	Estimate ($\hat{\beta}$)	Std. Error	t-value	$Pr(>\|t\|)$
(Intercept)	$\hat{\alpha} = 1113.11$		168.07	6.623	2.42e-10
PHI015	80	-126.99	38.74	-3.278	0.001205
PHI031	190	-151.06	45.40	-3.328	0.001019
	195	-150.71	45.96	-3.279	0.001202
PHI015	101	176.86	45.57	3.881	0.000135
PHI093	287	196.53	54.68	3.594	0.000398
PHI109188	145	212.50	29.90	7.108	1.44e-11
PHI96342	230	157.23	49.94	3.148	0.001858

From Table S1-28 we can see that all fitted marker/alleles are significant; the maximum value of $Pr(> |t|)$ is 0.001858 for PHI96342_230. There are three negative and four positive effects over the mean of HASL going from -151.06 for PHI031_190 up to 212.50 for PHI109188_145 and with standard errors smaller than 55.

To visualize the effects of individual marker/alleles, figures S1-17 and S1-18 present the distributions of HASL as functions of the number of bulks (z) for two marker/alleles, one with positive effect, PHI109188_145 (Figure S1-17) and the other with a negative effect, PHI031_190 (Figure S1-18).

From Figure S1-17 we can see how the mean and median of HASL increases lineally with the number of bulks of the marker/allele PHI109188_145; even when the distributions are formed by different number of accessions (brown numbers) and there is a large dispersion for each group (length of boxes and whiskers). The model fitted including only this marker/allele (blue line) was highly significant ($P = 1.807e-12$) and

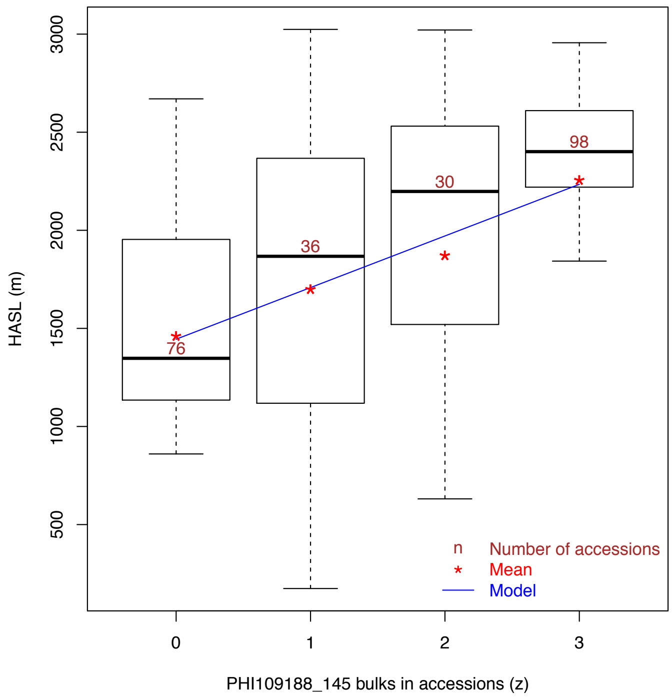

FIGURE S1-17. Distributions of HASL as function of the number of bulks of marker/allele PHI109188_145. Medians are shown as broad lines, while means are marked by red asterisks.

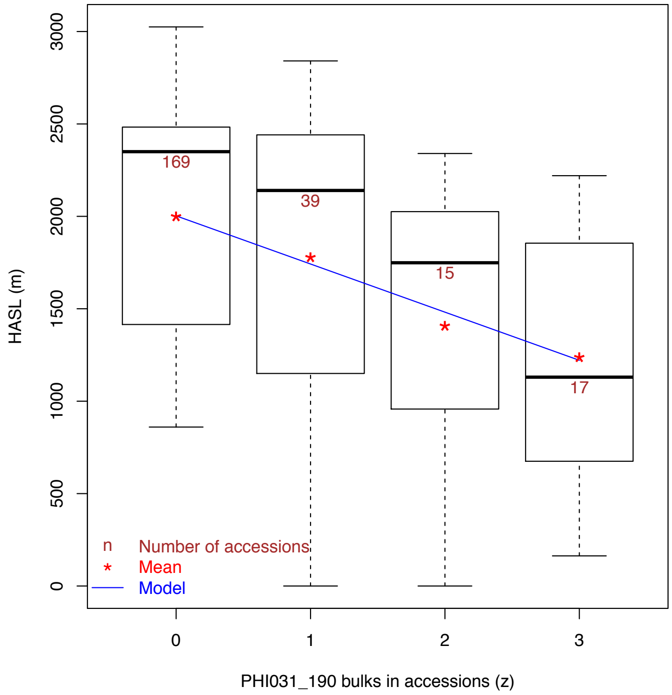

FIGURE S1-18. Distributions of HASL as function of the number of bulks of marker/allele PHI031_190. Medians are shown as broad lines, while means are marked by red asterisks.

explained approximately 19% of the total HASL variance (Adjusted R-squared 0.1853). In Figure S1-18 we can observe the distribution of HASL as function of the marker/allele PHI031_190. In this case the mean and median of HASL decrease lineally as function of the number of bulks containing PHI031_190. The model fitted to this relation (blue line) was highly significant ($P = 2.976e - 06$) and explained approximately 8% of the total HASL variance (Adjusted R-squared 0.08399).

Figure S1-19 presents a dendrogram of the 240 accessions colored by HASL. Colors correspond to quantiles 0% $\leq$ to $<$ 25% (red), 25% $\leq$ to $<$ 50% (green) to 50% $\leq$ to $<$ 75% (light blue) and 75% $\leq$ to $<$ 100% (violet).

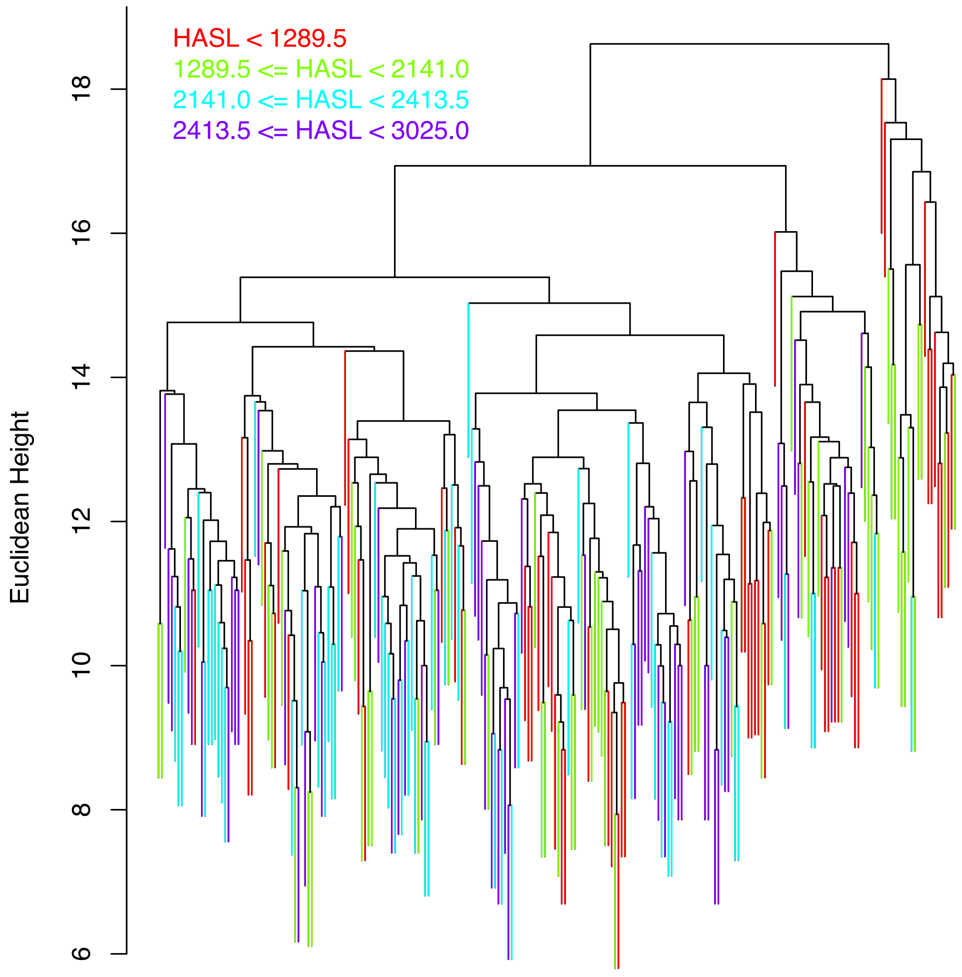

FIGURE S1-19. Dendrogram of 240 accessions colored by HASL. Euclidean distance, UPGMA (see Figure S1-2)

From Figure S1-19 we can see a tendency of the accessions to cluster by similar HASL, even when evidently other factors influence the genetic likeness.

From the results presented in this section we conclude that there are some marker/alleles strongly associated with HASL, confirming that genetic factors play an important role in the adaptation of maize (and teosinte) to different altitudes.

S1-3. R Code

S1-3.1. **Function to calculate the distances between and within classes.** The following R text presents the function `dist.bet.wit`, a function to obtain a summary of the analysis, `sum.dist.bet.wit`, and a short example of the use of these functions. If you want to use the code, just paste it in your R window.

```
### --- Begins definition of function dist.bet.wit --- ###
dist.bet.wit <-
function(x, method, classes, norm.max=FALSE){
# dist.bet.wit
# Calculates the distances between and within classes
# and test if there is a significant difference using
# wilcox.test and t.test
# See: GM_100

# Octavio Martinez (omartine@langebio.cinvestav.mx)
# August 2015
# Computational Biology Lab ( http://computational.biology.langebio.cinvestav.mx/ )
# Langebio, Cinvestav, Irapuato.

# This software is distributed under the terms of the GNU General
# Public License "GPL-3" ( http://fsf.org/ ) 'Share and Enjoy.'

# Input
#x - data.frame or matrix (individuals are rows, characters are columns)
#method - Method to calculate the distance (used by "dist"; see help for that function)
#classes - A character vector (NOT a factor!) giving the class to which each individual belongs.
#norm.max - If true normalizes the distance by the maximum

# Output
# A list with the following components:
#D - An object of class "dist" giving the distances between all individuals (order by classes)
#bet - Vector with the distances between classes
#wit - Vector with the distances within classes
#d.with - a data.frame with statistics for the distances within classes per class
#W.t - Wilcoxon test for the distances between (bet) and within (wit)
#T.t - t test for the distances between (bet) and within (wit)
#the.call - Call made to the function.

if(nrow(x) != length(classes)) {
stop("Number of rows (individuals) in x must be the same than the length of classes!")}

# Sort x by the order of classes.
o.c <- order(classes) # Order in which the classes will be
classes <- classes[o.c]
x <- x[o.c,]

u.c <- unique(classes) # The distinct classes
n.c <- length(u.c) # Number of classes
if(n.c<2) stop("At least two classes are needed!")
```

```
# Obtain the matrix of distances
D <- dist(x, method=method)
if(norm.max) D <- D/max(D)

# Segregate the distances between and within
#individuals with the "classes" criteria.
d.m <- as.matrix(D)
t.i <- c(1:nrow(d.m)) # Index for the rows of distances
bet <- c()
wit <- c()
# n, min, median, mean, max, S
d.with <- data.frame(rep(NA, n.c), rep(NA, n.c), rep(NA, n.c),
rep(NA, n.c), rep(NA, n.c), rep(NA, n.c))
names(d.with) <- c("n", "min", "median", "mean", "max", "S")
attributes(d.with)$row.names <- c(1:n.c)

for(i in 1:n.c){ # for each class
i.wit <- t.i[classes == u.c[i]]
m.i <- max(i.wit)
i.bet <- t.i[t.i > m.i]
temp <- d.m[i.wit, i.wit]
temp2 <- temp[upper.tri(temp)]
wit <- c(wit, temp2)
d.with[i,] <- c(length(temp2), min(temp2), median(temp2), mean(temp2), max(temp2), sd(temp2))
attributes(d.with)$row.names[i] <- u.c[i]
bet <- c(bet, as.vector(d.m[i.wit, i.bet]))
}

# Perform the tests
w.t <- wilcox.test(bet, wit)
t.t <- t.test(bet, wit)

res <- list(D, bet, wit, d.with, w.t, t.t, match.call())
names(res) <- c("D", "bet", "wit", "d.with", "W.t", "T.t", "the.call")
res
}
### --- Ends definition of function dist.bet.wit --- ###

### --- Begins definition of function sum.dist.bet.wit --- ###
sum.dist.bet.wit <-
function(x){
# sum.dist.bet.wit
# Summary for objects produced by "dist.bet.wit"
# (see the remarks in "sum.dist.bet.wit")
# omartine@langebio.cinvestav.mx
# See: GM_100

# x has: c("D", "bet", "wit", "d.with", "W.t", "T.t", "the.call")
cat("Object produced by:\n")
print(x$the.call)
d <- length(x$D)
n <- 0.5 + sqrt(2*d + 0.25)
```

```
nc <- nrow(x$d.with)
nb <- length(x$bet)
nw <- length(x$wit)
P.s <- c(x$W.t$p.value, x$T.t$p.value)
names(P.s) <- c("Wilcoxon", "T test")
cat("\n",n,"individuals segregated into",nc,"classes.\n\nStatistics for the distances:\n\n")

# Statistics for classes:
stats <- rbind(summary(x$bet), summary(x$wit), summary(x$D))
stats <- cbind(c(nb, nw, d), stats, c(sd(x$bet), sd(x$wit), sd(x$D)))
attributes(stats)$dimnames[[2]][c(1,8)] <- c("n","S")
attributes(stats)$dimnames[[1]] <- c("Between: ", "Within: ","Total: ")
print(stats)

cat("\nP values for the tests of equality of means:\n")
print(P.s)
return(invisible(stats))
}
### --- Ends definition of function sum.dist.bet.wit --- ###

### Example of use of dist.bet.wit

# Simulate marker profiles for two populations.
# 5 individual profiles for 10 markers with distinct probabilities.
set.seed(1959) # Set the random seed to obtain the same results.

# Create first population
pop1 <- matrix(sample(c(0,1), size=50, replace=T, prob=c(.2,.8)),
nrow=5, ncol=10, dimnames=list(paste("P1I", c(1:5), sep=""),
paste("M", c(1:10), sep="")))

# Create second population
pop2 <- matrix(sample(c(0,1), size=50, replace=T, prob=c(.8,.2)),
nrow=5, ncol=10, dimnames=list(paste("P2I", c(1:5), sep=""),
paste("M", c(1:10), sep="")))

# Put both populations in the same matrix
both.pop <- rbind(pop1,pop2)

# To see the matrix:
both.pop

# Make the analysis by Euclidean distance
res.both.pop <- dist.bet.wit(x=both.pop, method="euclidean",
classes=rep(c("P1", "P2"), each=5))

# If you want to see the full object
res.both.pop

# Summary of the results:
sum.dist.bet.wit(res.both.pop)
```

```
# Object produced by:
# dist.bet.wit(x = both.pop, method = "euclidean", classes = rep(c("P1",
#     "P2"), each = 5))
#
#  10 individuals segregated into 2 classes.
#
# Statistics for the distances:
#
#            n  Min. 1st Qu. Median  Mean 3rd Qu.  Max.         S
# Between:  25 1.732   2.449  2.449 2.477   2.646 3.000 0.3267634
# Within:   20 1.000   1.732  2.000 1.896   2.000 2.449 0.3344439
# Total:    45 1.000   2.000  2.236 2.219   2.449 3.000 0.4381588
#
# P values for the tests of equality of means:
#     Wilcoxon       T test
# 6.344539e-06 7.244110e-07

# To see the distance within each group
res.both.pop$d.with
#     n      min median     mean      max         S
# P1 10 1.000000      2 1.856387 2.449490 0.4134223
# P2 10 1.414214      2 1.935045 2.236068 0.2485523
```

S1-3.2. **'All Marker Alleles' (AMA) algorithm.** The R function 'AMA' implements the 'All Marker Alleles' (AMA) algorithm to find a small collection of accessions containing all the marker/allele combinations. If you want to use the code, just paste it in your R window.

```
AMA <- function(mat){
# AMA - Select a set of accessions with all Marker/Allele combinations

# Octavio Martinez (omartine@langebio.cinvestav.mx)
# August 2015
# Computational Biology Lab ( http://computational.biology.langebio.cinvestav.mx/ )
# Langebio, Cinvestav, Irapuato.

# This software is distributed under the terms of the GNU General
# Public License "GPL-3" ( http://fsf.org/ ) 'Share and Enjoy.'

# REVISED 20 Ago. See: GM_108 (internal use)

# Obtains a set of optimal or near-optimal accessions with the
# complete set of all marker/aleles and a small size (number of accessions)

## INPUT
# mat - Matrix (or data.frame) with accessions as rows and columns as marker-alleles.
# Data in "mat" can be allele presence / absence (1, 0), frequencies or
# any numeric scale, but zero means the ABSENCE of the marker/allele.
# Not missing data (NA's) are allowed!
# BOTH rows (accessions) and columns (marker/allele combinations)
#must be named with DIFFERENT names

## OUTPUT. A list with the following components:
```

```
# "Set.names" - Names of the accessions (rows) that enter into the solving set
# "Set.nums" - Numbers corresponding to the rows that enter into the solving set
# "Richness" - Accumulated richness in each step of the algorithm
# "Ties" - Cases of ties (solved by using the "rearesst" accession)
# "MinMat" - Matrix containing the accessions that solve the problem.
# "rho" - Rareness coefficients for the original accessions.
# "nam.unique" - Names of marker/alleles that are "unique" (appears only in one accession)
# "acc.unique" - Names of accessions that have unique marker/allele combinations.
# "on.acc" - Original number of accessions in the input matrix (mat)
# "comment" - A comment that is printed as output.
# "call" - The call of the function.

# NOTE: Output is returned invisibly
#       (thus you must assign it to see particular components!)

the.call <- match.call()
sale <- function(){
comment <- paste("\nA set of ",length(G.num)," accessions contains all ",n.c,
" marker/alleles from the collection of ",n.r,
" accessions.\nThe set of selected accessions represents the ",
round(100*length(G.num)/n.r,2),"% of the original.\n", sep="")
res <- list(G.set, G.num, curr.rich, ties, mat[sort(G.num),], acc.rear, nam.unique,
acc.unique, on.acc, comment, the.call)
names(res) <- c("Set.names", "Set.nums", "Richness", "Ties", "MinMat", "rho", "nam.unique",
"acc.unique", "on.acc", "comment", "call")
cat(comment)
return(invisible(res))
}

if(min(mat)<0) stop("Input cannot have negative elements!")
to.test <- apply(mat, 2, mean)
if(length(mat[is.na(mat)==TRUE])>0) {
stop("Sorry; NA values are not accepted in the input matrix or data.frame!")}
if(length(to.test[to.test==0])>0){
warning(paste(length(to.test[to.test==0]),
"columns from the input add to zero; those will be eliminated!"))
mat <- mat[,to.test>0]
}
n.r <- nrow(mat)
on.acc <- n.r
n.c <- ncol(mat)
if(class(mat)=="data.frame") mat <- as.matrix(mat)
mal.mean <- apply(mat, 2, mean) # Means for each marker / allele
acc.rear <- NULL # This is the rearness coefficient to break ties
for(i in 1:n.r){
acc.rear <- c(acc.rear, sqrt(sum((mat[i,]-mal.mean)^2)/n.c))
}
names(acc.rear) <- c(1:n.r)
pa <- 1*(mat>0) # Is the matrix of presence / absence
acc.names <- attributes(mat)$dimnames[[1]]
names(acc.names) <- c(1:n.r)
mal.names <- attributes(mat)$dimnames[[2]]
```

```
names(mal.names) <- c(1:n.c)
G.set <- NULL
G.num <- NULL
col.sum <- apply(pa, 2, sum)
nam.unique <- names(col.sum[col.sum==1])
for(i in 1:length(nam.unique)){
temp <- pa[,attributes(pa)$dimnames[[2]]==nam.unique[i]]
G.set <- c(G.set, names(temp[temp==1]))
G.num <- c(G.num, c(1:n.r)[acc.names==names(temp[temp==1])])
}
G.set <- unique(G.set)
acc.unique <- G.set
G.num <- unique(G.num)
G.alleles <- rep(0, n.c)
for(i in 1:length(G.num)){
temp <- pa[G.num[i], ]
G.alleles <- G.alleles + temp
G.alleles <- 1*(G.alleles > 0)
}
 curr.set <- G.set
curr.rich <- sum(G.alleles)
acum.rich <- sum(G.alleles)
ties <- NULL
obj <- acum.rich
n.loop <- 1
if(sum(G.alleles) == n.c) sale()
while(obj<n.c){
n.loop <- n.loop + 1
gain <- rep(0, n.r)
names(gain) <- c(1:n.r)
for(i in 1:n.r){
gain[i] <- sum(1*((pa[i,]-G.alleles)>0))
}
m.rich <- max(gain)
if(m.rich==0) sale()
richest <- gain[gain==m.rich]
n.richest <- length(richest)
if(n.richest>1){
if(is.null(ties)){
ties <- paste(names(richest), collapse=", ")
names(ties) <- n.loop
} else {
ties <- c(ties, paste(names(richest), collapse=", "))
names(ties)[length(ties)] <- n.loop
}
richest.acc <- as.integer(names(richest))
rear.rich <- acc.rear[richest.acc]
max.rea <- max(rear.rich)
selected <- richest.acc[rear.rich==max.rea]
G.set <- c(G.set, acc.names[selected])
G.num <- c(G.num, selected)
# Capture the new alleles.
```

```
G.alleles <- G.alleles + pa[selected, ]
G.alleles <- 1*(G.alleles > 0)
} else {
selected <- as.integer(names(richest))
G.set <- c(G.set, acc.names[selected])
G.num <- c(G.num, selected)
G.alleles <- G.alleles + pa[selected, ]
G.alleles <- 1*(G.alleles > 0)
}
curr.rich <- c(curr.rich, sum(G.alleles))
obj <- sum(G.alleles)
}
names(G.set) <- NULL
sale()
}
```

References

[1] Yoav Benjamini and Yosef Hochberg. Controlling the false discovery rate: a practical and powerful approach to multiple testing. *Journal of the Royal Statistical Society. Series B (Methodological)*, pages 289–300, 1995.

[2] Ingwer Borg and Patrick JF Groenen. *Modern multidimensional scaling: Theory and applications*. Springer Science & Business Media, 2005.

[3] AHD Brown. Core collections: a practical approach to genetic resources management. *Genome*, 31(2):818–824, 1989.

[4] C. Clark Cockerham. Variance of gene frequencies. *Evolution*, 23(1):pp. 72–84, 1969.

[5] Herman De Beukelaer, Petr Smỳkal, Guy F Davenport, and Veerle Fack. Core hunter ii: fast core subset selection based on multiple genetic diversity measures using mixed replica search. *BMC bioinformatics*, 13(1):312, 2012.

[6] P Dubreuil, M Warburton, M Chastanet, D Hoisington, and A Charcosset. More on the introduction of temperate maize into europe: large-scale bulk ssr genotyping and new historical elements. *Maydica*, 51:281–291, 2006.

[7] Bradley Efron and Robert J Tibshirani. *An introduction to the bootstrap*. CRC press, 1994.

[8] B Everitt. Cluster analysis (2nd), 1980.

[9] Brian S Everitt. *The analysis of contingency tables*. CRC Press, 1992.

[10] Ronald A Fisher. Frequency distribution of the values of the correlation coefficient in samples from an indefinitely large population. *Biometrika*, pages 507–521, 1915.

[11] Sir Ronald Aylmer Fisher. *Statistical methods for research workers: by Ronald A. Fisher*. Oliver and Boyd, 1946.

[12] OH Frankel et al. Genetic perspectives of germplasm conservation. *Genetic manipulation: impact on man and society. Cambridge University Press, Cambridge*, pages 161–170, 1984.

[13] Mario González, Raul Rodríguez, Maria Elena Zavala, Juan L Jacobo, Fernando Hernández, Jorge Acosta, Octavio Martínez, and June Simpson. Characterization of mexican isolates of colletotrichum lindemuthianum by using differential cultivars and molecular markers. *Phytopathology*, 88(4):292–299, 1998.

[14] Major M Goodman, William L Brown, GF Sprague, JW Dudley, et al. Races of corn. *Corn and corn improvement. Third edition.*, pages 33–79, 1988.

[15] B Gouesnard, TM Bataillon, G Decoux, C Rozale, DJ Schoen, and JL David. Mstrat: An algorithm for building germ plasm core collections by maximizing allelic or phenotypic richness. *Journal of Heredity*, 92(1):93–94, 2001.

[16] Myles Hollander, Douglas A Wolfe, and Eric Chicken. *Nonparametric statistical methods*. John Wiley & Sons, 2013.

[17] M Iriany, N Widiyati, F Kasim, M Dahlan, et al. [evaluation of yield on hybrid maize in high land]. In *Seminar Nasional Hasil Pengkajian dan Penelitian Teknologi Pertanian Menghadapi Era Otonomi Daerah, Palu (Indonesia), 3-4 Nov 1999*. PSE, 1999.

[18] Paul Jaccard. *Nouvelles recherches sur la distribution florale*. 1908.

[19] Kyu-Won Kim, Hun-Ki Chung, Gyu-Taek Cho, Kyung-Ho Ma, Dorothy Chandrabalan, Jae-Gyun Gwag, Tae-San Kim, Eun-Gi Cho, and Yong-Jin Park. Powercore: a program applying the advanced m strategy with a heuristic search for establishing core sets. *Bioinformatics*, 23(16):2155–2162, 2007.

[20] Gustave Malécot. *The Mathematics of Heredity (Revised, edited and translated by Yermanos, DM).* Freeman, San Francisco, 1969.

[21] Yoshihiro Matsuoka, Yves Vigouroux, Major M Goodman, Jesus Sanchez, Edward Buckler, and John Doebley. A single domestication for maize shown by multilocus microsatellite genotyping. *Proceedings of the National Academy of Sciences*, 99(9):6080–6084, 2002.

[22] Peter McCullagh and John A Nelder. *Generalized linear models*, volume 37. CRC press, 1989.

[23] SA Mohammadi and BM Prasanna. Analysis of genetic diversity in crop plants?salient statistical tools and considerations. *Crop Science*, 43(4):1235–1248, 2003.

[24] Masatoshi Nei. Analysis of gene diversity in subdivided populations. *Proceedings of the National Academy of Sciences*, 70(12):3321–3323, 1973.

[25] R Core Team. *R: A Language and Environment for Statistical Computing.* R Foundation for Statistical Computing, Vienna, Austria, 2013. ISBN 3-900051-07-0.

[26] Sohini Ramachandran, Omkar Deshpande, Charles C Roseman, Noah A Rosenberg, Marcus W Feldman, and L Luca Cavalli-Sforza. Support from the relationship of genetic and geographic distance in human populations for a serial founder effect originating in africa. *Proceedings of the National Academy of Sciences of the United States of America*, 102(44):15942–15947, 2005.

[27] M Humberto Reyes-Valdés, Amalio Santacruz-Varela, Octavio Martínez, June Simpson, Corina Hayano-Kanashiro, and Celso Cortés-Romero. Analysis and optimization of bulk DNA sampling with binary scoring for germplasm characterization. *PloS one*, 8(11):e79936, January 2013.

[28] Robert R Sokal. A statistical method for evaluating systematic relationships. *Univ Kans Sci Bull*, 38:1409–1438, 1958.

[29] Robert R Sokal and F James Rohlf. *Biometry: the principles andpractice of statistics in biological research.* WH. Freeman& Co., San Francisco., 1995.

[30] Chris Thachuk, José Crossa, Jorge Franco, Susanne Dreisigacker, Marilyn Warburton, and Guy F Davenport. Core hunter: an algorithm for sampling genetic resources based on multiple genetic measures. *BMC bioinformatics*, 10(1):243, 2009.

[31] Yves Vigouroux, Jeffrey C Glaubitz, Yoshihiro Matsuoka, Major M Goodman, Jesús Sánchez, and John Doebley. Population structure and genetic diversity of new world maize races assessed by dna microsatellites. *American Journal of Botany*, 95(10):1240–1253, 2008.

[32] M Vuylsteke, R Mank, B Brugmans, P Stam, and M Kuiper. Further characterization of aflp® data as a tool in genetic diversity assessments among maize (zea mays l.) inbred lines. *Molecular Breeding*, 6(3):265–276, 2000.

[33] Marilyn L Warburton, Xia Xianchun, Jose Crossa, Jorge Franco, Albrecht E Melchinger, Matthias Frisch, Martin Bohn, and David Hoisington. Genetic characterization of cimmyt inbred maize lines and open pollinated populations using large scale fingerprinting methods. *Crop Science*, 42(6):1832–1840, 2002.

[34] Daniela M Witten and Robert Tibshirani. A framework for feature selection in clustering. *Journal of the American Statistical Association*, 105(490), 2010.

[35] Sewall Wright. The genetical structure of populations. *Annals of eugenics*, 15(1):323–354, 1949.
