# Supplementary material for: An SSR‐based approach incorporating a novel algorithm for identification of rare maize genotypes facilitates criteria for landrace conservation in Mexico
Source: Ecol Evol. 2017 Feb 10;7(6):1680–90. doi: 10.1002/ece3.2754 (PMC5355182; doi:10.1002/ece3.2754)
Supplement: Supplementary file 2 [file ECE3-7-1680-s002.pdf]

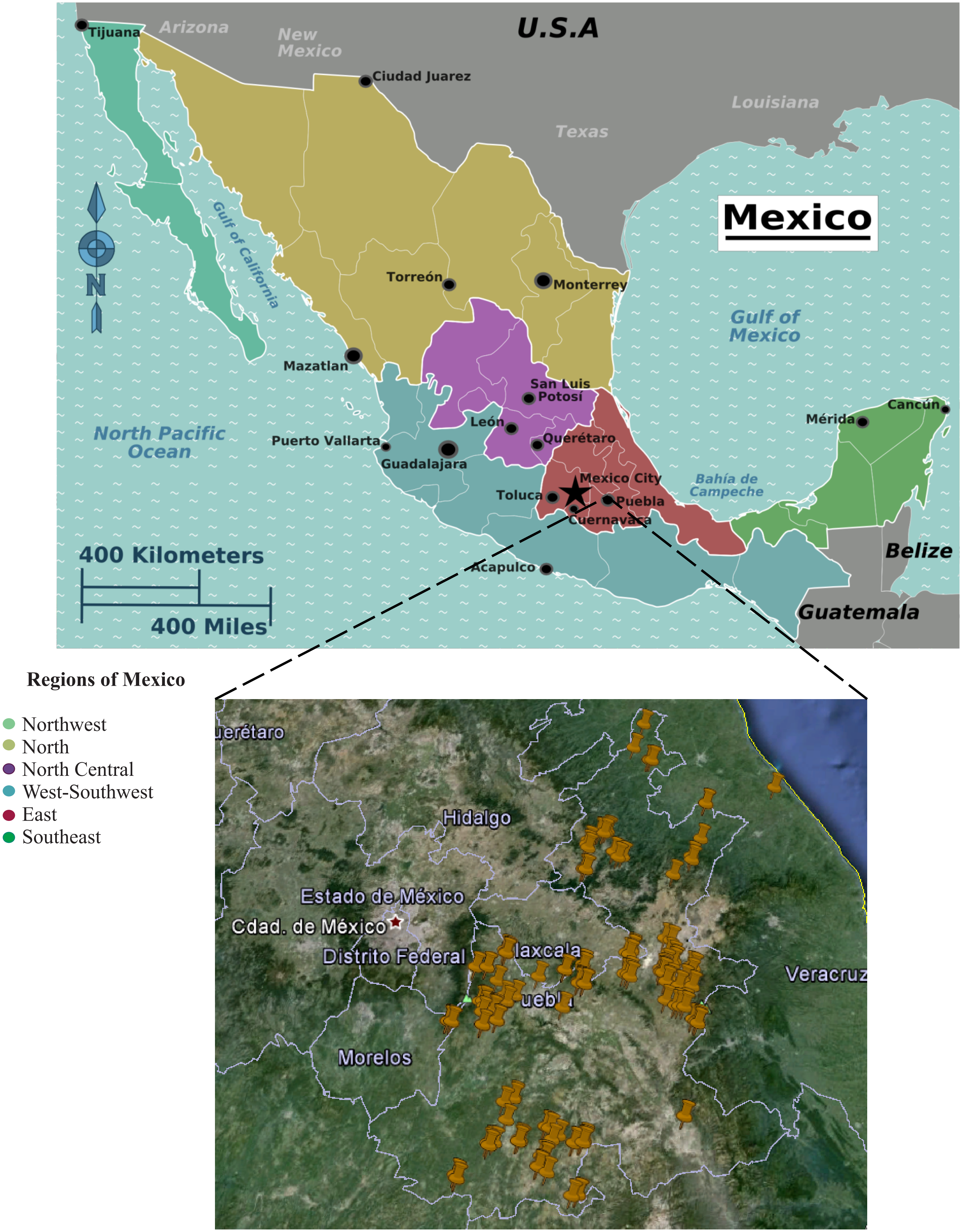

Supplementary Figure S1. Sampling locations of *Zea mays* L. accessions from Puebla State. Upper panel shows the location of Puebla State within Mexico-colors indicate the different regions of Mexico. Lower panel shows a close up aerial view of sampling sites. Map of Mexico was taken from https://www.worldofmaps.net/nordamerika/mexiko/karte-regionen-mexiko.htm
